# Alpha-lipoic acid alleviates cognitive deficits in transgenic APP23/PS45 mice through a mitophagy-mediated increase in ADAM10 α-secretase cleavage of APP

**DOI:** 10.1186/s13195-024-01527-3

**Published:** 2024-07-19

**Authors:** Jie Zhang, Yanshuang Jiang, Xiangjun Dong, Zijun Meng, Liangye Ji, Yu Kang, Mingjing Liu, Weihui Zhou, Weihong Song

**Affiliations:** 1 Chongqing Key Laboratory of Translational Medical Research in Cognitive Development and Learning and Memory Disorders, Ministry of Education Key Laboratory of Child Development and Disorders, National Clinical Research Center for Child Health and Disorders, International Science and Technology Cooperation Base of Child Development and Critical Disorders, Children’s Hospital of Chongqing Medical University, Chongqing, China; 2 Institute of Aging, Key Laboratory of Alzheimer’s Disease of Zhejiang Province, Zhejiang Provincial Clinical Research Center for Mental Disorders, School of Mental Health and the Affiliated Wenzhou Kangning Hospital, Wenzhou Medical University, Wenzhou, Zhejiang China; 3 Oujiang Laboratory (Zhejiang Lab for Regenerative Medicine, Vision and Brain Health), Wenzhou, Zhejiang 325001 China

**Keywords:** AD, ALA, ADAM10, Mitophagy, Cognitive deficits

## Abstract

**Background:**

Alpha-lipoic acid (ALA) has a neuroprotective effect on neurodegenerative diseases. In the clinic, ALA can improve cognitive impairments in patients with Alzheimer’s disease (AD) and other dementias. Animal studies have confirmed the anti-amyloidosis effect of ALA, but its underlying mechanism remains unclear. In particular, the role of ALA in amyloid-β precursor protein (APP) metabolism has not been fully elucidated.

**Objective:**

To investigate whether ALA can reduce the amyloidogenic effect of APP in a transgenic mouse model of AD, and to study the mechanism underlying this effect.

**Methods:**

ALA was infused into 2-month-old APP23/PS45 transgenic mice for 4 consecutive months and their cognitive function and AD-like pathology were then evaluated. An ALA drug concentration gradient was applied to 20E2 cells in vitro to evaluate its effect on the expression of APP proteolytic enzymes and metabolites. The mechanism by which ALA affects APP processing was studied using GI254023X, an inhibitor of A Disintegrin and Metalloproteinase 10 (ADAM10), as well as the mitochondrial toxic drug carbonyl cyanide m-chlorophenylhydrazone (CCCP).

**Results:**

Administration of ALA ameliorated amyloid plaque neuropathology in the brain tissue of APP23/PS45 mice and reduced learning and memory impairment. ALA also increased the expression of ADAM10 in 20E2 cells and the non-amyloidogenic processing of APP to produce the 83 amino acid C-terminal fragment (C83). In addition to activating autophagy, ALA also significantly promoted mitophagy. BNIP3L-knockdown reduced the mat/pro ratio of ADAM10. By using CCCP, ALA was found to regulate BNIP3L-mediated mitophagy, thereby promoting the α-cleavage of APP.

**Conclusions:**

The enhanced α-secretase cleavage of APP by ADAM10 is the primary mechanism through which ALA ameliorates the cognitive deficits in APP23/PS45 transgenic mice. BNIP3L-mediated mitophagy contributes to the anti-amyloid properties of ALA by facilitating the maturation of ADAM10. This study provides novel experimental evidence for the treatment of AD with ALA.

**Graphical abstract:**

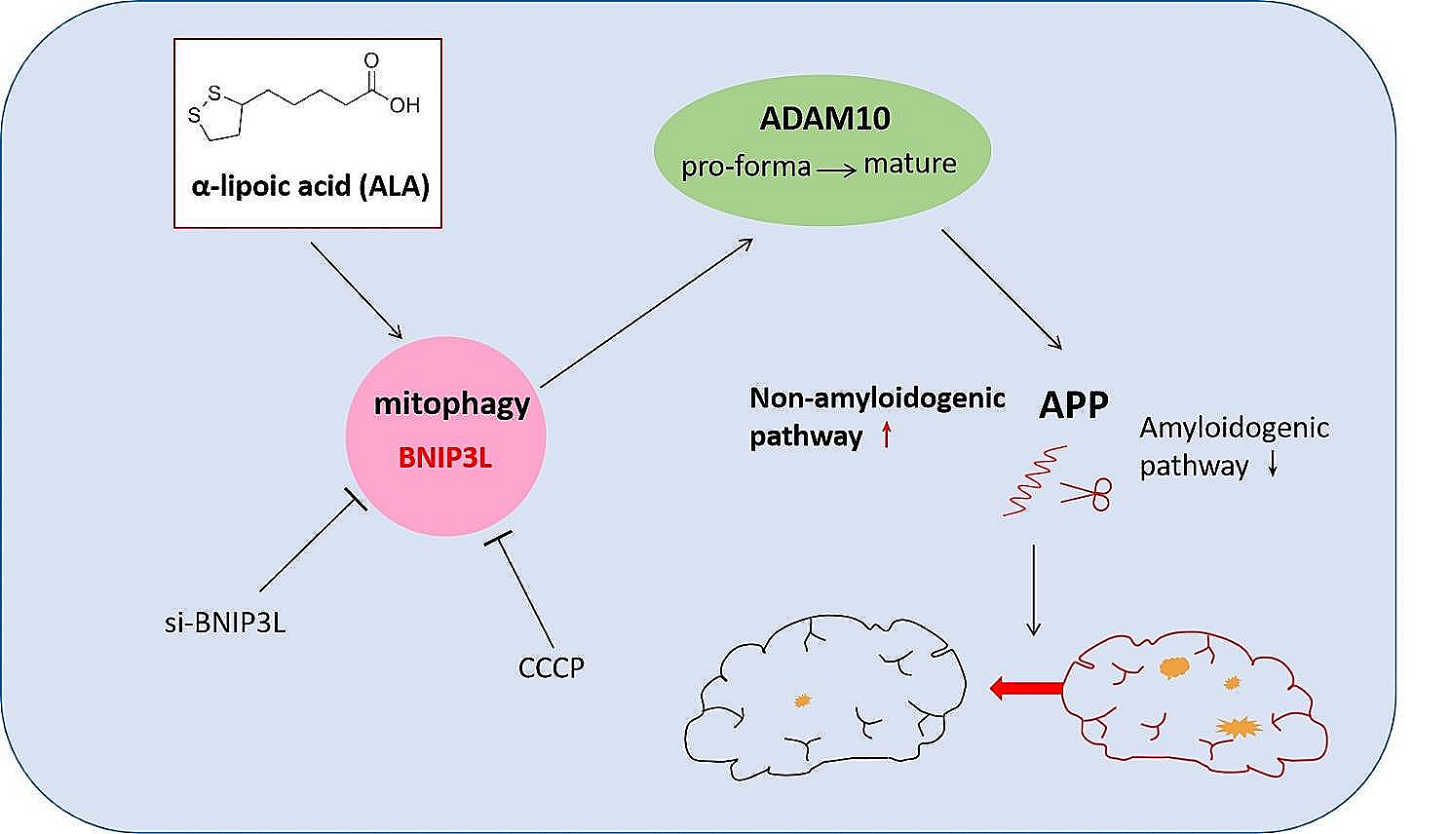

**Supplementary Information:**

The online version contains supplementary material available at 10.1186/s13195-024-01527-3.

## Introduction

AD is the most prevalent neurodegenerative disorder and has an insidious onset. It is characterized by a progressive decline in learning and memory, together with cognitive impairment [1]. Neurotic plaques, neurofibrillary tangles, and neuronal loss are the characteristic neuro-pathologies of AD, although the mechanism underlying AD pathogenesis remains unknown [2]. Amyloid plaques are comprised mostly of amyloid-β protein (Aβ) and are generated by the sequential cleavage of APP at β- and γ-secretase cutting sites. The amyloid cascade hypothesis is considered to be the predominant cause of AD [2–4]. Many experimental studies as well as clinical data have shown that reducing the production and deposition of Aβ have potential therapeutic benefits in AD [5–9]. APP is a key cell adhesion molecule involved with various events during neuronal development, including synaptogenesis, synaptic plasticity, neurite outgrowth, growth cone pathfinding, and migration [10–12]. However, the metabolism of APP is very complex, with studies showing that it can be cleaved through a non-amyloid pathway mediated by α-secretase, as well as through an amyloid pathway mediated by β-secretase [13, 14]. APP is mainly cleaved in neurons by ADAM10, the major α-secretase, thereby releasing C83 and a secreted N-terminal APPα (sAPPα) [15, 16]. C83 is further cleaved by γ-secretase to generate the APP intracellular domain and a 3 kDa product [17]. sAPPα has neurotrophic and neuroprotective properties [18–22]. Beta-site amyloid β precursor protein cleaving enzyme (BACE1) is a β-secretase. Under normal conditions, β-cleavage at the Glu^11^ site (β’-site) of APP releases C89 and a truncated Aβ, Aβ_11−40/42_. In addition to the Glu^11^ site, BACE1 also cleaves APP at the Asp^1^ site (β-site) under pathological conditions to generate C99 and intact Aβ, mainly Aβ_1−40/42_ [23–26]. BACE1 functions as an initiator of amyloidogenic APP processing at the Asp^1^ site, while Aβ_1−40/42_ plays a central role in the pathogenesis of AD [27]. β-secretase competes with α-secretase for APP. Researchers have therefore sought novel breakthroughs in AD therapy through inhibition of BACE1 cleavage or promotion of ADAM10 cleavage [27].

ALA is the natural cofactor for pyruvate dehydrogenase and α-ketoglutarate dehydrogenase, and can easily penetrate the blood-brain barrier [28–30]. ALA has high safety and minimal toxicity due to its antioxidant characteristics and iron chelation ability. It has been used in the treatment of numerous chronic diseases, including diabetes and associated peripheral neuropathy [31, 32]. Importantly, an incidental finding in clinical trials was that ALA could improve cognitive function in AD [33]. Furthermore, human studies have shown that ALA levels decline significantly with age [34]. Other evidence supports a neuroprotective effect of ALA in patients with AD and age-related dementias [35]. Most studies aimed at the prevention and treatment of AD with ALA have focused on its antioxidant and anti-inflammatory properties, as well as its beneficial effects on glucose metabolism [36–39]. However, it has yet to be determined whether ALA can directly affect APP metabolism, and especially the pathologic changes in Aβ.

Mitophagy is a selective form of autophagy that plays a crucial role in maintaining mitochondrial homeostasis [40]. The membrane structure of autophagosome is derived from specific cell organelles or structures, such as the endoplasmic reticulum (ER) [41, 42]. The formation of autophagosomes involves membrane rearrangement of organelles and the lysosome degradation pathway. Moreover, the metabolic pathway of APP is related to the transportation and maturation of APP and its cutting enzymes α- and β-secretases in the membrane structure of cells. Their localization on the sub-organelles facilitates the interaction between the cutting enzymes and APP substrates [13]. The majority of APP localizes in the Golgi complex with only a small proportion of APP is detected at the cell surface [43, 44]. Over 50% of APP is internalized within 10 min and classified into early endosomes, where one portion is recycled back into the plasma membrane (PM) and another is targeted to the lysosome for degradation [44–48]. The pro-forma ADAM10 undergoes a series of folding and modification in the ER before entering the Golgi apparatus for further glycosylation modification into the mature form [49–51]. Finally, mature ADAM10 is transported to the PM to perform its α-secretase activity [52]. Furthermore, both nascent APP and BACE1 mature through the constitutive secretory pathway from the ER to PM [53]. Therefore, the metabolic pathway of APP is closely linked to changes in the membrane structure and to lysosomal degradation. However, there is currently no evidence linking the APP metabolic pathway to mitophagy in AD. Studies have shown that ALA can improve mitochondrial function, as well as being involved in the regulation of autophagy in neurodegenerative diseases. However, it is still not known whether ALA participates in mitophagy during AD.

The current study found that ALA may have a promoting effect on the activity of ADAM10, thereby alleviating memory deficits in APP23/PS45 transgenic mice. Surprisingly, ALA was found to regulate BNIP3L-mediated mitophagy, thereby having a positive effect on the process of APP cleavage.

## Materials and methods

### Animals

This study used APP23/PS45 double transgenic mice with a background of C57BL/6 as animal models of AD. The APP23/PS45 mice were generated by cross-breeding APP23 and PS45 mice in our lab as previously described [54]. These genetically modified mice carry specific mutations in their DNA, including human APP751 cDNA with the Swedish double mutation at positions 670/671 (KM3NL) in APP23 transgenic mice, and presenilin-1 (PS1) cDNA with a human familial AD-associated G384A mutation in PS45 transgenic mice. The two cDNAs are under the control of the murine Thy1.2 promoter. All experiments were conducted in accordance with the ethical guidelines of the Animal Centre at the Children’s Hospital of Chongqing Medical University. The mice were housed in a controlled 12 h light/dark cycle environment with sufficient food and water. WT + Veh, AD + Veh and AD + ALA group mice were subjected to administration at 2 months of age. AD + ALA group mice were treated with ALA (5 mg/kg), WT + Veh and WT mice were treated with the same volume of vehicle solution via intraperitoneal injection once daily for 4 months. ALA was freshly diluted with sterile phosphate-buffered saline (PBS).

### Mouse genotyping

Genotypes for the APP23/PS45 and WT mice were confirmed by Polymerase Chain Reaction (PCR) amplification of genomic DNA extracted from tail tissues. The primer sequences were as follows: Thy1.2-forward (5’ -CACCACAGAATCCAAGTCGG-3’), APP23-reverse (5’-CTTGACGTTCTGCCTCTTCC-3’), and PS1-reverse (5’-ATCACAGCCAAGATGAGC-3’). The parameters for PCR amplification were set as follows: 2 µL genomic DNA, 0.5 µL primer, 10 µL Taqmix and 6.5 µL ddH_2_O into 20 µL premix system, predenaturation at 94 °C for 5 min, denaturation at 94 °C for 40 s, annealing at 56 °C for 40 s, and extension at 72 °C for 1 min (40 cycles) with final thorough extension at 72 °C for 10 min. The PCR products were loaded onto 1.5% agarose gels containing Goldview nucleic acid dye and electrophoresed at 120 V for 25 min. The separated DNA fragments were visualized in a gel imaging system and the resulting electrophoretic images were captured and saved for further analysis.

### Drugs

ALA was purchased from the supplier (Chengdu Brilliant Pharmaceutical Co., Ltd.) and diluted with PBS. GI254023X and CCCP were purchased from Selleck. Chloroquine (CQ) was purchased from Sigma. GI254023X and CQ were dissolved in DMSO.

### Antibodies

Rabbit anti-APP C-terminal polyclonal antibody C20 (1:1000) was used to detect APP and its C-terminal fragments. Anti-ADAM10 (1:1000, #ab124695) and anti-PS1 (1:1000, #ab15458) were purchased from Abcam. Anti-sAPPα 6E10 (1:1000, SIG-39,320) was purchased from Biolegend. Anti-ADAM17 (1:2000, #6978), anti-BACE1(1:1000, #5606), anti-P62 (1:1000, #39,749), anti-LC3 (1:1000, #12,741), and anti-BNIP3L (1:1000, #12,396) were purchased from CST. Anti-β-actin (1:10000, #81115-1-RR) and anti-GAPDH (1:50000, #60004-1-Ig) were purchased from Proteintech.

### Open field test

Each mouse was placed individually in an opaque chamber (30 × 30 × 30 cm) with an open top and allowed to roam freely for 10 min. ANY-maze software was used to track the activity of mice. The middle 100 cm^2^ area was arbitrarily defined as the central area, and the remaining area as the peripheral area. The number of mice entering the central and peripheral areas were counted, with the percentage entering the central area used as the evaluation index: percentage of central area entries/total entries into central area and peripheral area.

### Elevated plus maze

The elevated plus maze is a ‘cross’-shaped maze placed at a height of 60 cm above ground and consisting of two open arms (35 × 5 cm) and two closed arms of the same size and with 15 cm high walls. Each mouse was placed in the central area of the maze facing the same open arm and allowed to explore for 5 min without any inducing stimulus. Exploration behaviours were monitored and analysed with ANY-maze software (ANY maze, Stoelting). Entries into open arms or closed arms were calculated as an exploration practice, with the mouse head completely entering an open arm or a closed arm from the central area. The proportion of entries into open arms was used as an indicator to evaluate the anxiety level: percentage of time into open arms/total entries into open arms and closed arms.

### Y maze

The Y maze test is a behavioural test used to monitor short-term spatial memory. The experimental apparatus consists of A, B and C arms. At the beginning of each test, the mouse was placed in the same position near the distal end of arm A and with its head facing the central area, whereupon it was allowed to explore freely for 8 min. The camera and ANY-maze software recorded and monitored the mouse’s exploration path. The behaviour of its head completely entering into arm A, B or C from the central region and then protruding out was defined as one entry. Entering the three arms consecutively was counted as an effective alternation, and the maximum number of alternations was calculated by subtracting 2 from the total entries. The spontaneous alternations preference (SAP) score (effective number of alternations/maximum number of alternations) was used as a monitored index for the short-term spatial memory ability of mice.

### Morris water maze

This test was performed to detect spatial learning and memory in mice at the age of 6-months, as previously described [55, 56]. WT mice and APP23/PS45 mice with or without ALA treatment were subjected to the classic Morris water maze schedule. This consisted of a visible platform test on day-1, a 4-day hidden platform test from day 2 to 5, and finally a 24 h probe trial. In the visible and hidden platform tests, each mouse was trained by 5 continuous trials, with an inter-trial interval of 90 min. Mice that found the platform during the 60 s trial were allowed to stay on the platform for 5 s, while mice that could not locate the platform during a maximum of 60 s were artificially guided there and then rested for 20 s. In the probe trial the platform was withdrawn, thus forcing each mouse to search for the platform for 60 s. The tracks taken by mice were recorded by ANY-maze software, and the path length, escape latency, and passing times through the platform or SW3 quadrant were measured. These data were analysed by two-way ANOVA with post hoc LSD test.

### Immunohistochemical staining

One half of the mouse brain was infused with PBS and then fixed in freshly prepared 4% paraformaldehyde (pH 7.4) for one week, dehydrated in 30% sucrose solution for 3 days, and subsequently sectioned into 30 μm-thick coronal slices using a cryostat. To induce DNA denaturation, the slices were incubated with 88% formic acid for 15 min, and residual peroxidase activity was removed by incubating with 3% H_2_O_2_ for 30 min. After incubating with 5% skim milk for 2 h, the slices were incubated overnight at 4℃ with 4G8 primary antibody (diluted 1:500). Plaques were visualized by the ABC and DAB methods, and images were recorded with a whole slide scanner under 40× magnification.

### Aβ40/42 ELISA assay

Cell culture media or APP23/PS45 double transgenic mouse brain tissue homogenates were collected as recommended by the ELISA Technical Guide (***thermofisher.com***). Protease inhibitors (Roche, Basel, Switzerland) were added to the media or homogenates to prevent serine proteases from degrading Aβ peptides. The level of Aβ40/42 was determined using an Aβ40/42 ELISA Kit (KHB3481/KHB3544, Invitrogen). A microplate reader (Bio Tek Synergy H1, Winooski, USA) was used to measure the optical density at 450 nm. The concentration of Aβ40/42 peptides in samples was estimated according to the optical density values and a standard curve.

### Western blot assay

Proteins from brain tissues or cells were extracted with a radioimmunoprecipitation assay (RIPA) buffer supplemented with a protease inhibitor. Media from cultured cells were concentrated into a powder in a vacuum freezer and then suspended in the RIPA lysate. The protein concentration of each sample was quantified using the BCA method. An equal mass of protein (30–60 µg) from each sample was diluted with 5×SDS sample buffer and ddH_2_O into the same volume. After denaturation at 100℃, the samples were separated by electrophoresis using 10% or 12.5% Tris-glycine SDS-PAGE gels. Subsequently, the proteins were transferred to PVDF membranes and then blocked with 5% skim milk for 2 h. For immunoblotting analysis, the membranes were incubated overnight at 4℃ with diluted primary antibodies. Finally, horseradish peroxidase (HRP)-labelled goat anti-rabbit/mouse IgG (Proteintech, 1:10000) was used to detected the specific target protein. Blots were visualized using GENE GNOME imager (Syngene, UK) with clarity western ECL substrate (Bio-Rad).

### Assay for ADAM10 activity

The assay for ADAM10 activity was performed according to the manufacturer’s instructions for the SensoLyte^®^ 520 ADAM10 Activity Assay Kit (AS-72,226, ANASPEC). Brain tissues or cells were fully homogenized in pre-chilled assay buffer. The supernatant was then obtained by centrifugation at 10,000×g for 15 min at 4℃. An aliquot (50 µL) of supernatant from each sample was then added into the corresponding well of a 96-well microplate with a black, flat bottom plate and non-binding surface. Three replicate wells were used for each sample. A positive control solution (50 µL) containing purified ADAM10 enzyme, or a negative control solution containing only assay buffer, were added to the control wells. A 10 µM 5-FAM reference standard was diluted with assay buffer by 2-fold serial dilution to obtain concentrations of 5, 2.5, 1.25, 0.625, 0.312, 0.156, and 0 µM. Next, 50 µL of these serially diluted 5-FAM reference solutions were added per well. Freshly prepared ADAM10 substrate (5-FAM/QXLTM 520) was diluted 100-fold in pre-chilled assay buffer, and 50 µL was then added to each well to start the enzymatic reaction. The fluorescence intensity at Ex/Em = 490 nm/520 nm was measured immediately and continuously, with the data recorded every 10 min for 2 h. The concentration of enzymatic reaction product was calculated by reference to the 5-FAM fluorescence standard curve, and the data for each sample was plotted as a curve of the relative 5-FAM concentration versus time.

### Cell culture

HEK293 cells stably transfected with the Swedish mutant APP695 plasmid were referred to as the 20E2 cell line. The 20E2 cells were cultured in DMEM media (50 µg/mL G418) supplemented with 10% fetal bovine serum (Gibco), and maintained at 37℃ in an incubator with 5% CO_2_.

### Tandem mRFP-eGFP-LC3 assay

The mRFP-eGFP-LC3 double fluorescent plasmid serves as a vector for autophagy detection. It contains the rat LC3B autophagy gene encoding the mRFP-eGFP tandem fluorescent-tagged LC3 (tfLC3). Prior to fusion with lysosomes, tfLC3 exhibits both eGFP and mRFP signals and displays yellow fluorescence. Upon fusion of autophagosomes with lysosomes, eGFP fluorescence is quenched under acidic conditions, thus enabling specific detection of LC3 protein labeled with mRFP [57]. Chloroquine (CQ), a classical autophagy inhibitor, impedes the fusion between autophagosomes and lysosomes as well as the degradation of lysosomal proteins by increasing the lysosomal pH levels [58]. This reporter plasmid combined with CQ is of general utility for analysing the autophagosome maturation process. This method was employed to investigate the impact of ALA on autophagy flux in 20E2 cells. The specific experimental steps were as follows: 20E2 cells transfected with mRFP-eGFP-LC3 plasmid were evenly seeded onto a 15 mm diameter confocal microscopy dish. At 60% confluence, the cells were transfected with dual fluorescence mRFP-eGFP-LC3 plasmid and then treated with ALA (400 µM) or CQ (25 µM) for 24 h. Finally, the cells were fixed with 4% paraformaldehyde and stained with 1 µg/mL DAPI (Sigma-Aldrich) in PBS. Images were obtained using a laser confocal microscope (Nikon C2 Plus, Japan). For the quantification of autophagic flux, red and yellow LC3 dots in 5 replicates were quantified by counting > 30 cells.

### Transmission electron microscopy (TEM)

TEM was used to observe the autophagic flux in 20E2 cells following ALA treatment for 24 h. More than 1 × 10^6^ cells were centrifuged into a compact cellular mass, treated with 2.5% glutaraldehyde overnight at 4℃, fixed with 1% (w/v) osmium tetroxide for 2 h, then dehydrated with graded concentrations of ethanol (50%, 70%, 80%, 90%, 95%, and 100%) and 100% acetone for 20 min each time. The samples were embedded in Epon812 epoxy resin overnight at 70℃, and then sliced into 1 μm ultrathin sections with a Reichert ultra-thin microtome. The sections were stained with lead citrate and a 50% ethanol saturated solution of uranyl acetate for 15 min each, and finally observed by TEM.

### Mito-tracker staining

Mitochondrial content was measured using Mito-Tracker Red CMXRos (C1049B, Beyotime Biotech Co.). Cells were stained with a 200 nM working solution at 37℃ for 30 min, and then incubated with fresh media containing 1× Hoechst for 5 min. Images were collected at 405 nm and 561 nm excitation wavelengths.

### Assay for mitochondrial membrane potential (MMP)

MMP was detected using the tetraethylbenzimidazolylcarbocyanine iodide dye (JC-1) dual fluorescence probe (C2006, Beyotime Biotech Co.). When MMP is elevated, JC-1 formes J-aggregates in the mitochondrial matrix and emits red fluorescence. Conversely, when MMP is reduced, JC-1 fails to accumulate in the mitochondrial matrix and exists as a monomer that emits green fluorescence. The depolarization of mitochondria is assessed by the ratio of red/green fluorescence intensity. CCCP, a reversible proton-gradient uncoupling agent, induces rapid mitochondrial depolarization and decreases the MMP. Positive control cells were first treated with 10 µM CCCP for 20 min, then cells in each group were incubated with JC-1 (1×) in a cell incubator at 37℃ for 20 min. Subsequently, cells were washed twice with JC-1 staining buffer (1×), cultured in new media containing Hoechst (1×), and imaged with a fluorescence microscope. The fluorescence intensity of JC-1 monomer (green) and J-aggregate (red) was detected at excitation wavelengths of 488 nm and 561 nm, respectively, with the red/green fluorescence values used to evaluate MMP.

### siRNA transfection

20E2 cells were transfected with 50 nM BNIP3L specific siRNA or a negative control siRNA sequence (WZ Biosciences Inc. China) using Lipofectamine 2000 transfection reagent (11668, Invitrogen). The sequences of siRNA as followed: BNIP3L si-1 forward sense 5’-CAGTCAGAAGAAGAAGTTGTA-3’, BNIP3L si-1 reverse sense 5’-TACAACTTCTTCTTCTGACTG-3’; BNIP3L si-2 forward sense 5’-GCTAGGCATCTATATTGGAAA-3’, BNIP3L si-2 reverse sense 5’-TTTCCAATATAGATGCCTAGC-3’; BNIP3L si-3 forward sense 5’-CCCTAAACGTTCTGTGTCTTT-3’, BNIP3L si-3 reverse sense 5’-AAAGACACAGAACGTTTAGGG-3’; negative control siRNA forward sense 5’-TTCTCCGAACGTGTCACGT-3’, negative control siRNA reverse sense 5’-ACGTGACACGTTCGGAGAA-3’.

### Statistical analysis

The statistical analysis was conducted using GraphPad Prism 8.0 and SPSS 22.0 software. Student’s *t* test was employed for comparing two groups, while One-way ANOVA was used for comparisons among multiple groups. Additionally, Two-way ANOVA with post hoc LSD test was utilized to assess differences in data from the spatial learning trials of Morris water maze and ADAM10 enzyme activity. Data were presented as the mean ± SEM. Values of *P* < 0.05 were considered statistically significant, and ns means not significant.

## Results

### ALA attenuated cognitive deficits in APP23/PS45 transgenic mice

**Fig. 1:**
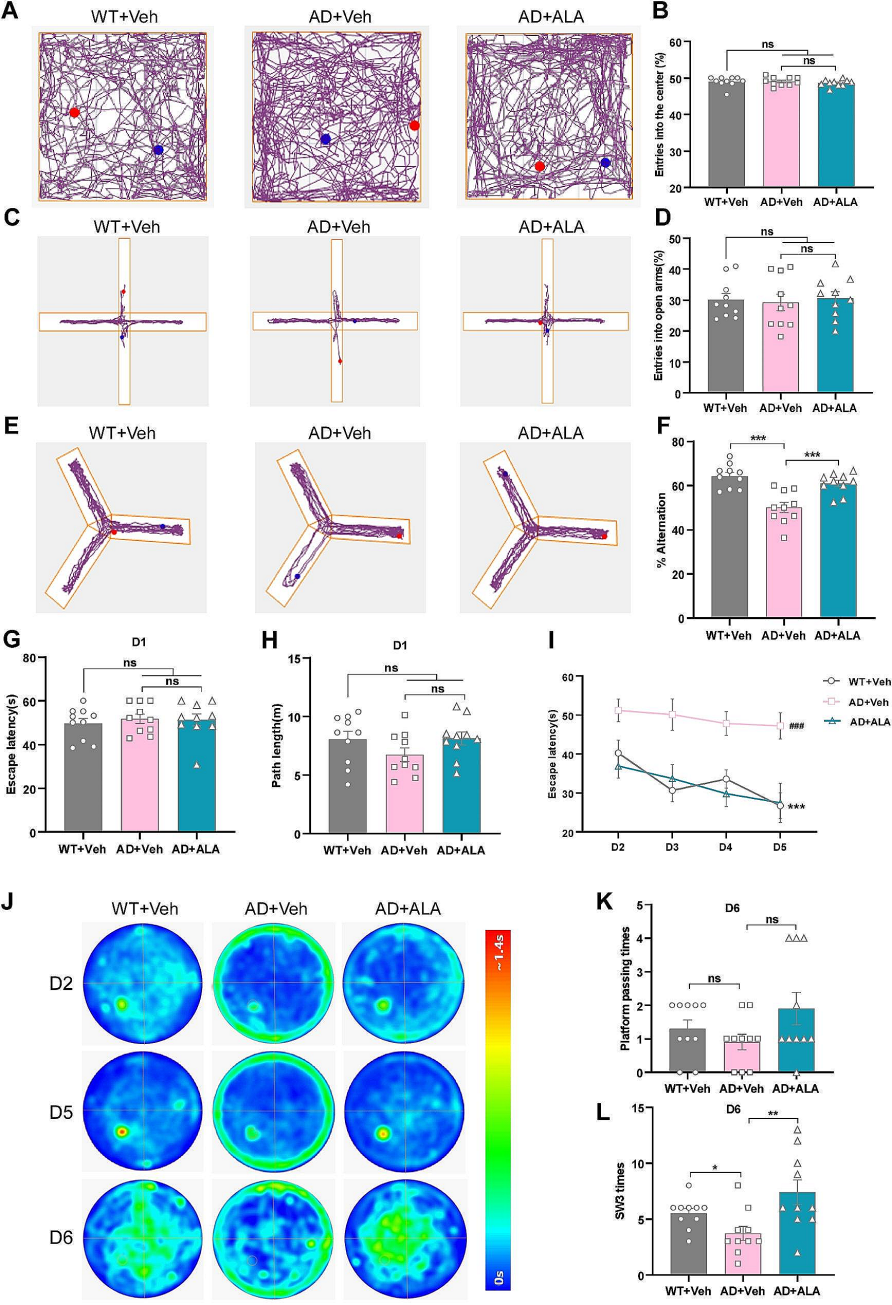
Behavioural experiments showed that cognitive deficits in APP23/PS45 transgenic mice were alleviated by ALA. **(A)** Representative movement trajectories of mice in open field test. **(B)** Percentages of entries into the central area by WT + Veh, AD + Veh and AD + ALA mice. **(C)** Representative movement trajectories of mice in elevated plus maze. **(D)** Percentages of entries into open arms from three groups. **(E)** Representative movement trajectories of mouse in Y maze. **(F)** Scores for SAP. **(G, H)** Escape latency and path length to a visual platform in the visible platform period. **(I)** AD + ALA group mice showed shorter latency to escape onto the hidden platform during day 2 to 5 compared with the AD + Veh group (AD + Veh vs. WT + Veh, ^###^*P* < 0.001; AD + ALA vs. AD + Veh, ***P < 0.001). **(J)** Motion track heat maps from three groups tested on day 2, 5 and 6. **(K, L)** During the no platform period, the entries into SW3 quadrant for AD + ALA mice were significantly more than the AD + Veh mice. *n* = 10. *P < 0.05, **P < 0.01, ***P < 0.001

To investigate whether ALA affected anxiety and depression, three groups (WT + Veh, AD + Veh, AD + ALA) of 6-month old mice underwent the open field test and elevated plus maze. In the open field test, no significant differences in the percentage of entries into the central area were observed between the three groups (*P* > 0.05; Fig. 1A, B). Moreover, the three groups showed similar results for the exploration of open arms in the elevated plus maze (*P* > 0.05; Fig. 1C, D). Hence, these tests confirmed that ALA administration did not affect anxiety and depression-like behaviours in APP23/PS45 transgenic mice.

SAP in the Y maze was used as an index of short-term spatial memory. The SAP score for AD + Veh mice was significantly lower than that of WT + Veh mice (AD + Veh, 50.08% ± 2.34% vs. WT + Veh, 64.29% ± 1.69%; *P* < 0.001; Fig. 1E, F). ALA treatment significantly increased the SAP score of the AD model (AD + ALA, 62.86% ± 1.47% vs. AD + Veh, 50.08% ± 2.34%; *P* < 0.001; Fig. 1E, F). The results of the Y maze indicated the short-term spatial memory ability of APP23/PS45 transgenic mice was impaired compared to WT mice. Interestingly, ALA treatment reversed the short-term spatial memory deficits of APP23/PS45 transgenic mice.

Next, the Morris water maze was used to examine the effects of ALA on learning and memory in APP23/PS45 mice. In the visible platform test, no significant differences in escape latency and path length were observed between the AD + Veh and AD + ALA groups. The escape latency times for the WT + Veh, AD + Veh and AD + ALA mice were 49.56 ± 2.34 s, 51.76 ± 2.16 s, and 51.29 ± 2.67 s, respectively, while the path lengths were 8.04 ± 0.68 m, 6.72 ± 0.58 m, and 8.10 ± 0.55 m, respectively (Fig. 1G, H). These results indicated there were no differences in motor ability or vision among the three groups. The hidden platform period from day 2 to 5 is an acquired training test that evaluates the learning ability of mice. Mice in the AD + Veh group showed poor learning ability, with an escape latency time of 51.13 ± 2.90 s, 50.07 ± 4.06 s, 47.79 ± 3.07 s and 47.17 ± 3.36 s on day 2, 3, 4 and 5, respectively (Fig. 1I). However, the escape latency of WT + Veh mice decreased progressively from 40.23 ± 3.31 s on day 2 to 26.70 ± 3.32 s on day 5 (WT + Veh vs. AD + Veh, *P* < 0.001; Fig. 1I). The escape latency of AD + ALA mice also decreased from 36.87 ± 3.09 s on day 2 to 27.42 ± 5.04 s on day 5 (AD + ALA vs. AD + Veh; *P* < 0.001; Fig. 1I). The heat maps for mice movement tracks on day 2 and 5 are shown in Fig. 1J. The above results indicated that ALA administration significantly improved the learning deficits of APP23/PS45 mice. The last day is a probe trial to evaluate the spatial memory of mice. No significant differences in platform passing times were observed among the three groups (Fig. 1K). As shown in Fig. 1L, the number of entries into the SW3 platform quadrant for WT + Veh, AD + Veh and AD + ALA mice was 5.50 ± 0.43, 3.70 ± 0.63, and 7.40 ± 1.10 times, respectively. Thus, AD + Veh mice had less entries into the platform quadrant compared with WT + Veh mice (*P* < 0.05), while AD + ALA mice had more entries than AD + Veh mice (*P* < 0.01). In summary, the Morris water maze revealed that APP/PS45 transgenic mice had reduced learning and memory abilities compared to their WT littermates. However, ALA reversed the cognitive deficits in these transgenic mice.

### ALA ameliorated amyloid pathologies in APP23/PS45 transgenic mice

**Fig. 2:**
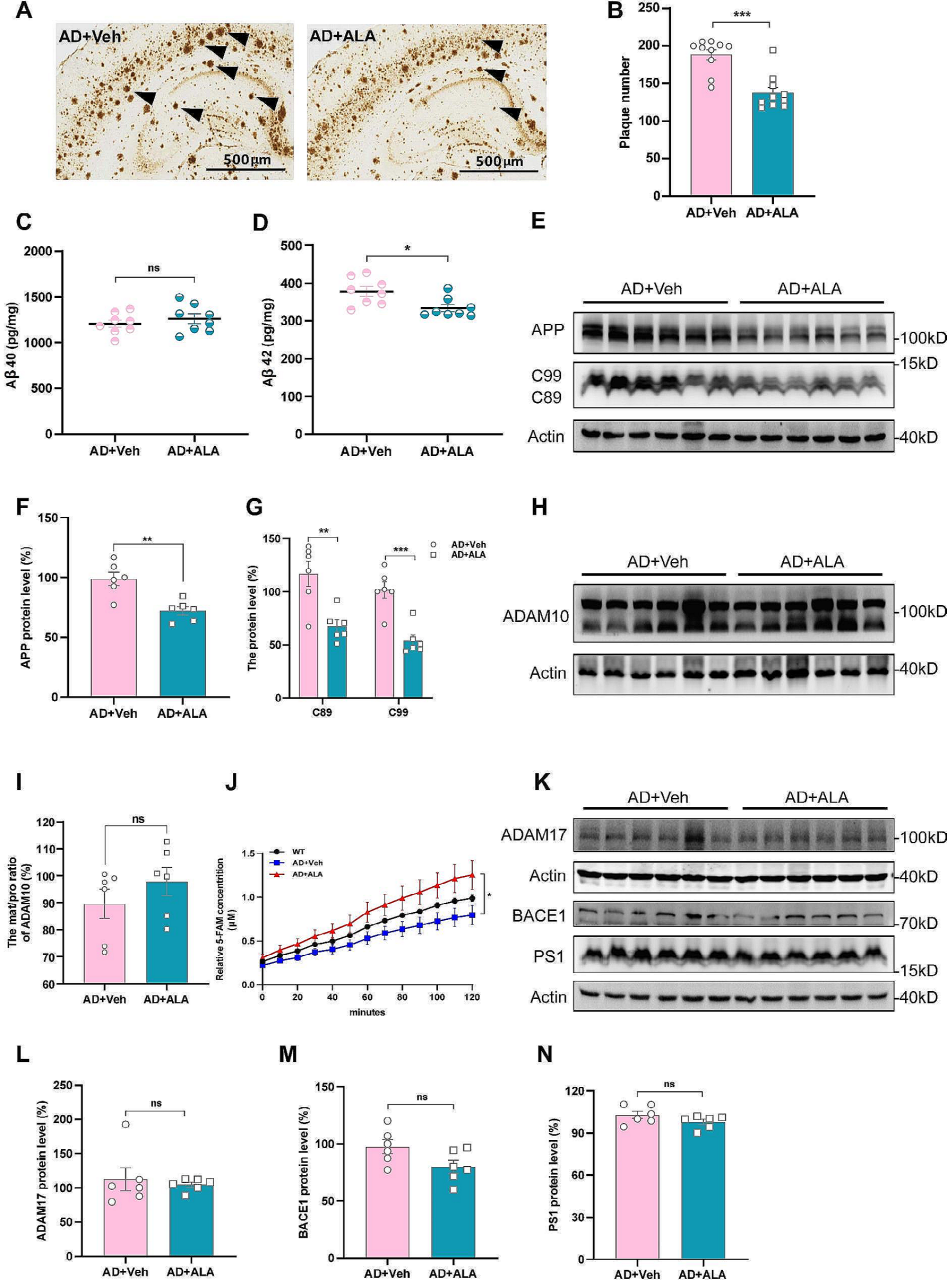
The amyloid pathologies of brain tissue in APP23/PS45 transgenic mice were ameliorated by ALA. **(A)** Representative images of senile plaques detected by 4G8 immunostaining from AD + Veh and AD + ALA mice. Black arrows indicate plaques. **(B)** Mean numbers of senile plaques, *n* = 10. ***P < 0.001. **(C, D)** ELISA was performed to measure the levels of Aβ40 and Aβ42 in the brain tissue of AD + Veh and AD + ALA mice. *n* = 8, *P < 0.05. **(E-G)** Immunoblot bands and protein levels of APP, C99 and C89. *n* = 6. **P < 0.01, ***P < 0.001. **(H, I)** Western blots analysis of ADAM10. **(J)** The α-cleavage activity of ADAM10 was increased in AD + ALA compared with AD + Veh mice. *n* = 3. *P < 0.05. **(K-N)** Immunoblot bands and protein levels of ADAM17, BACE1 and PS1. *n* = 6

We next examined the effects of ALA on the amyloid pathology of brain tissue from APP23/PS45 mice. Senile plaques in brain tissue were evaluated by immunohistochemistry between AD + Veh and AD + ALA group mice. The size of these plaques was significantly smaller in the AD + ALA group compared to the AD + Veh group (Fig. 2A). Quantification also revealed there were significantly fewer plaques in the ALA treated group (AD + ALA, 137.43 ± 7.39 vs. AD + Veh, 188.20 ± 6.89; *P* < 0.001; Fig. 2B). Next, ELISA was performed to evaluate the levels of Aβ40 and Aβ42. ALA treatment reduced the level of Aβ42 in APP23/PS45 mice (AD + ALA, 333.82 ± 8.97 pg/mg vs. AD + Veh, 377.86 ± 12.55 pg/mg; *P* < 0.05; Fig. 2D). However, no significant change was observed in the level of Aβ40 (AD + ALA, 1261.80 ± 52.79 pg/mg vs. AD + Veh, 1207.36 ± 40.50 pg/mg; *P* > 0.05; Fig. 2C). The observed effects of ALA on senile plaques and Aβ42 suggested that its impact on cognitive performance may be due to its inhibition of amyloidogenesis in the brain.

Western blot analysis of brain tissue was performed to investigate the effects of ALA on APP metabolism in vivo. ALA significantly decreased the protein levels of APP, C89 and C99 in the brain of APP23/PS45 mice (Fig. 2E-G). Next, we explored the specific mechanism by which ALA alters APP processing in the brain of AD mice. Western blot was used to compare the expression of APP-cleaving enzymes (α, β and γ secretases) between the AD + Veh and AD + ALA groups. The mature/pro-forma (mat/pro) ratio for ADAM10 was slightly increased in the brain tissues of AD + ALA, although this did not reach significance (AD + ALA: 97.79% ± 5.20% vs. AD + Veh: 89.58% ± 5.39%; *P* > 0.05; Fig. 2H, I). Surprisingly, ALA treatment significantly increased the α-cleavage activity of ADAM10 (Fig. 2J) without altering the expression levels of ADAM17, BACE1 and PS1 did not change (Fig. 2K-N). These results indicated that ALA reduced the amyloid metabolic pathway of APP.

### ALA promoted α-secretase cleavage of APP in vitro

**Fig. 3:**
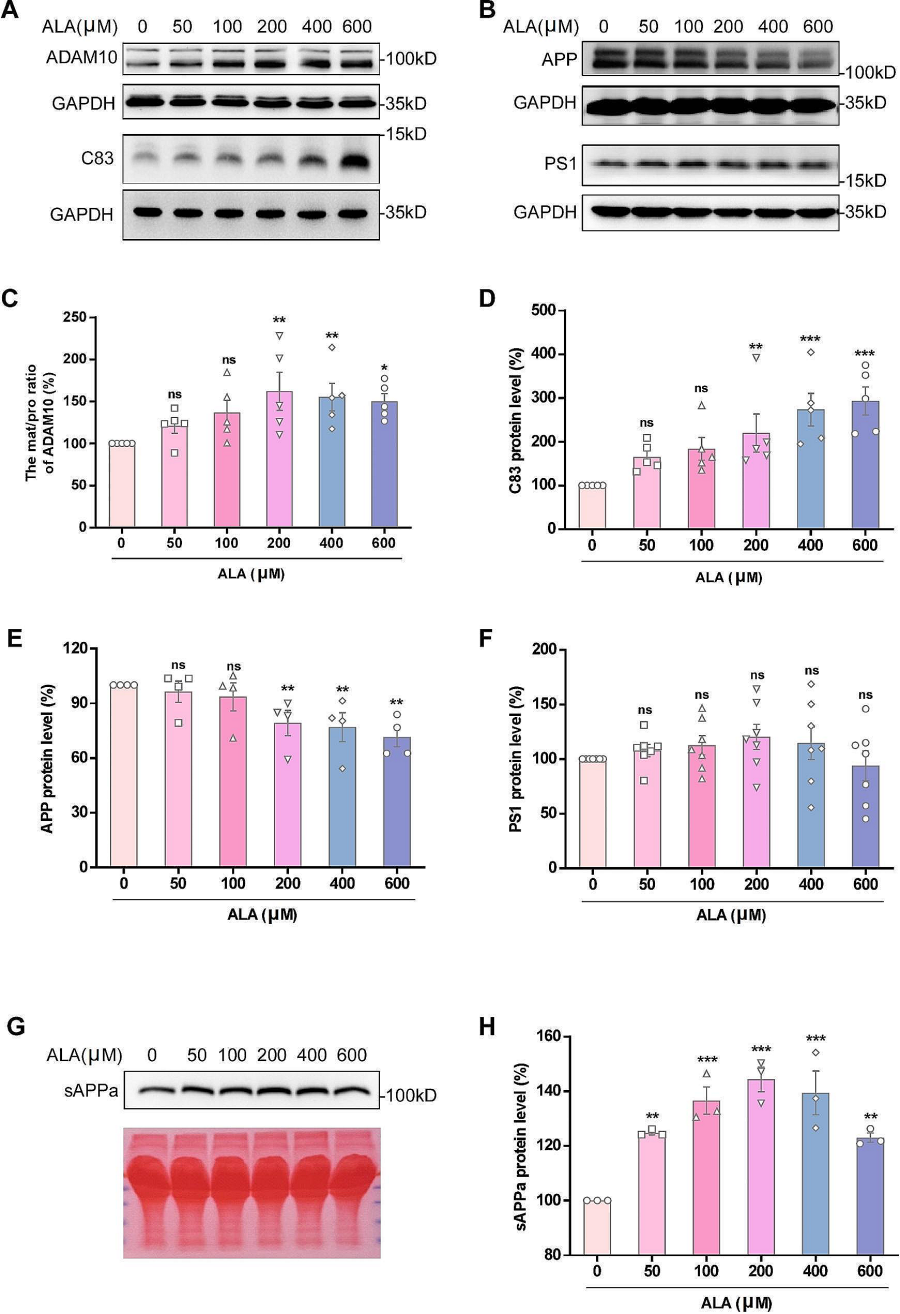
The α-secretase cleavage of APP in 20E2 cells was increased by ALA. 20E2 cells was treated with ALA (0, 50, 100, 200, 400 or 600 µM) for 24 h, and then total cell lysates and culture media were subjected to immunoblotting. **(A-F)** Western blots and corresponding quantification were performed for ADAM10, C83, APP and PS1 in 20E2 cell lysates. **(G-H)** The sAPPα from media was detected with 6E10 primary antibody, and total protein was stained with Ponceau S as an internal reference. Relative levels of sAPPα in media. *n* = 3–7. *P < 0.05, **P < 0.01, ***P < 0.001

We further investigated the specific mechanism by which ALA attenuates the amyloid pathway of APP in a mouse model of AD by conducting in vitro experiments with the 20E2 cell line that stably overexpressed APP. These cells were treated with increasing concentrations of ALA (0, 50, 100, 200, 400 or 600 µM) for 24 h, and the level of APP catabolites and sequential cleavage enzymes was then evaluated. ALA treatment significantly increased the mat/pro ratio for ADAM10 (Fig. 3A, C). The level of C83 also increased sharply in a concentration-dependent manner (Fig. 3A, D). Furthermore, the expression of APP gradually decreased (Fig. 3B, E). Consistent with the results of in vitro experiments, ALA did not affect the expression of PS1 in 20E2 cells (Fig. 3B, F). Western blot analysis of the culture media from 20E2 cells treated with ALA for 24 h showed an increased level of sAPPα (Fig. 3G, H). These results indicated that ALA promoted the non-amyloidogenic processing of APP in vitro.

### ADAM10 promoted α-secretase cleavage of APP in 20E2 cells treated by ALA

**Fig. 4:**
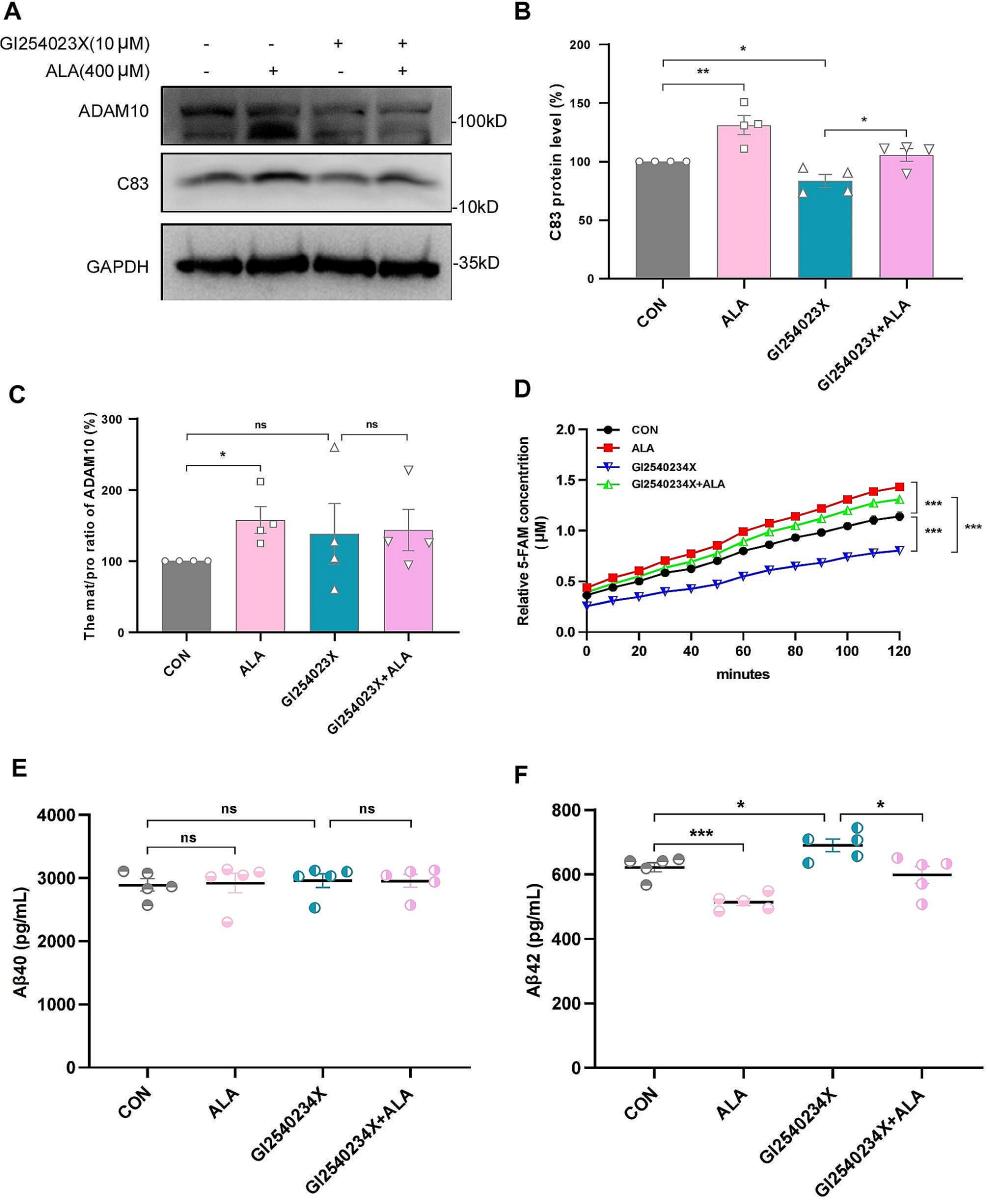
The crucial role of ADAM10 in promoting α-secretase cleavage of APP following ALA treatment of 20E2 cells. 20E2 cells were treated with ALA (400 µM) or GI254023 × (10 µM) for 24 h. **(A-C)** Representative Western blot bands and protein levels of C83 and ADAM10. *n* = 4. *P < 0.05, **P < 0.01. **(D)** Evaluation of ADAM10 protease activity in 20E2 cell lysates, ***P < 0.001. **(E, F)** The levels of Aβ40 and Aβ42 in the culture media of 20E2 cells were measured by ELISA. *n* = 5. *P < 0.05, ***P < 0.001

ADAM10 is involved in the non-amyloidogenic processing of APP. It cleaves APP at the α-site to produce the intermediate metabolite C83, thereby reducing the generation of intact Aβ. To further evaluate the effect of ALA on ADAM10, 20E2 cells were treated with either ALA (400 µM) or GI254023 × (10 µM) to investigate changes in the APP non-amyloid metabolic pathway. As expected, GI254023X significantly reduced C83 compared to the CON group (GI254023X, 83.42% ± 5.42% vs. CON, 100%; *P* < 0.05; Fig. 4A, B), whereas ALA restored C83 after GI254023X treatment (GI254023X + ALA, 105.63% ± 5.40% vs. GI254023X, 83.42% ± 5.42%; *P* < 0.05; Fig. 4A, B). Both ALA and GI254023X increased the mat/pro ratio for ADAM10 compared with the CON group, but this was only significant with ALA (Fig. 4C). A kinetic assay for ADAM10 activity was also conducted. GI254023X was found to strongly inhibit ADAM10 activity, whereas ALA reversed the inhibition of ADAM10 α-secretase cleavage activity caused by GI254023X (Fig. 4D). In addition, the levels of Aβ40 and Aβ42 were measured in the culture media of 20E2 cells treated with ALA or GI254023X. No significant differences in the Aβ40 level were observed between these groups (Fig. 4E). However, ALA reduced the Aβ42 level (ALA, 513.88 ± 11.19 pg/mL vs. CON, 622.43 ± 14.65 pg/mL; *P* < 0.05; Fig. 4F), whereas GI254023X increased the Aβ42 level compared with the CON group (GI254023X, 690.15 ± 19.65 pg/mL vs. CON, 622.43 ± 14.65 pg/mL; *P* < 0.001; Fig. 4F). The level of Aβ42 in media from 20E2 cells treated with GI254023X + ALA was significantly lower than in the GI254023X group (GI254023X + ALA, 598.37 ± 26.44 pg/mL vs. GI254023X, 690.15 ± 19.65 pg/mL; *P* < 0.05; Fig. 4F). These findings confirmed that ALA promoted the non-amyloidogenic processing of APP by increasing the α-secretase activity of ADAM10.

### ALA increased autophagic flux in vitro

**Fig. 5:**
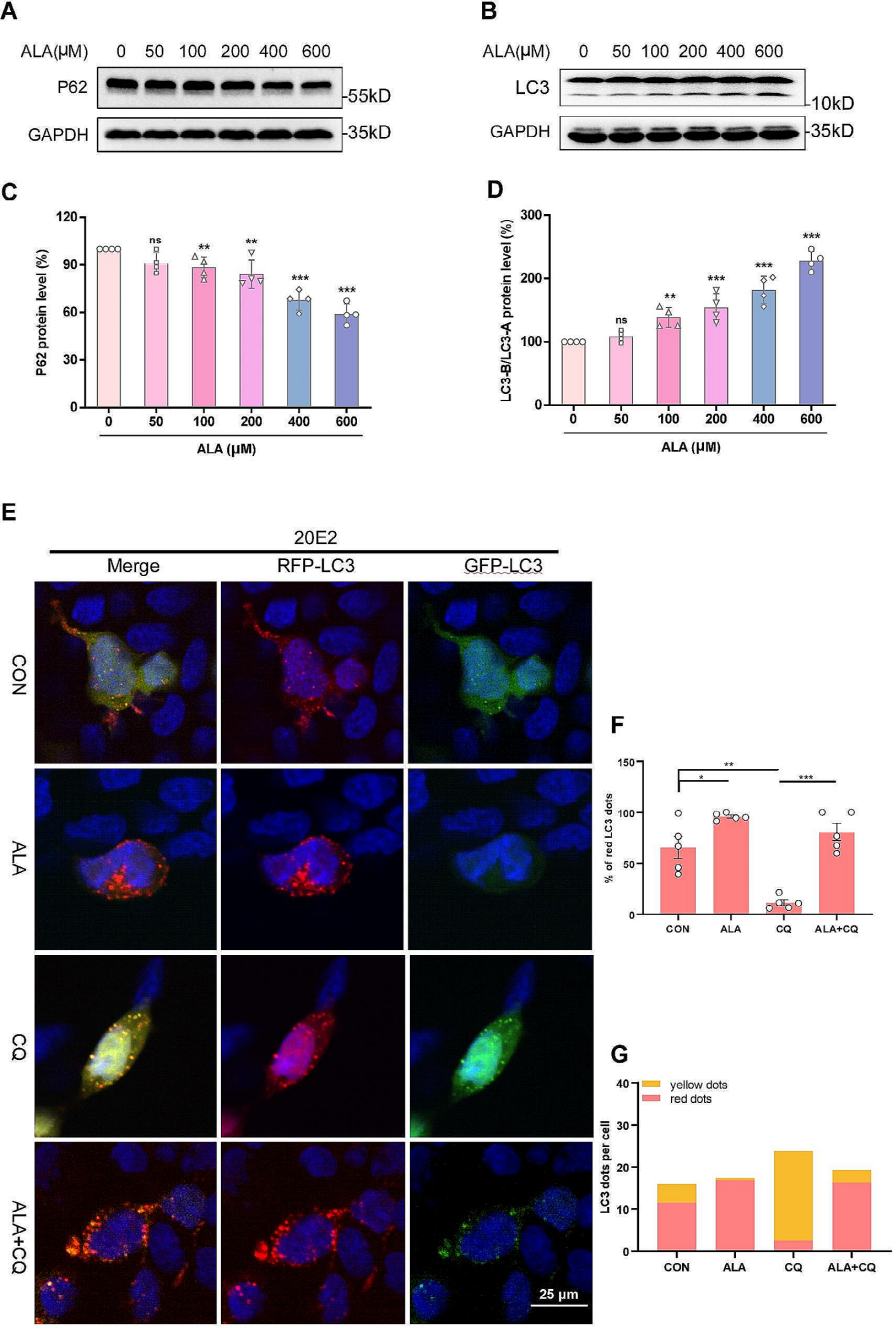
Autophagy was activated in 20E2 cells after ALA treatment. **(A-D)** 20E2 cells were treated with ALA (0, 50, 100, 200, 400 or 600 µM) for 24 h. Representative Western blot bands are shown for P62 and LC3. Protein levels for P62 and bar plot summary of LC3-B/LC3-A. *n* = 4. **P < 0.01, ***P < 0.001. **(E)** 20E2 cells were transfected with mRFP-eGFP-LC3 plasmid for 24 h, then treated with ALA (400 µM) or CQ (25 µM) for 24 h. Representative images of CON, ALA, CQ and ALA + CQ groups cells were showed. **(F)** The proportion of red LC3 dots to total LC3 dots (sum of red and yellow LC3 dots) among four groups. **(G)** The number of yellow and red LC3 dots per cell was quantified. At least 30 cells were counted in each group. *P < 0.05, **P < 0.01, ***P < 0.001

To further explore the mechanism by which ALA affects ADAM10, we evaluated the autophagic level of 20E2 cells treated with ALA (0, 50, 100, 200, 400 or 600 µM) for 24 h. Western blot analysis revealed that the P62 protein level decreased and the LC3-B/LC3-A ratio increased with higher concentrations of ALA (Fig. 5A-D). To evaluate the complete autophagic flux, 20E2 cells transfected with mRFP-eGFP-LC3 plasmid were treated with ALA (400 µM) or CQ (25 µM) for 24 h and the LC3 dots subsequently quantified by fluorescence microscopy. This analysis revealed 65.37 ± 10.82% red dots in the CON group, indicating a smooth autophagic flux as expected. However, the proportion of red dots in the ALA group was increased compared with the CON group (ALA, 95.82% ± 1.52% vs. CON, 65.37% ± 10.82%; *P* < 0.05; Fig. 5E, F). In contrast, the proportion of red dots decreased in CQ-treated cells (CQ, 10.97% ± 2.80% vs. CON, 65.37% ± 10.82%; *P* < 0.01; Fig. 5E, F) and the number of yellow dots was increased (CQ, 21.24 ± 2.94 vs. CON, 4.47 ± 2.08; *P* < 0.01; Fig. 5E, G), indicating that CQ diminished autophagic flux. The proportion of red dots in ALA + CQ group was significantly increased (ALA + CQ, 80.72% ± 8.31% vs. CQ, 10.97% ± 2.80%; *P* < 0.001 Fig. 5E, F), and the number of yellow dots decreased compared with the CQ group (ALA + CQ, 2.93 ± 1.29 vs. CQ, 21.24 ± 2.94; *P* < 0.01; Fig. 5E, G). In summary, these results indicated that ALA can activate autophagy in 20E2 cells.

### ALA improved mitophagy in vitro

**Fig. 6:**
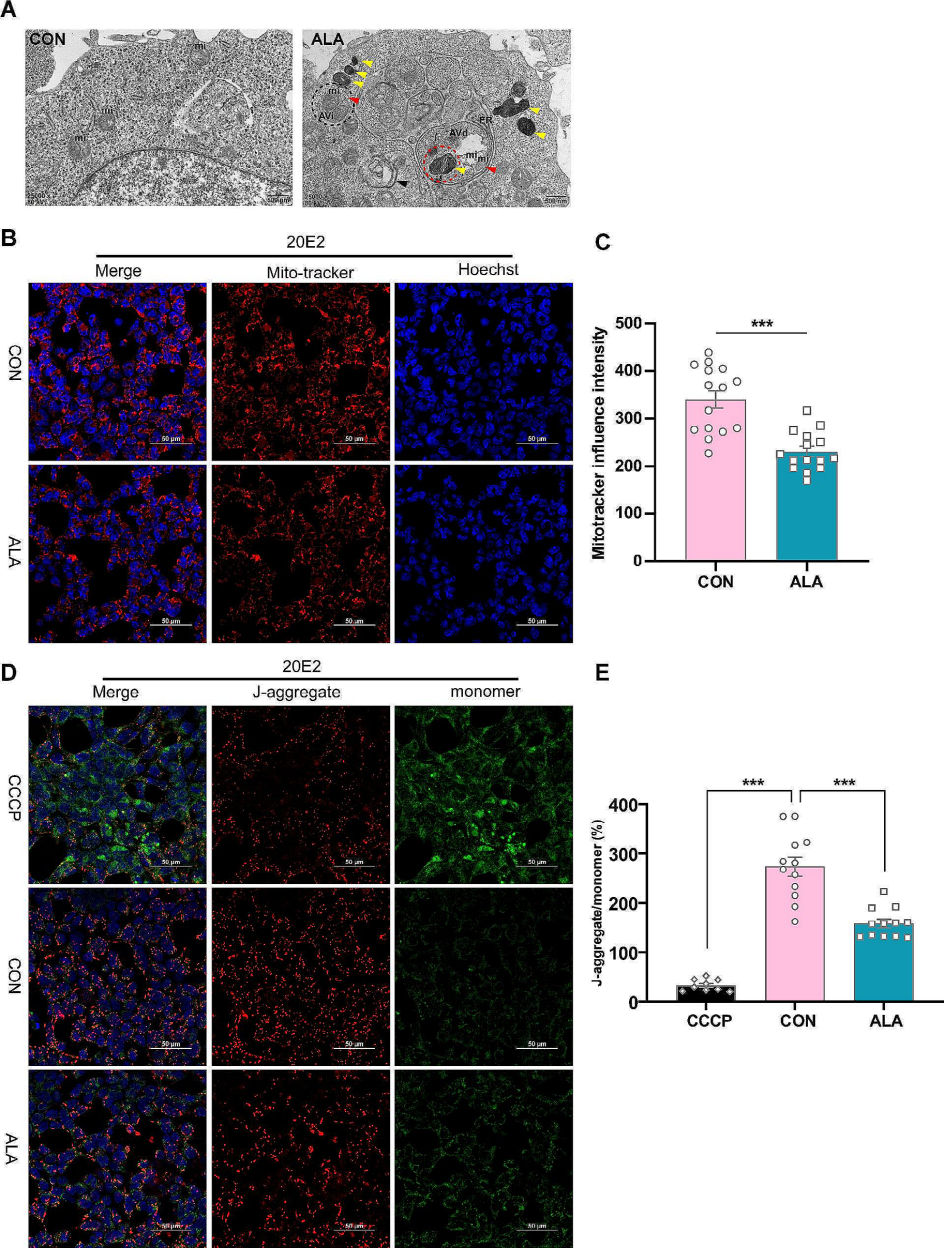
The mitophagy in 20E2 cells was improved by ALA treatment. **(A)** TEM images of 20E2 cells in CON and ALA (400 µM) groups. Autophagosomes (red arrows) and autolysosomes (yellow arrows) were observed in the ALA group. In the ALA-treated 20E2 cell, two mitochondria are seen wrapped within the bilayer limiting membrane of an autophagosome. These structures were early AVi (black dashed circle). AVd contained partially degraded mitochondria and endosomal/lysosomal particles. The yellow arrows indicate mitochondrial vesicles in which partially degraded mitochondria (red dashed circles) were also observed. mi, mitochondrion. **(B, C)** Mitochondrial mass was detected using the Mito-Tracker fluorescent probe. **(D, E)** MMP was performed with the JC-1 dual fluorescence probe. ***P < 0.001

TEM was used to observe the autophagy of 20E2 cells treated with ALA. Minimal autophagy was observed in the control group, whereas the number of autophagosomes (red arrows) and autolysosomes (yellow arrows) increased significantly in the ALA group (400 µM). This result confirmed that ALA indeed promotes autophagy in 20E2 cells (Fig. 6A).

Surprisingly, two intact mitochondria were found in initial autophagic vacuoles (AVi). These mitochondria are marked by black dashed circles in 20E2 cells treated with ALA. Mitochondrial components were also found in degradative autophagic vacuoles (AVd), together with mitochondrial vesicles (yellow arrows) and partially degraded mitochondria (red dashed circles) (Fig. 6A). We therefore speculated that ALA might regulate non-amyloidogenic processing of APP by promoting mitophagy. The Mito-Tracker Red CMXRos Kit was used to evaluate mitochondrial mass. Treatment of 20E2 cells with ALA (400 µM) reduced the red fluorescence intensity of Mito-Tracker (Fig. 6B, C), indicating that ALA reduced the number of mitochondria. Furthermore, ALA appeared to decrease the MMP of 20E2 cells, as shown by the reduced ratio of J-aggregate/monomer (Fig. 6D, E). Based on the increased mitochondrial autophagy observed by TEM, and the decrease in mitochondrial number and MMP in 20E2 cells, we concluded that ALA can improve mitophagy in vitro.

### BNIP3L-mediated mitophagy promoted ADAM10 α-secretase cleavage of APP

**Fig. 7:**
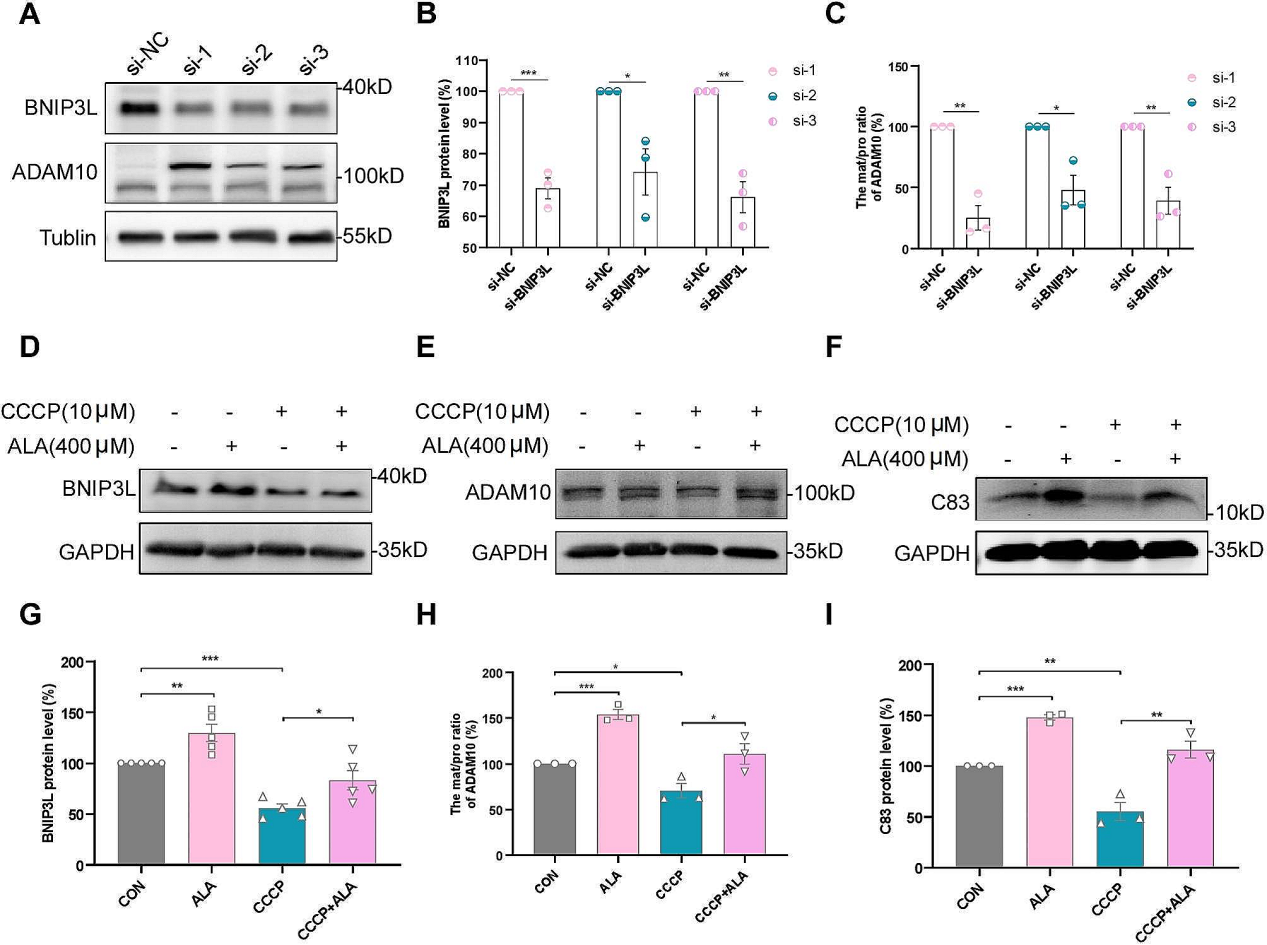
BNIP3L-mediated mitophagy is involved in the regulation of ADAM10. **(A-C)** Three different siRNAs were used to knockdown BNIP3L in 20E2 cells. Representative Western blot bands and protein levels for BNIP3L and ADAM10 were shown. **(D-I)** Representative Western blot bands and protein levels for BNIP3L, ADAM10 and C83 in 20E2 cells treated with ALA (400 µM) or CCCP (10 µM). *n* = 3–5. *P < 0.05, **P < 0.01, ***P < 0.001

Knockdown of BNIP3L by siRNA was conducted in 20E2 cells to assess the effect on expression of ADAM10. Western blot analysis verified the successful inhibition of BNIP3L by siRNA (Fig. 7A, B). The mat/pro ratio for ADAM10 was significantly reduced following BNIP3L knockdown (si-1, 25.11% ± 10.07% vs. si-NC, 100%, *P* < 0.01; si-2, 47.89% ± 12.23% vs. si-NC, 100%, *P* < 0.05; si-3, 39.13% ± 11.09% vs. si-NC, 100%, *P* < 0.01; Fig. 7A, C). These results indicated that BNIP3L was involved in the regulation of ADAM10.

To confirm the important role of mitophagy in the ALA-mediated increase in APP α-cleavage, 20E2 cells were treated with CCCP (10 µM) or ALA (400 µM). Western blot analysis was used to detect the expression of BNIP3L, a mitophagy receptor protein. ALA increased the expression of BNIP3L in 20E2 cells (ALA, 129.59% ± 8.55% vs. CON, 100%; *P* < 0.01; Fig. 7D, G), whereas CCCP strongly decreased BNIP3L expression (CCCP, 55.66% ± 4.07% vs. CON, 100%, *P* < 0.001; Fig. 7D, G). The level of BNIP3L in the CCCP + ALA group was significantly higher than in the CCCP group (CCCP + ALA, 83.12% ± 9.46% vs. CCCP, 55.66% ± 4.07%, *P* < 0.05; Fig. 7D, G). These results further indicated that ALA enhanced mitophagy, while attenuating the CCCP-induced impairment of mitophagy in 20E2 cells. CCCP was also found to decrease the mat/pro ratio for ADAM10 in 20E2 cells (CCCP, 70.44% ± 7.93% vs. CON, 100%; *P* < 0.05; Fig. 7E, H), whereas ALA partially restored the decreased ratio (CCCP + ALA, 110.78% ± 11.18% vs. CCCP, 70.44% ± 7.93%, *P* < 0.05; Fig. 7E, H). The trend observed for C83 was consistent with that of ADAM10 (ALA, 147.79% ± 2.71% vs. CON, 100%, *P* < 0.001; CCCP, 55.03% ± 8.85% vs. CON, 100%, *P* < 0.01; CCCP + ALA, 115.96% ± 8.22% vs. CCCP, 55.03% ± 8.85%, *P* < 0.01; Fig. 7F, I). These results further confirmed that BNIP3L-mediated mitophagy promoted the cleavage of APP by ADAM10 α-secretase.

## Discussion

Our animal study found that ALA treatment improved cognitive dysfunction in APP23/PS45 transgenic mice (Fig. 1E-L). Studies have revealed that anxiety-like behaviour may have specific effects on learning and memory [59–61]. In the present study, mice in the WT, AD-Veh and AD-ALA groups exhibited no obvious anxiety-like behaviours (Fig. 1A-D). Therefore, we believed that ALA was responsible for the positive impact on the cognitive performance of AD transgenic mice. APP is endoproteolytically processed by BACE1 and γ-secretase to release amyloid peptides (Aβ40 and 42), which aggregate to form amyloid plaques in the brains of AD. Previous studies in transgenic mice and cultured cell models have demonstrated that the familial AD-PS1 (FAD-PS1) variants shift the ratio of Aβ40 : 42 to favor Aβ42 [62–64]. In transgenic mice that co-express the Swedish mutation of APP and two FAD-PS1 variants, the researchers further showed that the shift in Aβ42 : 40 ratios associated with the expression of FAD-PS1 variants is due to a specific elevation in the steady-state levels of Aβ42, while maintaining a constant level of Aβ40 [65]. Aggregates of Aβ42 initiate a neurotoxic cascade and are thought to be critical to the formation of amyloid plaques that ultimately cause learning and memory to decline [66, 67]. In our study, ALA significantly reduced amyloid plaques in APP23/PS45 mice. ALA significantly reduced the level of Aβ42 in the brains of APP23/PS45 mice, while the changes of Aβ40 was not significant (Fig. 2A-D). Thus, ALA reduced amyloidosis in the brains of APP23/PS45 transgenic mice.

What is the mechanism by which ALA attenuates amyloidosis in the brain tissue of APP23/PS45 transgenic mice? Western blot analysis of brain tissues from the AD-Veh and AD-ALA groups showed that ALA treatment in vivo decreased the levels of APP and of the β-secretase cleavage products C99 and C89, but not the expression of β- and γ-secretases (Fig. 2E, G, K-N). Meanwhile, we found that the mat/pro ratio for ADAM10 was slightly increased in AD-ALA mice. Thus, we evaluated its protease activity and surprisingly found that it was also increased (Fig. 2H-J). However, the mat/pro ratio for ADAM10 in the brain tissue was not significantly different between AD-Veh and AD-ALA mice. There may be several reasons for this. Firstly, APP23/PS45 transgenic mice use the Thy1.2 promoter in the APP expression vector. The Thy1.2 promoter mainly targets neurons, meaning that APP is specifically expressed in these cells. The mammalian brain is mainly composed of neurons and glial cells [68, 69], and the constitutive cleavage of APP by α- and β-secretase occurs primarily in neurons [70, 71]. However, the protein samples evaluated in the present study were derived from the total brain tissue. Secondly, the regulation of protein expression in vivo is a complex process involving both positive and negative feedback. It was therefore reasonable to postulate that ADAM10 is effective at reducing amyloid plaques. It has been reported that ADAM10 partially competes with γ-secretase to cleave the C99 and C89 fragments produced by β-secretase [72, 73]. In the present study, the levels of C99 and C89 were also decreased in the brain tissue of mice treated with ALA. This may be explained by a decrease in their production, or by an increase in their cleavage due to ADAM10. In summary, we hypothesized that the improvement of learning and memory deficits and reduction of amyloidosis in APP23/PS45 mice may be related to the regulation of ADAM10 protein expression and enzyme activity by ALA.

Increasing the α-secretase cleavage of APP by ADAM10, one of the most important constitutive proteases in neurons, has attracted the attention of researchers as a possible strategy to reduce neurotoxic Aβ peptides [72, 74, 75]. Animal study suggested that ADAM10 may be involved in the regulation of APP metabolism by ALA. To further explore the mechanism of action of ALA, we also performed in vitro studies using 20E2 cells with stable overexpression of APP. The expression of ADAM10 mat/pro and C83 by these cells was observed to increase as the concentration of ALA was increased (Fig. 3A, C, D). APP expression decreased with increasing ALA, whereas the expression of PS1 did not change significantly (Fig. 3B, E, F). The ADAM family mediates ectodomain shedding of cell surface molecules, such as TNF-α, IL6R, and the transmembrane chemokines CX3CL1 and CXCL16. It can do this through both constitutive shedding and phorbol myristate acetate (PMA)-induced shedding [76–78]. ADAM10 is a major member of the ADAM family and induces α-secretase cleavage of APP through constitutive shedding [72, 79]. Recently we reported that ADAM10 is essential for CNTNAP2 processing and its function; the inhibition of the ADAM10-mediated α-secretase cleavage by pathogenic mutations underlies autism spectrum disorders’ pathogenesis [80].The S1’ binding pocket of ADAM10 is an important determinant of the substrate selectivity of this protease [81]. The P1’ phenylpropyl substituent of GI254023X has an improved fit that allow it to bind within the S1’ pocket, thereby inhibiting constitutive cytokine shedding events [76]. The shedding of CX3CL1 and CXCL16 was profoundly reduced in ADAM10-deficient murine embryonic fibroblasts, confirming that constitutive shedding of these cytokines is primarily mediated by ADAM10 [76]. Furthermore, GI254023X did not affect PMA-induced shedding, and is therefore thought to be a specific inhibitor of ADAM10 [82]. Thus, we also confirmed the key role of ADAM10 in modulating APP metabolism by using the specific ADAM10 inhibitor GI254023X. The mat/pro ratio for ADAM10 was still highly elevated after GI254023X treatment, with no difference between the ALA and GI254023X + ALA groups (Fig. 4A, C). We speculated that GI254023X did not inhibit ADAM10 by reducing its protein level, but rather by specifically binding to it through its peptide or pseudopeptide structural functional group. ALA may promote the dynamic balance of ADAM10 expression, and inhibited ADAM10 could be degraded at an accelerated rate and replaced with new and active ADAM10. Therefore, the ADAM10 level may have been saturated in the ALA, GI254023X and GI254023X + ALA groups of 20E2 cells, and hence no significant difference in protein level was observed. If the P1’ phenylpropyl substituent of GI254023X occupies the S1’ binding pocket of ADAM10, ALA might then induce the production of new mature ADAM10, which would partially compensate for the reduced ADAM10 activity caused by GI254023X. This could explain why C83 and ADAM10 activity were partially restored in the GI254023X + ALA group (Fig. 4A, B, D). Other authors have argued that α- and β-secretases compete for the APP substrate, so that increased α-secretase cleavage causes a decrease in β-secretase cleavage, and vice versa [83, 84]. In addition, mutations in APP identified from FAD kindreds alter the protein’s normal processing, causing either increased production of both peptides or a specific elevation in Aβ42 [85–88]. In this regard, our results showed that inhibition of ADAM10 by GI254023X led to an increased level of Aβ42 in the media, confirming that GI254023X indirectly enhanced β-secretase cleavage (Fig. 4F). In addition, the component of α-secretase directly promoted by ALA competes for APP with β-secretase that is indirectly promoted by GI254023X, resulting in partial reduction of Aβ42 (Fig. 4F). These results supported the notion that potentiation of α-secretase, especially by ADAM10, may be an effective therapeutic approach for AD [89, 90].

We demonstrated here that ALA causes increased expression and activity of ADAM10, but the specific mechanism behind this remains unclear. ADAM10 is a long-lived protein, and its proteolytic activity for α-cleavage is related to the mature enzyme [49, 91, 92]. A recent study showed that overexpression of the transcription factor EB induces the mature form of ADAM10 by activating the entire autophagy-lysosome pathway [93]. Another study reported that ADAM10 is degraded by the lysosomal pathway via asparagine endopeptidase [94]. Autophagy is markedly impaired in AD, and this also affects the accumulation of Aβ peptides and p-tau protein [95–97]. Using a mouse model of AD, Lee and colleagues suggested that faulty autolysosome acidification induces the autophagic accumulation of Aβ in neurons and the formation of senile plaques in brain tissues [98]. Although there is substantial evidence suggesting that dysregulation of autophagy contributes to the neuropathology of AD, so far there have been few studies on the regulatory mechanisms of autophagy in the APP metabolic pathway. In the current study, ALA was found to activate autophagic flux in an AD cell model (Fig. 5A-G). Moreover, the damaged mitochondria were observed by TEM to be engulfed by autophagosomes and then degraded in autolysosomes (Fig. 6A). Mitochondrial dysfunction with aging leads to reduced oxidative phosphorylation [99]. The resulting decrease in ATP level triggers energy stress, which could accelerate Aβ and tau pathology [100]. Mitochondrial dysfunction in the brain of a mouse model of AD was found to occur prior to Aβ deposition [101]. The accumulation of damaged neuronal mitochondria has been observed in both sporadic and familial AD, as well as in animal models. Mitophagy dysfunction is therefore a fundamental pathological hallmark of AD [102–104].

Mitophagy is a subtype of selective autophagy that removes defective and superfluous mitochondria [105, 106]. The specific receptor BNIP3L is involved in receptor-mediated mitophagy and binds directly to the LIR motif of LC3 to mediate the elimination of mitochondria [107, 108]. BNIP3L directly targets mitochondria and facilitates the reduction of MMP, leading to the recognition of dysfunctional mitochondria by autophagosomes, thereby inducing mitophagy and enhancing mitochondrial function [109]. Studies have shown that BNIP3L-mediated mitophagy promotes the autophagy-lysosome degradation of mitochondria in reticulocytes [110]. CCCP is a mitochondria toxic drug that uncouples the electron transport chain and phosphorylation, leading to major disruption of the MMP and resulting in mitochondrial damage [111–113]. Mitochondrial depolarization selectively induces mitophagy, thereby preventing the accumulation of damaged mitochondria [110, 114]. In the present study, CCCP significantly decreased MMP and BNIP3L expression, whereas ALA moderately decreased MMP and increased BNIP3L expression (Figs. 6D, E; 7D, G). ALA also reduced the mitochondrial mass, as determined by Mito-Tracker (Fig. 6B, C). Combined with the observation of TEM, we concluded that ALA activated mitophagy, whereas CCCP inhibited mitophagy. Activation of mitophagy has been shown to improve neuronal mitochondrial function [115, 116]. Additionally, ALA is an essential coenzyme in mitochondria. Supplementation with ALA can ameliorate mitochondrial dysfunction and alleviate cognitive deficits in AD-like animal models and in patients with mild or moderate dementia [117–120]. In the current study, ALA treatment reduced pathological APP processing and improved learning and memory ability in APP23/PS45 transgenic mice (Figs. 1, 2 and 3). Therefore, we speculated that ALA may increase the expression and activity of ADAM10 by inducing BNIP3L-mediated mitophagy, thereby promoting the non-amyloid cleavage pathway of APP. Studies have shown that BNIP3L overexpression can reduce damage to mitochondria caused by CCCP treatment [107]. In the present study, knockdown of BNIP3L expression in 20E2 cells with siRNA resulted in a significant reduction in the mat/pro ratio for ADAM10, thus demonstrating that BNIP3L can indeed regulate ADAM10 (Fig. 7A-C). ALA partially compensated for the loss of BNIP3L induced by CCCP, indicating that ALA may restore mitophagy dysfunction by inducing BNIP3L (Fig. 7D, G). CCCP decreased the expression of ADAM10, which could be partially rescued by ALA (Fig. 7E, H). The level of C83 was consistent with the expression of ADAM10 (Fig. 7F, I). That supports the notion that BNIP3L-mediated mitophagy is involved in the regulation of ADAM10.

Following on from the observation that ALA attenuated cognitive deficits and amyloidosis in APP23/PS45 transgenic mice, further analysis in 20E2 cells showed that ALA mediated an increase in the cleavage of APP by ADAM10 α-secretase. This occurred through mitophagy and in a BNIP3L-dependent manner. However, because we did not perform knockout experiments for BNIP3L in APP23/PS45 mice, there is insufficient evidence to confirm that BNIP3L-mediated mitophagy directly attenuates cognitive deficits and amyloidosis, with further in vivo studies required. Additional experiments involving treatment of APP23/PS45 transgenic mice with CCCP or ALA are also required to confirm the results of our in vitro study. Cell-based assays with 20E2 cells showed that knockdown of BNIP3L decreased the level of mature ADAM10. However, the specific molecular mechanism involved requires further investigation with additional cell-based assays. Meanwhile, other neurogenic cell models should be used to validate the findings.

## Conclusion

The evidence presented in this study indicated that ALA can increase the expression and α-secretase activity of ADAM10 by activating BNIP3L-mediated mitophagy. This has the effect of moving the processing of APP towards a non-pathological pathway, and alleviating cognitive impairments in APP23/PS45 transgenic mice. The current findings highlighted a potential therapeutic role for ALA in the treatment of AD by enhancing non-amyloidogenic processing of APP.

### Electronic supplementary material

Below is the link to the electronic supplementary material.

Supplementary Material 1

Supplementary Material 2

Supplementary Material 3

Supplementary Material 4

Supplementary Material 5

## Data Availability

No datasets were generated or analysed during the current study.

## Abbreviations

AD: Alzheimer’s disease
ADAM10: A Disintegrin and Metalloproteinase 10
ALA: Alpha-lipoic acid
APP: Amyloid-β precursor protein
AVd: Degradative autophagic vacuoles
AVi: Initial autophagic vacuoles
Aβ: Amyloid-β protein
BACE1: Beta-site amyloid β precursor protein cleaving enzyme
C83: 83 amino acid C-terminal fragment
CCCP: Carbonyl cyanide m-chlorophenylhydrazone
CQ: Chloroquine
ELISA: Enzymelinked immunosorbent assay
ER: Endoplasmic reticulum
FAD-PS1: Familial AD-PS1
MMP: Mitochondrial membrane potential
PBS: Phosphate-buffered saline
PCR: Polymerase Chain Reaction
PM: Plasma membrane
SAP: Spontaneous alternations preference
sAPPα: Secreted amyloid-β precursor protein α
TEM: Transmission electron microscopy
WT: Wild-type

## References

[1] McKhann G, Drachman D, Folstein M, Katzman R, Price D, Stadlan EM. Clinical diagnosis of Alzheimer’s disease: report of the NINCDS-ADRDA Work Group under the auspices of Department of Health and Human Services Task Force on Alzheimer’s Disease. Neurology. 1984;34(7):939–44. 10.1212/wnl.34.7.939. 6610841 10.1212/wnl.34.7.939

[2] Hardy JA, Higgins GA. Alzheimer’s disease: the amyloid cascade hypothesis. Science. 1992;256(5054):184–5. 10.1126/science.1566067. 1566067 10.1126/science.1566067

[3] Glenner GG, Wong CW. Alzheimer’s disease: initial report of the purification and characterization of a novel cerebrovascular amyloid protein. Biochem Biophys Res Commun. 1984;120(3):885–90. 10.1016/s0006-291x(84)80190-4. 6375662 10.1016/s0006-291x(84)80190-4

[4] Selkoe DJ. Alzheimer’s disease: genes, proteins, and therapy. Physiol Rev. 2001;81(2):741–66. 10.1152/physrev.2001.81.2.741. 11274343 10.1152/physrev.2001.81.2.741

[5] Ito K, Tatebe T, Suzuki K, Hirayama T, Hayakawa M, Kubo H, et al. Memantine reduces the production of amyloid-β peptides through modulation of amyloid precursor protein trafficking. Eur J Pharmacol. 2017;798:16–25. 10.1016/j.ejphar.2017.02.001. 28167259 10.1016/j.ejphar.2017.02.001

[6] Takata K, Kitamura Y, Saeki M, Terada M, Kagitani S, Kitamura R, et al. Galantamine-induced amyloid-{beta} clearance mediated via stimulation of microglial nicotinic acetylcholine receptors. J Biol Chem. 2010;285(51):40180–91. 10.1074/jbc.M110.142356. 20947502 10.1074/jbc.M110.142356 PMC3001000

[7] Bhattacharya S, Haertel C, Maelicke A, Montag D. Galantamine slows down plaque formation and behavioral decline in the 5XFAD mouse model of Alzheimer’s disease. PLoS ONE. 2014;9(2):e89454. 10.1371/journal.pone.0089454. 24586789 10.1371/journal.pone.0089454 PMC3931790

[8] Mintun MA, Wessels AM, Sims JR. Donanemab in Early Alzheimer’s Disease. Reply. N Engl J Med. 2021;385(7):667. 10.1056/NEJMc2109455. 34379934 10.1056/NEJMc2109455

[9] van Dyck CH, Swanson CJ, Aisen P, Bateman RJ, Chen C, Gee M, et al. Lecanemab in Early Alzheimer’s Disease. N Engl J Med. 2023;388(1):9–21. 10.1056/NEJMoa2212948. 36449413 10.1056/NEJMoa2212948

[10] Bell KF, Zheng L, Fahrenholz F, Cuello AC. ADAM-10 over-expression increases cortical synaptogenesis. Neurobiol Aging. 2008;29(4):554–65. 10.1016/j.neurobiolaging.2006.11.004. 17187903 10.1016/j.neurobiolaging.2006.11.004

[11] Müller UC, Zheng H. Physiological functions of APP family proteins. Cold Spring Harb Perspect Med. 2012;2(2):a006288. 10.1101/cshperspect.a006288. 22355794 10.1101/cshperspect.a006288 PMC3281588

[12] Sosa LJ, Cáceres A, Dupraz S, Oksdath M, Quiroga S, Lorenzo A. The physiological role of the amyloid precursor protein as an adhesion molecule in the developing nervous system. J Neurochem. 2017;143(1):11–29. 10.1111/jnc.14122. 28677143 10.1111/jnc.14122

[13] Zhang X, Song W. The role of APP and BACE1 trafficking in APP processing and amyloid-β generation. Alzheimers Res Ther. 2013;5(5):46. 10.1186/alzrt211. 24103387 10.1186/alzrt211 PMC3978418

[14] Zhang Y, Chen H, Li R, Sterling K, Song W. Amyloid β-based therapy for Alzheimer’s disease: challenges, successes and future. Signal Transduct Target Ther. 2023;8(1):248. 10.1038/s41392-023-01484-7. 37386015 10.1038/s41392-023-01484-7 PMC10310781

[15] Esch FS, Keim PS, Beattie EC, Blacher RW, Culwell AR, Oltersdorf T, et al. Cleavage of amyloid beta peptide during constitutive processing of its precursor. Science. 1990;248(4959):1122–4. 10.1126/science.2111583. 2111583 10.1126/science.2111583

[16] Lammich S, Kojro E, Postina R, Gilbert S, Pfeiffer R, Jasionowski M, et al. Constitutive and regulated alpha-secretase cleavage of Alzheimer’s amyloid precursor protein by a disintegrin metalloprotease. Proc Natl Acad Sci U S A. 1999;96(7):3922–7. 10.1073/pnas.96.7.3922. 10097139 10.1073/pnas.96.7.3922 PMC22396

[17] Buxbaum JD, Liu KN, Luo Y, Slack JL, Stocking KL, Peschon JJ, et al. Evidence that tumor necrosis factor alpha converting enzyme is involved in regulated alpha-secretase cleavage of the Alzheimer amyloid protein precursor. J Biol Chem. 1998;273(43):27765–7. 10.1074/jbc.273.43.27765. 9774383 10.1074/jbc.273.43.27765

[18] Meziane H, Dodart JC, Mathis C, Little S, Clemens J, Paul SM, et al. Memory-enhancing effects of secreted forms of the beta-amyloid precursor protein in normal and amnestic mice. Proc Natl Acad Sci U S A. 1998;95(21):12683–8. 10.1073/pnas.95.21.12683. 9770546 10.1073/pnas.95.21.12683 PMC22891

[19] Ring S, Weyer SW, Kilian SB, Waldron E, Pietrzik CU, Filippov MA, et al. The secreted beta-amyloid precursor protein ectodomain APPs alpha is sufficient to rescue the anatomical, behavioral, and electrophysiological abnormalities of APP-deficient mice. J Neurosci. 2007;27(29):7817–26. 10.1523/jneurosci.1026-07.2007. 17634375 10.1523/jneurosci.1026-07.2007 PMC6672885

[20] Tyan SH, Shih AY, Walsh JJ, Maruyama H, Sarsoza F, Ku L, et al. Amyloid precursor protein (APP) regulates synaptic structure and function. Mol Cell Neurosci. 2012;51(1–2):43–52. 10.1016/j.mcn.2012.07.009. 22884903 10.1016/j.mcn.2012.07.009 PMC3538857

[21] Milosch N, Tanriöver G, Kundu A, Rami A, François JC, Baumkötter F, et al. Holo-APP and G-protein-mediated signaling are required for sAPPα-induced activation of the akt survival pathway. Cell Death Dis. 2014;5(8):e1391. 10.1038/cddis.2014.352. 25165877 10.1038/cddis.2014.352 PMC4454324

[22] Mockett BG, Richter M, Abraham WC, Müller UC. Therapeutic potential of secreted amyloid precursor protein APPsα. Front Mol Neurosci. 2017;10:30. 10.3389/fnmol.2017.00030. 28223920 10.3389/fnmol.2017.00030 PMC5293819

[23] Vassar R, Bennett BD, Babu-Khan S, Kahn S, Mendiaz EA, Denis P, et al. Beta-secretase cleavage of Alzheimer’s amyloid precursor protein by the transmembrane aspartic protease BACE. Science. 1999;286(5440):735–41. 10.1126/science.286.5440.735. 10531052 10.1126/science.286.5440.735

[24] Sun X, Bromley-Brits K, Song W. Regulation of β-site APP-cleaving enzyme 1 gene expression and its role in Alzheimer’s disease. J Neurochem. 2012;120(s1):62–70. 10.1111/j.1471-4159.2011.07515.x. 22122349 10.1111/j.1471-4159.2011.07515.x

[25] Deng Y, Wang Z, Wang R, Zhang X, Zhang S, Wu Y, et al. Amyloid-β protein (Aβ) Glu11 is the major β-secretase site of β-site amyloid-β precursor protein-cleaving enzyme 1(BACE1), and shifting the cleavage site to Aβ Asp1 contributes to Alzheimer pathogenesis. Eur J Neurosci. 2013;37(12):1962–9. 10.1111/ejn.12235. 23773065 10.1111/ejn.12235

[26] Li Y, Zhou W, Tong Y, He G, Song W. Control of APP processing and abeta generation level by BACE1 enzymatic activity and transcription. Faseb j. 2006;20(2):285–92. 10.1096/fj.05-4986com. 16449801 10.1096/fj.05-4986com

[27] Moussa-Pacha NM, Abdin SM, Omar HA, Alniss H, Al-Tel TH. BACE1 inhibitors: current status and future directions in treating Alzheimer’s disease. Med Res Rev. 2020;40(1):339–84. 10.1002/med.21622. 31347728 10.1002/med.21622

[28] Reed LJ, De BB, Gunsalus IC, Hornberger CS. Jr. Crystalline alpha-lipoic acid; a catalytic agent associated with pyruvate dehydrogenase. Science. 1951;114(2952):93–4. 10.1126/science.114.2952.93. 14854913 10.1126/science.114.2952.93

[29] Panigrahi M, Sadguna Y, Shivakumar BR, Kolluri SV, Roy S, Packer L, et al. Alpha-lipoic acid protects against reperfusion injury following cerebral ischemia in rats. Brain Res. 1996;717(1–2):184–8. 10.1016/0006-8993(96)00009-1. 8738270 10.1016/0006-8993(96)00009-1

[30] Ahmed MA, El-Awdan SA. Lipoic acid and pentoxifylline mitigate nandrolone decanoate-induced neurobehavioral perturbations in rats via re-balance of brain neurotransmitters, up-regulation of Nrf2/HO-1 pathway, and down-regulation of TNFR1 expression. Horm Behav. 2015;73:186–99. 10.1016/j.yhbeh.2015.07.007. 26187709 10.1016/j.yhbeh.2015.07.007

[31] Gorąca A, Huk-Kolega H, Piechota A, Kleniewska P, Ciejka E, Skibska B. Lipoic acid - biological activity and therapeutic potential. Pharmacol Rep. 2011;63(4):849–58. 10.1016/s1734-1140(11)70600-4. 22001972 10.1016/s1734-1140(11)70600-4

[32] Ziegler D, Nowak H, Kempler P, Vargha P, Low PA. Treatment of symptomatic diabetic polyneuropathy with the antioxidant alpha-lipoic acid: a meta-analysis. Diabet Med. 2004;21(2):114–21. 10.1111/j.1464-5491.2004.01109.x. 14984445 10.1111/j.1464-5491.2004.01109.x

[33] Hager K, Marahrens A, Kenklies M, Riederer P, Münch G. Alpha-lipoic acid as a new treatment option for Alzheimer [corrected] type dementia. Arch Gerontol Geriatr. 2001;32(3):275–82. 10.1016/s0167-4943(01)00104-2. 11395173 10.1016/s0167-4943(01)00104-2

[34] Pei X, Hu F, Hu Z, Luo F, Li X, Xing S, et al. Neuroprotective effect of α-Lipoic acid against Aβ(25–35)-Induced damage in BV2 cells. Molecules. 2023;28(3):1168. 10.3390/molecules28031168. 36770835 10.3390/molecules28031168 PMC9919339

[35] Liu J. The effects and mechanisms of mitochondrial nutrient alpha-lipoic acid on improving age-associated mitochondrial and cognitive dysfunction: an overview. Neurochem Res. 2008;33(1):194–203. 10.1007/s11064-007-9403-0. 17605107 10.1007/s11064-007-9403-0

[36] Suh JH, Wang H, Liu RM, Liu J, Hagen TM. (R)-alpha-lipoic acid reverses the age-related loss in GSH redox status in post-mitotic tissues: evidence for increased cysteine requirement for GSH synthesis. Arch Biochem Biophys. 2004;423(1):126–35. 10.1016/j.abb.2003.12.020. 14871476 10.1016/j.abb.2003.12.020 PMC4696556

[37] Maczurek A, Hager K, Kenklies M, Sharman M, Martins R, Engel J, et al. Lipoic acid as an anti-inflammatory and neuroprotective treatment for Alzheimer’s disease. Adv Drug Deliv Rev. 2008;60(13–14):1463–70. 10.1016/j.addr.2008.04.015. 18655815 10.1016/j.addr.2008.04.015

[38] Sancheti H, Kanamori K, Patil I, Díaz Brinton R, Ross BD, Cadenas E. Reversal of metabolic deficits by lipoic acid in a triple transgenic mouse model of Alzheimer’s disease: a 13 C NMR study. J Cereb Blood Flow Metab. 2014;34(2):288–96. 10.1038/jcbfm.2013.196. 24220168 10.1038/jcbfm.2013.196 PMC3915206

[39] Song G, Liu Z, Wang L, Shi R, Chu C, Xiang M, et al. Protective effects of lipoic acid against acrylamide-induced neurotoxicity: involvement of mitochondrial energy metabolism and autophagy. Food Funct. 2017;8(12):4657–67. 10.1039/c7fo01429e. 29159335 10.1039/c7fo01429e

[40] Pickles S, Vigié P, Youle RJ. Mitophagy and Quality Control mechanisms in mitochondrial maintenance. Curr Biol. 2018;28(4):R170–85. 10.1016/j.cub.2018.01.004. 29462587 10.1016/j.cub.2018.01.004 PMC7255410

[41] Hamasaki M, Furuta N, Matsuda A, Nezu A, Yamamoto A, Fujita N, et al. Autophagosomes form at ER-mitochondria contact sites. Nature. 2013;495(7441):389–93. 10.1038/nature11910. 23455425 10.1038/nature11910

[42] Tooze SA, Yoshimori T. The origin of the autophagosomal membrane. Nat Cell Biol. 2010;12(9):831–5. 10.1038/ncb0910-831. 20811355 10.1038/ncb0910-831

[43] Caporaso GL, Takei K, Gandy SE, Matteoli M, Mundigl O, Greengard P, et al. Morphologic and biochemical analysis of the intracellular trafficking of the Alzheimer beta/A4 amyloid precursor protein. J Neurosci. 1994;14(5 Pt 2):3122–38. 10.1523/jneurosci.14-05-03122.1994. 8182461 10.1523/jneurosci.14-05-03122.1994 PMC6577440

[44] Koo EH, Squazzo SL, Selkoe DJ, Koo CH. Trafficking of cell-surface amyloid beta-protein precursor. I. Secretion, endocytosis and recycling as detected by labeled monoclonal antibody. J Cell Sci. 1996;109(Pt 5):991–8. 10.1242/jcs.109.5.991. 8743946 10.1242/jcs.109.5.991

[45] Perez RG, Soriano S, Hayes JD, Ostaszewski B, Xia W, Selkoe DJ, et al. Mutagenesis identifies new signals for beta-amyloid precursor protein endocytosis, turnover, and the generation of secreted fragments, including Abeta42. J Biol Chem. 1999;274(27):18851–6. 10.1074/jbc.274.27.18851. 10383380 10.1074/jbc.274.27.18851

[46] Koo EH, Squazzo SL. Evidence that production and release of amyloid beta-protein involves the endocytic pathway. J Biol Chem. 1994;269(26):17386–9. http://doi.org/https://pubmed.ncbi.nlm.nih.gov/8021238/. 8021238 10.1016/S0021-9258(17)32449-3

[47] Lai A, Sisodia SS, Trowbridge IS. Characterization of sorting signals in the beta-amyloid precursor protein cytoplasmic domain. J Biol Chem. 1995;270(8):3565–73. http://doi.org/https://pubmed.ncbi.nlm.nih.gov/7876092/. 7876092 10.1074/jbc.270.8.3565

[48] Haass C, Koo EH, Mellon A, Hung AY, Selkoe DJ. Targeting of cell-surface beta-amyloid precursor protein to lysosomes: alternative processing into amyloid-bearing fragments. Nature. 1992;357(6378):500–3. 10.1038/357500a0. 1608449 10.1038/357500a0

[49] Anders A, Gilbert S, Garten W, Postina R, Fahrenholz F. Regulation of the alpha-secretase ADAM10 by its prodomain and proprotein convertases. Faseb j. 2001;15(10):1837–9. 10.1096/fj.01-0007fje. 11481247 10.1096/fj.01-0007fje

[50] Maretzky T, Evers A, Le Gall S, Alabi RO, Speck N, Reiss K, et al. The cytoplasmic domain of a disintegrin and metalloproteinase 10 (ADAM10) regulates its constitutive activity but is dispensable for stimulated ADAM10-dependent shedding. J Biol Chem. 2015;290(12):7416–25. 10.1074/jbc.M114.603753. 25605720 10.1074/jbc.M114.603753 PMC4367251

[51] Vingtdeux V, Marambaud P. Identification and biology of α-secretase. J Neurochem. 2012;120(Suppl 1):34–45. 10.1111/j.1471-4159.2011.07477.x. 22121879 10.1111/j.1471-4159.2011.07477.x

[52] Parvathy S, Hussain I, Karran EH, Turner AJ, Hooper NM. Cleavage of Alzheimer’s amyloid precursor protein by alpha-secretase occurs at the surface of neuronal cells. Biochemistry. 1999;38(30):9728–34. 10.1021/bi9906827. 10423252 10.1021/bi9906827

[53] Capell A, Steiner H, Willem M, Kaiser H, Meyer C, Walter J, et al. Maturation and pro-peptide cleavage of beta-secretase. J Biol Chem. 2000;275(40):30849–54. 10.1074/jbc.M003202200. 10801872 10.1074/jbc.M003202200

[54] Ly PT, Wu Y, Zou H, Wang R, Zhou W, Kinoshita A, et al. Inhibition of GSK3β-mediated BACE1 expression reduces Alzheimer-associated phenotypes. J Clin Invest. 2013;123(1):224–35. 10.1172/jci64516. 23202730 10.1172/jci64516 PMC3533290

[55] Bromley-Brits K, Deng Y, Song W. Morris water maze test for learning and memory deficits in Alzheimer’s disease model mice. J Vis Exp. 2011;53:2920. 10.3791/2920. 10.3791/2920 PMC3347885 21808223

[56] Zhou W, Li X, Huang D, Zhou W, Li T, Song W. No significant effect of 7,8-dihydroxyflavone on APP processing and Alzheimer-associated phenotypes. Curr Alzheimer Res. 2015;12(1):47–52. 10.2174/1567205012666141218124243. 25523427 10.2174/1567205012666141218124243

[57] Kimura S, Noda T, Yoshimori T. Dissection of the autophagosome maturation process by a novel reporter protein, tandem fluorescent-tagged LC3. Autophagy. 2007;3(5):452–60. 10.4161/auto.4451. 17534139 10.4161/auto.4451

[58] Mauthe M, Orhon I, Rocchi C, Zhou X, Luhr M, Hijlkema KJ, et al. Chloroquine inhibits autophagic flux by decreasing autophagosome-lysosome fusion. Autophagy. 2018;14(8):1435–55. 10.1080/15548627.2018.1474314. 29940786 10.1080/15548627.2018.1474314 PMC6103682

[59] Li F, Zhu Y, Sun X, Hu H, Zhou M, Bai Y, et al. Diethylhexyl phthalate induces anxiety-like behavior and learning and memory impairment in mice probably by damaging blood-brain barrier. Nan Fang Yi Ke Da Xue Xue Bao. 2022;42(8):1237–43. 10.12122/j.issn.1673-4254.2022.08.17. 36073224 10.12122/j.issn.1673-4254.2022.08.17 PMC9458531

[60] Aparna S, Patri M. Benzo[a]pyrene exposure and overcrowding stress impacts anxiety-like behavior and impairs learning and memory in adult zebrafish, Danio rerio. Environ Toxicol. 2021;36(3):352–61. 10.1002/tox.23041. 33280238 10.1002/tox.23041

[61] Delphin-Combe F, Bathsavanis A, Rouch I, Liles T, Vannier-Nitenberg C, Fantino B, et al. Relationship between anxiety and cognitive performance in an elderly population with a cognitive complaint. Eur J Neurol. 2016;23(7):1210–7. 10.1111/ene.13004. 27106897 10.1111/ene.13004

[62] Borchelt DR, Thinakaran G, Eckman CB, Lee MK, Davenport F, Ratovitsky T, et al. Familial Alzheimer’s disease-linked presenilin 1 variants elevate Abeta1-42/1–40 ratio in vitro and in vivo. Neuron. 1996;17(5):1005–13. 10.1016/s0896-6273(00)80230-5. 8938131 10.1016/s0896-6273(00)80230-5

[63] Citron M, Westaway D, Xia W, Carlson G, Diehl T, Levesque G, et al. Mutant presenilins of Alzheimer’s disease increase production of 42-residue amyloid beta-protein in both transfected cells and transgenic mice. Nat Med. 1997;3(1):67–72. 10.1038/nm0197-67. 8986743 10.1038/nm0197-67

[64] Lamb BT, Bardel KA, Kulnane LS, Anderson JJ, Holtz G, Wagner SL, et al. Amyloid production and deposition in mutant amyloid precursor protein and presenilin-1 yeast artificial chromosome transgenic mice. Nat Neurosci. 1999;2(8):695–7. 10.1038/11154. 10412057 10.1038/11154

[65] Jankowsky JL, Fadale DJ, Anderson J, Xu GM, Gonzales V, Jenkins NA, et al. Mutant presenilins specifically elevate the levels of the 42 residue beta-amyloid peptide in vivo: evidence for augmentation of a 42-specific gamma secretase. Hum Mol Genet. 2004;13(2):159–70. 10.1093/hmg/ddh019. 14645205 10.1093/hmg/ddh019

[66] Jarrett JT, Berger EP, Lansbury PT Jr. The carboxy terminus of the beta amyloid protein is critical for the seeding of amyloid formation: implications for the pathogenesis of Alzheimer’s disease. Biochemistry. 1993;32(18):4693–7. 10.1021/bi00069a001. 8490014 10.1021/bi00069a001

[67] Iwatsubo T, Odaka A, Suzuki N, Mizusawa H, Nukina N, Ihara Y. Visualization of a beta 42(43) and a beta 40 in senile plaques with end-specific A beta monoclonals: evidence that an initially deposited species is a beta 42(43). Neuron. 1994;13(1):45–53. 10.1016/0896-6273(94)90458-8. 8043280 10.1016/0896-6273(94)90458-8

[68] Azevedo FA, Carvalho LR, Grinberg LT, Farfel JM, Ferretti RE, Leite RE, et al. Equal numbers of neuronal and nonneuronal cells make the human brain an isometrically scaled-up primate brain. J Comp Neurol. 2009;513(5):532–41. 10.1002/cne.21974. 19226510 10.1002/cne.21974

[69] von Bartheld CS, Bahney J, Herculano-Houzel S. The search for true numbers of neurons and glial cells in the human brain: a review of 150 years of cell counting. J Comp Neurol. 2016;524(18):3865–95. 10.1002/cne.24040. 27187682 10.1002/cne.24040 PMC5063692

[70] Cai H, Wang Y, McCarthy D, Wen H, Borchelt DR, Price DL, et al. BACE1 is the major beta-secretase for generation of abeta peptides by neurons. Nat Neurosci. 2001;4(3):233–4. 10.1038/85064. 11224536 10.1038/85064

[71] Haass C, Kaether C, Thinakaran G, Sisodia S. Trafficking and proteolytic processing of APP. Cold Spring Harb Perspect Med. 2012;2(5):a006270. 10.1101/cshperspect.a006270. 22553493 10.1101/cshperspect.a006270 PMC3331683

[72] Kuhn PH, Wang H, Dislich B, Colombo A, Zeitschel U, Ellwart JW, et al. ADAM10 is the physiologically relevant, constitutive alpha-secretase of the amyloid precursor protein in primary neurons. Embo j. 2010;29(17):3020–32. 10.1038/emboj.2010.167. 20676056 10.1038/emboj.2010.167 PMC2944055

[73] Portelius E, Price E, Brinkmalm G, Stiteler M, Olsson M, Persson R, et al. A novel pathway for amyloid precursor protein processing. Neurobiol Aging. 2011;32(6):1090–8. 10.1016/j.neurobiolaging.2009.06.002. 19604603 10.1016/j.neurobiolaging.2009.06.002

[74] Marcello E, Borroni B, Pelucchi S, Gardoni F, Di Luca M. ADAM10 as a therapeutic target for brain diseases: from developmental disorders to Alzheimer’s disease. Expert Opin Ther Targets. 2017;21(11):1017–26. 10.1080/14728222.2017.1386176. 28960088 10.1080/14728222.2017.1386176

[75] Lichtenthaler SF. Alpha-secretase cleavage of the amyloid precursor protein: proteolysis regulated by signaling pathways and protein trafficking. Curr Alzheimer Res. 2012;9(2):165–77. 10.2174/156720512799361655. 21605033 10.2174/156720512799361655

[76] Ludwig A, Hundhausen C, Lambert MH, Broadway N, Andrews RC, Bickett DM, et al. Metalloproteinase inhibitors for the disintegrin-like metalloproteinases ADAM10 and ADAM17 that differentially block constitutive and phorbol ester-inducible shedding of cell surface molecules. Comb Chem High Throughput Screen. 2005;8(2):161–71. 10.2174/1386207053258488. 15777180 10.2174/1386207053258488

[77] Gearing AJ, Beckett P, Christodoulou M, Churchill M, Clements J, Davidson AH, et al. Processing of tumour necrosis factor-alpha precursor by metalloproteinases. Nature. 1994;370(6490):555–7. 10.1038/370555a0. 8052310 10.1038/370555a0

[78] Matthews V, Schuster B, Schütze S, Bussmeyer I, Ludwig A, Hundhausen C, et al. Cellular cholesterol depletion triggers shedding of the human interleukin-6 receptor by ADAM10 and ADAM17 (TACE). J Biol Chem. 2003;278(40):38829–39. 10.1074/jbc.M210584200. 12832423 10.1074/jbc.M210584200

[79] Allinson TM, Parkin ET, Condon TP, Schwager SL, Sturrock ED, Turner AJ, et al. The role of ADAM10 and ADAM17 in the ectodomain shedding of angiotensin converting enzyme and the amyloid precursor protein. Eur J Biochem. 2004;271(12):2539–47. 10.1111/j.1432-1033.2004.04184.x. 15182369 10.1111/j.1432-1033.2004.04184.x

[80] Zhang Q, Xing M, Bao Z, Xu L, Bai Y, Chen W, et al. Contactin-associated protein-like 2 (CNTNAP2) mutations impair the essential α-secretase cleavages, leading to autism-like phenotypes. Signal Transduct Target Ther. 2024;9(1):51. 10.1038/s41392-024-01768-6. 38424048 10.1038/s41392-024-01768-6 PMC10904759

[81] Caescu CI, Jeschke GR, Turk BE. Active-site determinants of substrate recognition by the metalloproteinases TACE and ADAM10. Biochem J. 2009;424(1):79–88. 10.1042/bj20090549. 19715556 10.1042/bj20090549 PMC2774824

[82] Hundhausen C, Misztela D, Berkhout TA, Broadway N, Saftig P, Reiss K, et al. The disintegrin-like metalloproteinase ADAM10 is involved in constitutive cleavage of CX3CL1 (fractalkine) and regulates CX3CL1-mediated cell-cell adhesion. Blood. 2003;102(4):1186–95. 10.1182/blood-2002-12-3775. 12714508 10.1182/blood-2002-12-3775

[83] Postina R, Schroeder A, Dewachter I, Bohl J, Schmitt U, Kojro E, et al. A disintegrin-metalloproteinase prevents amyloid plaque formation and hippocampal defects in an Alzheimer disease mouse model. J Clin Invest. 2004;113(10):1456–64. 10.1172/jci20864. 15146243 10.1172/jci20864 PMC406531

[84] Skovronsky DM, Moore DB, Milla ME, Doms RW, Lee VM. Protein kinase C-dependent alpha-secretase competes with beta-secretase for cleavage of amyloid-beta precursor protein in the trans-golgi network. J Biol Chem. 2000;275(4):2568–75. 10.1074/jbc.275.4.2568. 10644715 10.1074/jbc.275.4.2568

[85] Citron M, Oltersdorf T, Haass C, McConlogue L, Hung AY, Seubert P, et al. Mutation of the β-amyloid precursor protein in familial Alzheimer’s disease increases β-protein production. Nature. 1992;360(6405):672–4. 10.1038/360672a0. 1465129 10.1038/360672a0

[86] Cai XD, Golde TE, Younkin SG. Release of excess amyloid beta protein from a mutant amyloid beta protein precursor. Science. 1993;259(5094):514–6. 10.1126/science.8424174. 8424174 10.1126/science.8424174

[87] Suzuki N, Cheung TT, Cai XD, Odaka A, Otvos L Jr., Eckman C, et al. An increased percentage of long amyloid beta protein secreted by familial amyloid beta protein precursor (beta APP717) mutants. Science. 1994;264(5163):1336–40. 10.1126/science.8191290. 8191290 10.1126/science.8191290

[88] Haass C, Hung AY, Selkoe DJ, Teplow DB. Mutations associated with a locus for familial Alzheimer’s disease result in alternative processing of amyloid beta-protein precursor. J Biol Chem. 1994;269(26):17741–8. 8021287 10.1016/S0021-9258(17)32503-6

[89] Fahrenholz F. Alpha-secretase as a therapeutic target. Curr Alzheimer Res. 2007;4(4):412–7. 10.2174/156720507781788837. 17908044 10.2174/156720507781788837

[90] Lichtenthaler SF. α-secretase in Alzheimer’s disease: molecular identity, regulation and therapeutic potential. J Neurochem. 2011;116(1):10–21. 10.1111/j.1471-4159.2010.07081.x. 21044078 10.1111/j.1471-4159.2010.07081.x

[91] Brummer T, Pigoni M, Rossello A, Wang H, Noy PJ, Tomlinson MG, et al. The metalloprotease ADAM10 (a disintegrin and metalloprotease 10) undergoes rapid, postlysis autocatalytic degradation. Faseb j. 2018;32(7):3560–73. 10.1096/fj.201700823RR. 29430990 10.1096/fj.201700823RR PMC5998973

[92] Jefferson T, Auf dem Keller U, Bellac C, Metz VV, Broder C, Hedrich J, et al. The substrate degradome of meprin metalloproteases reveals an unexpected proteolytic link between meprin β and ADAM10. Cell Mol Life Sci. 2013;70(2):309–33. 10.1007/s00018-012-1106-2. 22940918 10.1007/s00018-012-1106-2 PMC3535375

[93] Wang H, Muthu Karuppan MK, Nair M, Lakshmana MK. Autophagy-dependent increased ADAM10 mature protein Induced by TFEB overexpression is mediated through PPARα. Mol Neurobiol. 2021;58(5):2269–83. 10.1007/s12035-020-02230-8. 33417226 10.1007/s12035-020-02230-8 PMC8026501

[94] Zhang X, Tang L, Zhang Z. ADAM10 and ADAM17 are degraded by lysosomal pathway via asparagine endopeptidase. Biochem Biophys Res Commun. 2021;537:15–21. 10.1016/j.bbrc.2020.12.063. 33383559 10.1016/j.bbrc.2020.12.063

[95] Nixon RA, Wegiel J, Kumar A, Yu WH, Peterhoff C, Cataldo A, et al. Extensive involvement of autophagy in Alzheimer disease: an immuno-electron microscopy study. J Neuropathol Exp Neurol. 2005;64(2):113–22. 10.1093/jnen/64.2.113. 15751225 10.1093/jnen/64.2.113

[96] Menzies FM, Fleming A, Caricasole A, Bento CF, Andrews SP, Ashkenazi A, et al. Autophagy and neurodegeneration: pathogenic mechanisms and Therapeutic opportunities. Neuron. 2017;93(5):1015–34. 10.1016/j.neuron.2017.01.022. 28279350 10.1016/j.neuron.2017.01.022

[97] Caccamo A, Majumder S, Richardson A, Strong R, Oddo S. Molecular interplay between mammalian target of rapamycin (mTOR), amyloid-beta, and tau: effects on cognitive impairments. J Biol Chem. 2010;285(17):13107–20. 10.1074/jbc.M110.100420. 20178983 10.1074/jbc.M110.100420 PMC2857107

[98] Lee JH, Yang DS, Goulbourne CN, Im E, Stavrides P, Pensalfini A, et al. Faulty autolysosome acidification in Alzheimer’s disease mouse models induces autophagic build-up of Aβ in neurons, yielding senile plaques. Nat Neurosci. 2022;25(6):688–701. 10.1038/s41593-022-01084-8. 35654956 10.1038/s41593-022-01084-8 PMC9174056

[99] Guo Y, Guan T, Shafiq K, Yu Q, Jiao X, Na D, et al. Mitochondrial dysfunction in aging. Ageing Res Rev. 2023;88:101955. 10.1016/j.arr.2023.101955. 37196864 10.1016/j.arr.2023.101955

[100] Kerr JS, Adriaanse BA, Greig NH, Mattson MP, Cader MZ, Bohr VA, et al. Mitophagy and Alzheimer’s Disease: Cellular and Molecular mechanisms. Trends Neurosci. 2017;40(3):151–66. 10.1016/j.tins.2017.01.002. 28190529 10.1016/j.tins.2017.01.002 PMC5341618

[101] Yao J, Irwin RW, Zhao L, Nilsen J, Hamilton RT, Brinton RD. Mitochondrial bioenergetic deficit precedes Alzheimer’s pathology in female mouse model of Alzheimer’s disease. Proc Natl Acad Sci U S A. 2009;106(34):14670–5. 10.1073/pnas.0903563106. 19667196 10.1073/pnas.0903563106 PMC2732886

[102] Swerdlow RH, Khan SM. A mitochondrial cascade hypothesis for sporadic Alzheimer’s disease. Med Hypotheses. 2004;63(1):8–20. 10.1016/j.mehy.2003.12.045. 15193340 10.1016/j.mehy.2003.12.045

[103] Du H, Guo L, Yan S, Sosunov AA, McKhann GM, Yan SS. Early deficits in synaptic mitochondria in an Alzheimer’s disease mouse model. Proc Natl Acad Sci U S A. 2010;107(43):18670–5. 10.1073/pnas.1006586107. 20937894 10.1073/pnas.1006586107 PMC2972922

[104] Beck JS, Mufson EJ, Counts SE. Evidence for mitochondrial UPR Gene activation in familial and sporadic Alzheimer’s Disease. Curr Alzheimer Res. 2016;13(6):610–4. 10.2174/1567205013666151221145445. 26687188 10.2174/1567205013666151221145445 PMC5977398

[105] Kim I, Rodriguez-Enriquez S, Lemasters JJ. Selective degradation of mitochondria by mitophagy. Arch Biochem Biophys. 2007;462(2):245–53. 10.1016/j.abb.2007.03.034. 17475204 10.1016/j.abb.2007.03.034 PMC2756107

[106] Pradeepkiran JA, Reddy PH. Defective mitophagy in Alzheimer’s disease. Ageing Res Rev. 2020;64:101191. 10.1016/j.arr.2020.101191. 33022416 10.1016/j.arr.2020.101191 PMC7710581

[107] Wu X, Zheng Y, Liu M, Li Y, Ma S, Tang W, et al. BNIP3L/NIX degradation leads to mitophagy deficiency in ischemic brains. Autophagy. 2021;17(8):1934–46. 10.1080/15548627.2020.1802089. 32722981 10.1080/15548627.2020.1802089 PMC8386707

[108] Novak I, Kirkin V, McEwan DG, Zhang J, Wild P, Rozenknop A, et al. Nix is a selective autophagy receptor for mitochondrial clearance. EMBO Rep. 2010;11(1):45–51. 10.1038/embor.2009.256. 20010802 10.1038/embor.2009.256 PMC2816619

[109] Schweers RL, Zhang J, Randall MS, Loyd MR, Li W, Dorsey FC, et al. NIX is required for programmed mitochondrial clearance during reticulocyte maturation. Proc Natl Acad Sci U S A. 2007;104(49):19500–5. 10.1073/pnas.0708818104. 18048346 10.1073/pnas.0708818104 PMC2148318

[110] Sandoval H, Thiagarajan P, Dasgupta SK, Schumacher A, Prchal JT, Chen M, et al. Essential role for Nix in autophagic maturation of erythroid cells. Nature. 2008;454(7201):232–5. 10.1038/nature07006. 18454133 10.1038/nature07006 PMC2570948

[111] Narendra DP, Jin SM, Tanaka A, Suen DF, Gautier CA, Shen J, et al. PINK1 is selectively stabilized on impaired mitochondria to activate Parkin. PLoS Biol. 2010;8(1):e1000298. 10.1371/journal.pbio.1000298. 20126261 10.1371/journal.pbio.1000298 PMC2811155

[112] Liberman EA, Topaly VP, Tsofina LM, Jasaitis AA, Skulachev VP. Mechanism of coupling of oxidative phosphorylation and the membrane potential of mitochondria. Nature. 1969;222(5198):1076–8. 10.1038/2221076a0. 5787094 10.1038/2221076a0

[113] Narendra D, Tanaka A, Suen DF, Youle RJ. Parkin is recruited selectively to impaired mitochondria and promotes their autophagy. J Cell Biol. 2008;183(5):795–803. 10.1083/jcb.200809125. 19029340 10.1083/jcb.200809125 PMC2592826

[114] Elmore SP, Qian T, Grissom SF, Lemasters JJ. The mitochondrial permeability transition initiates autophagy in rat hepatocytes. Faseb j. 2001;15(12):2286–7. 10.1096/fj.01-0206fje. 11511528 10.1096/fj.01-0206fje

[115] Fang EF, Hou Y, Palikaras K, Adriaanse BA, Kerr JS, Yang B, et al. Mitophagy inhibits amyloid-β and tau pathology and reverses cognitive deficits in models of Alzheimer’s disease. Nat Neurosci. 2019;22(3):401–12. 10.1038/s41593-018-0332-9. 30742114 10.1038/s41593-018-0332-9 PMC6693625

[116] Du F, Yu Q, Yan S, Hu G, Lue LF, Walker DG, et al. PINK1 signalling rescues amyloid pathology and mitochondrial dysfunction in Alzheimer’s disease. Brain. 2017;140(12):3233–51. 10.1093/brain/awx258. 29077793 10.1093/brain/awx258 PMC5841141

[117] Mehrotra A, Sood A, Sandhir R. Mitochondrial modulators improve lipid composition and attenuate memory deficits in experimental model of Huntington’s disease. Mol Cell Biochem. 2015;410(1–2):281–92. 10.1007/s11010-015-2561-5. 26374445 10.1007/s11010-015-2561-5

[118] Eser Faki H, Tras B, Uney K. Alpha lipoic acid and vitamin E improve atorvastatin-induced mitochondrial dysfunctions in rats. Mitochondrion. 2020;52:83–8. 10.1016/j.mito.2020.02.011. 32119925 10.1016/j.mito.2020.02.011

[119] Memudu AE, Adewumi AE. Alpha lipoic acid ameliorates scopolamine induced memory deficit and neurodegeneration in the cerebello-hippocampal cortex. Metab Brain Dis. 2021;36(7):1729–45. 10.1007/s11011-021-00720-9. 34021876 10.1007/s11011-021-00720-9

[120] Hager K, Kenklies M, McAfoose J, Engel J, Münch G. Alpha-lipoic acid as a new treatment option for Alzheimer’s disease–a 48 months follow-up analysis. J Neural Transm Suppl. 2007;72:189–93. 10.1007/978-3-211-73574-9_24. 10.1007/978-3-211-73574-9_24 17982894

