# Supplementary material for: Alpha-lipoic acid alleviates cognitive deficits in transgenic APP23/PS45 mice through a mitophagy-mediated increase in ADAM10 α-secretase cleavage of APP
Source: Alzheimers Res Ther. 2024 Jul 19;16:160. doi: 10.1186/s13195-024-01527-3 (PMC11264788; doi:10.1186/s13195-024-01527-3)

Fig.4 C83 Fig.4 C83 marker (2023.3.28)


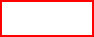

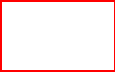

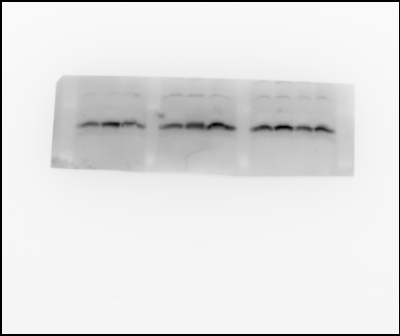

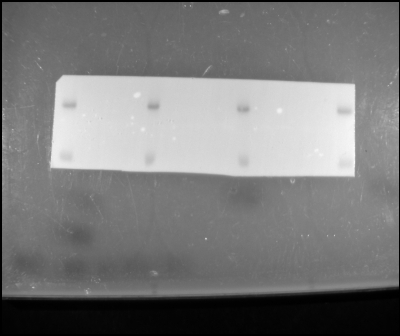


Fig.4 C83 Fig.4 C83 marker (2023.4.13)


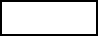

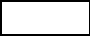

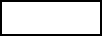

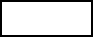

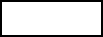

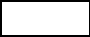

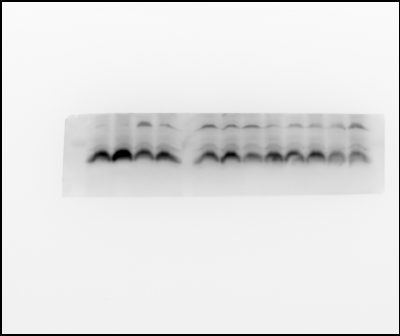

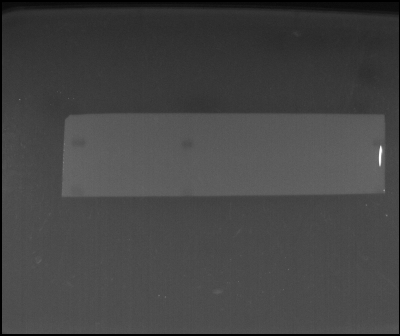


Fig.4 ADAM10 Fig.4 ADAM10 marker (2023.3.28)


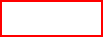

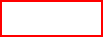

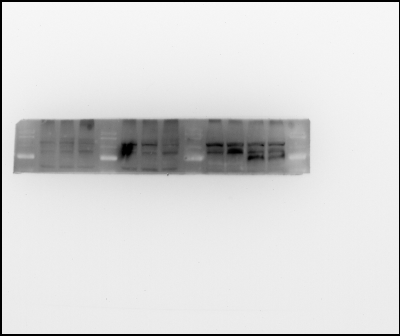

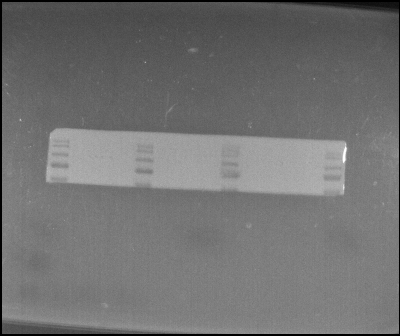


Fig.4 ADAM10 Fig.4 ADAM10 marker (2023.4.13)


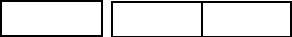

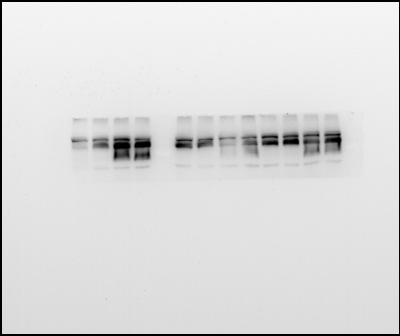

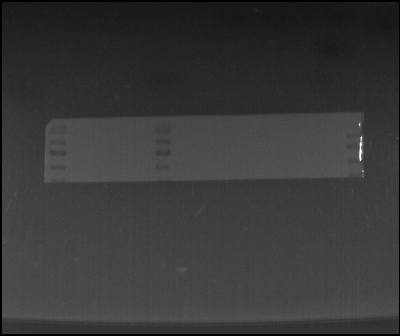


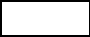

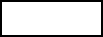

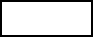


Fig.4 GAPDH Fig.4 GAPDH marker (2023.3.28)


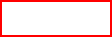

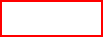

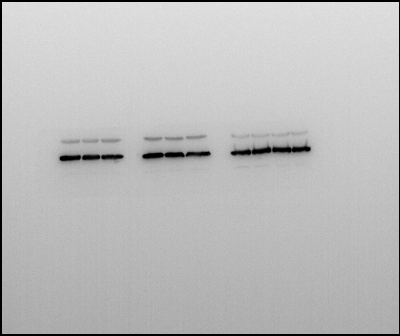

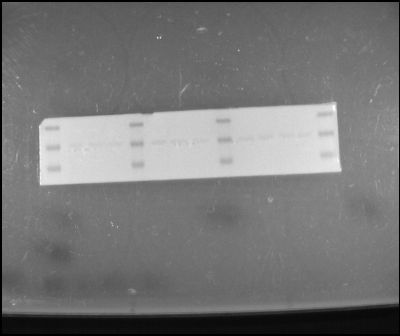


Fig.4 GAPDH Fig.4 GAPDH marker (2023.4.13)


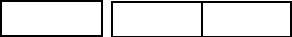

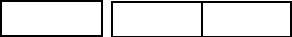

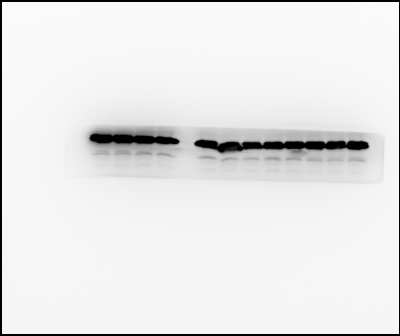

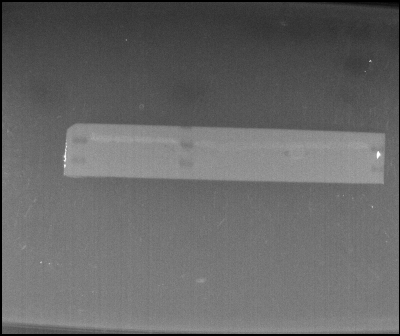

Supplement: Supplementary file 1 — Supplementary Material 1 [file 13195_2024_1527_MOESM1_ESM.docx]
