# Supplementary material for: Alpha-lipoic acid alleviates cognitive deficits in transgenic APP23/PS45 mice through a mitophagy-mediated increase in ADAM10 α-secretase cleavage of APP
Source: Alzheimers Res Ther. 2024 Jul 19;16:160. doi: 10.1186/s13195-024-01527-3 (PMC11264788; doi:10.1186/s13195-024-01527-3)

Fig.2E APP Fig.2E APP marker


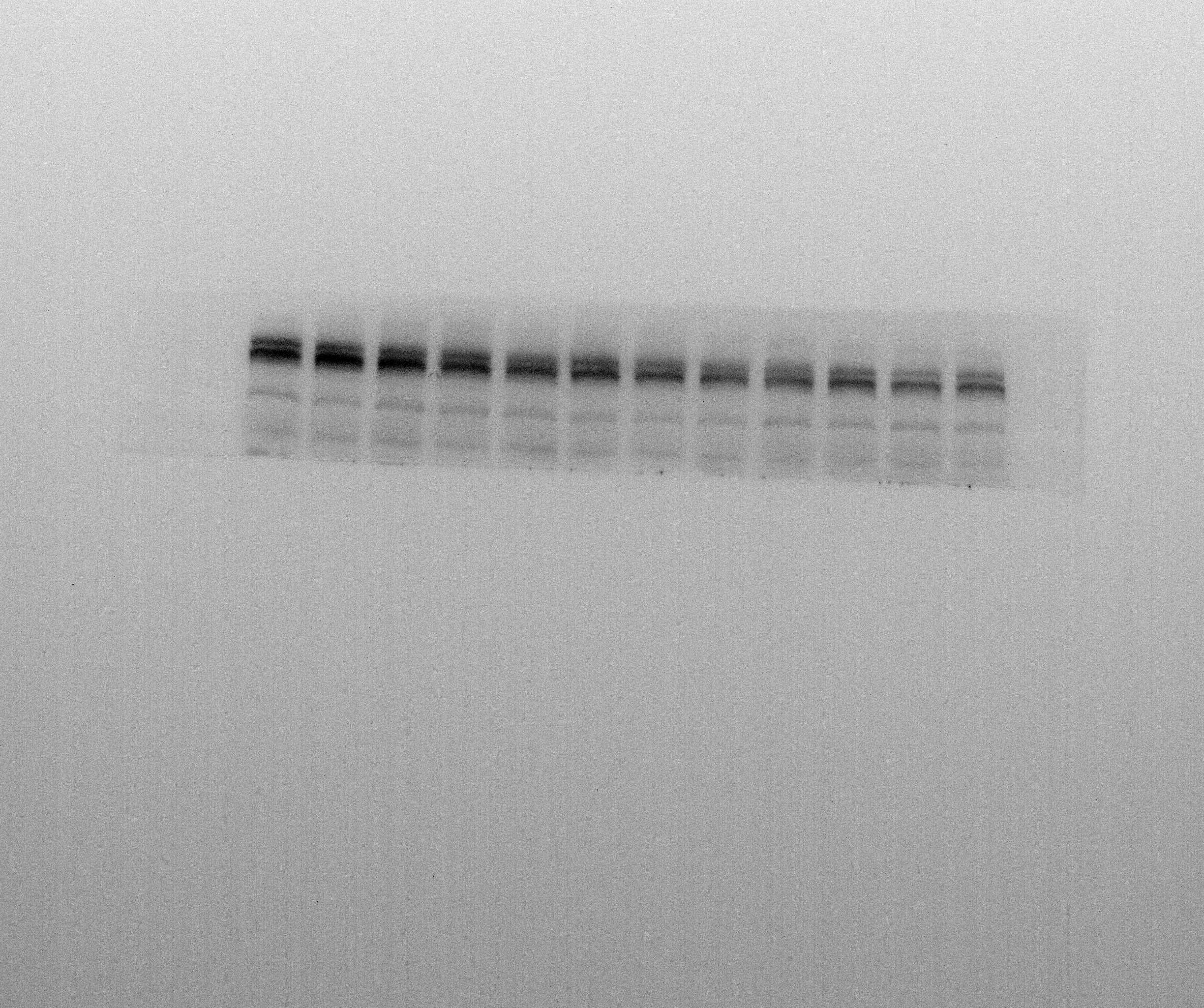

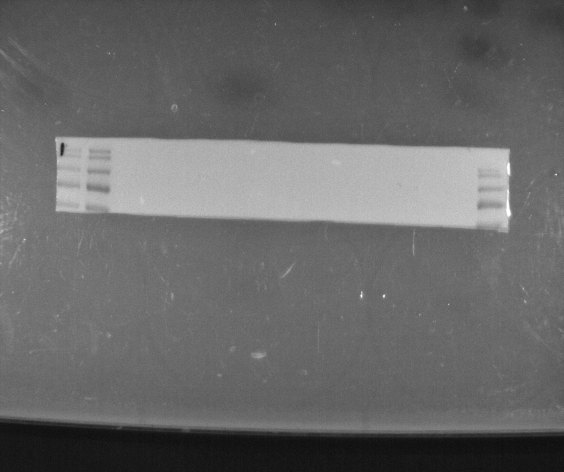


Fig.2E C99 C89 Fig.2E C99 C89 marker


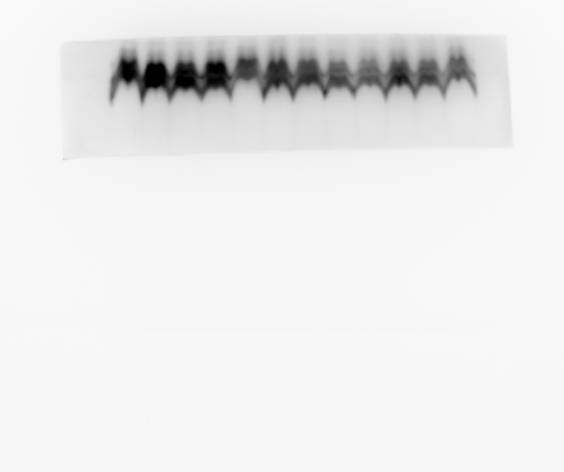

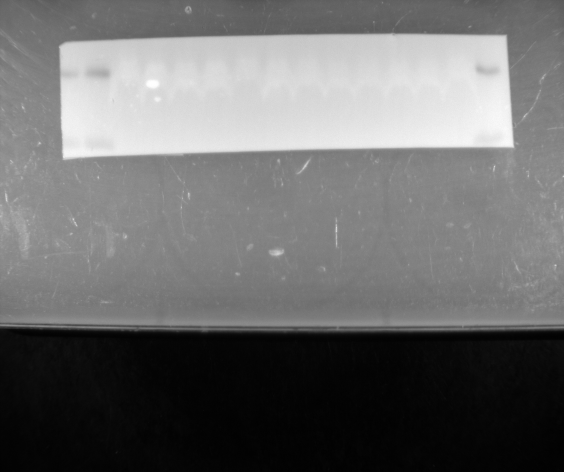


Fig.2E ACTIN Fig.2E ACTIN marker (2023.3.24)


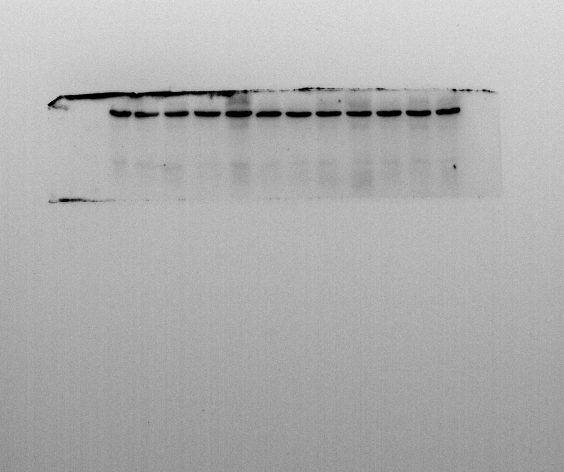

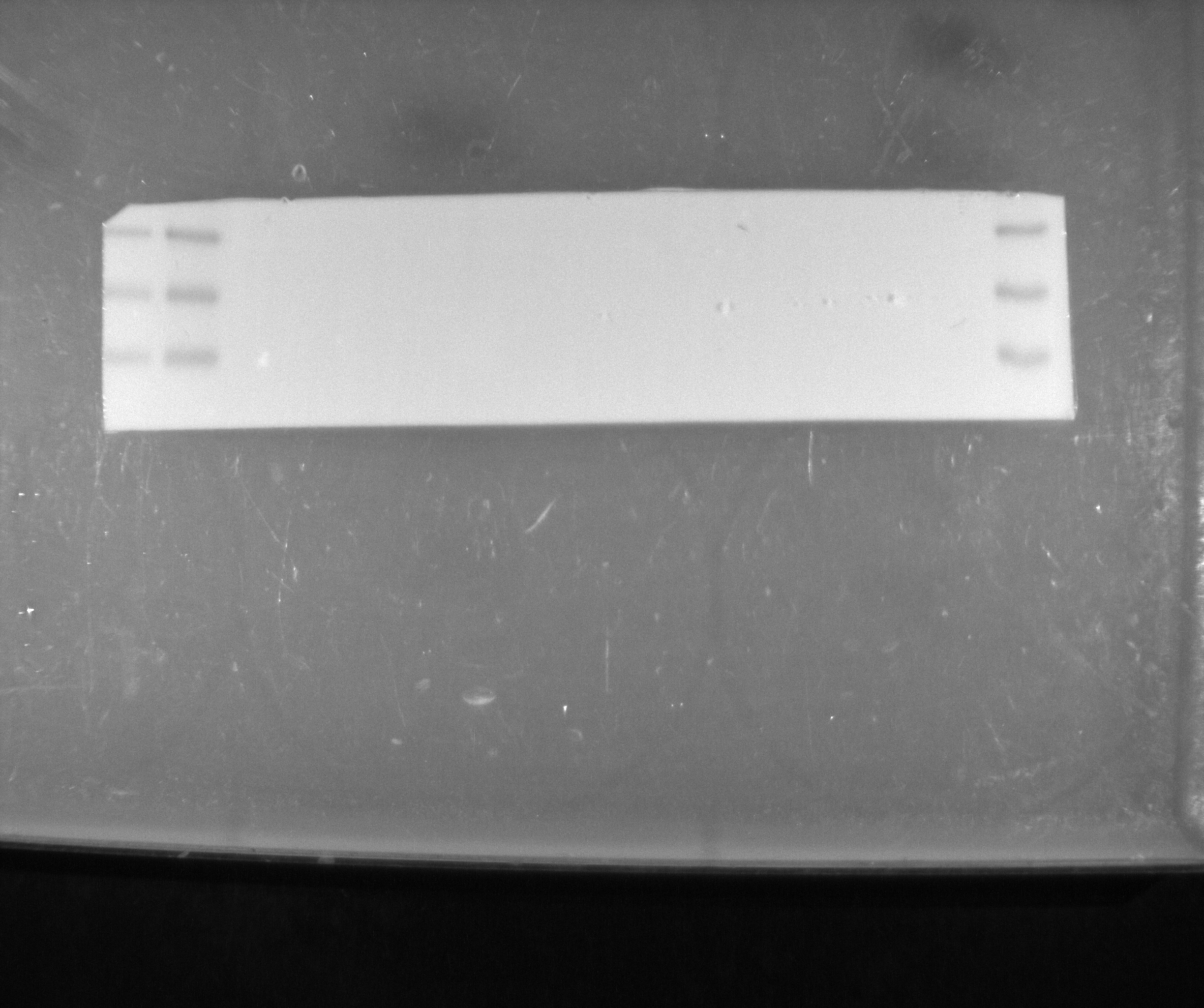


Fig.2H ADAM10 Fig.2H ADAM10 marker


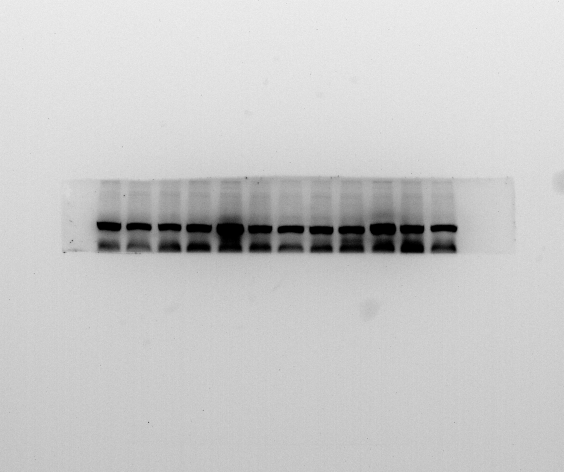

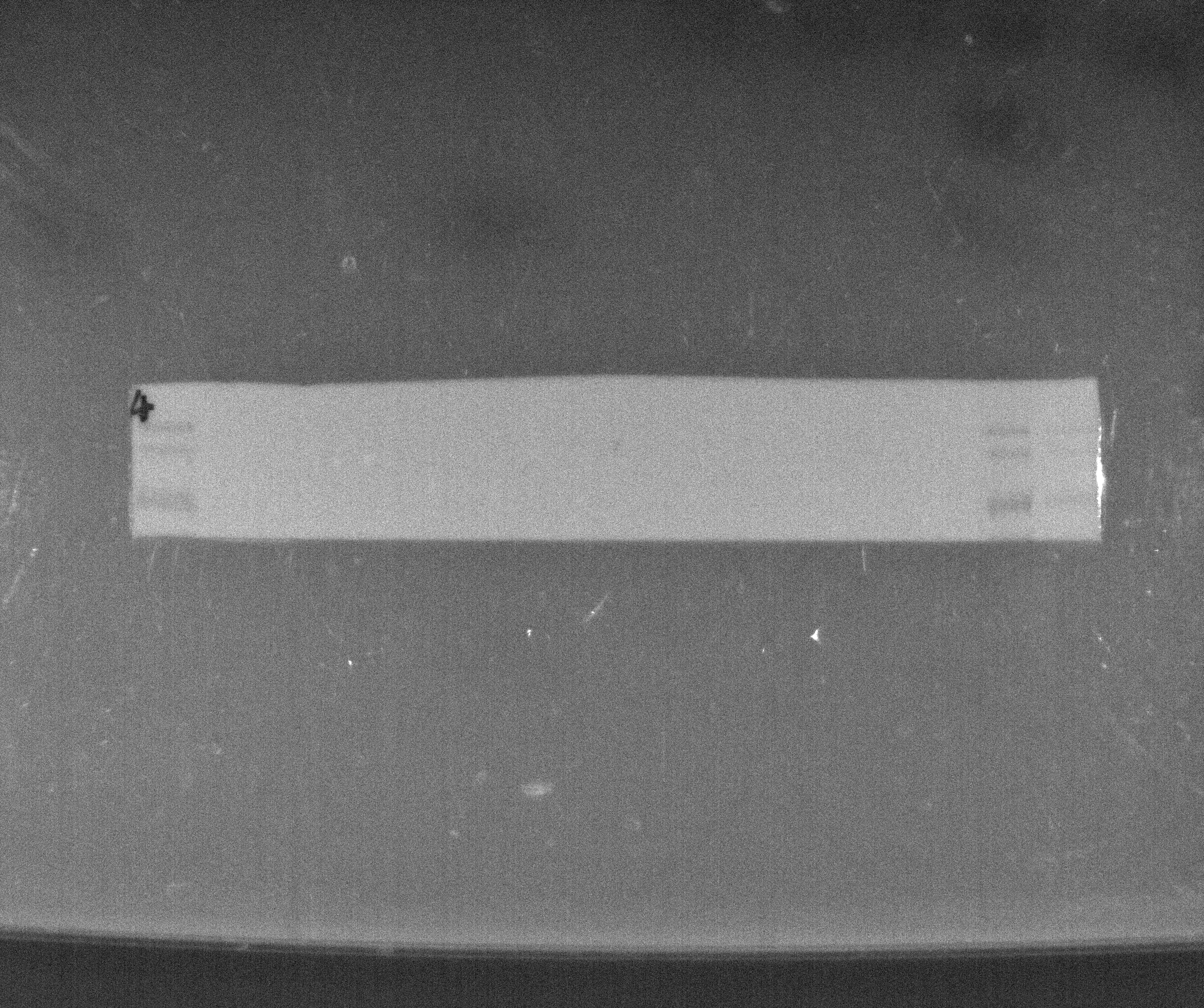


Fig.2H ACTIN (2023.3.24)





Fig.2K ADAM17 Fig.2K ADAM17 marker


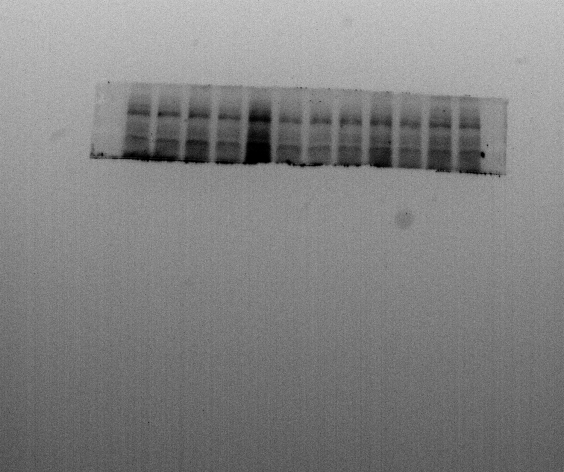

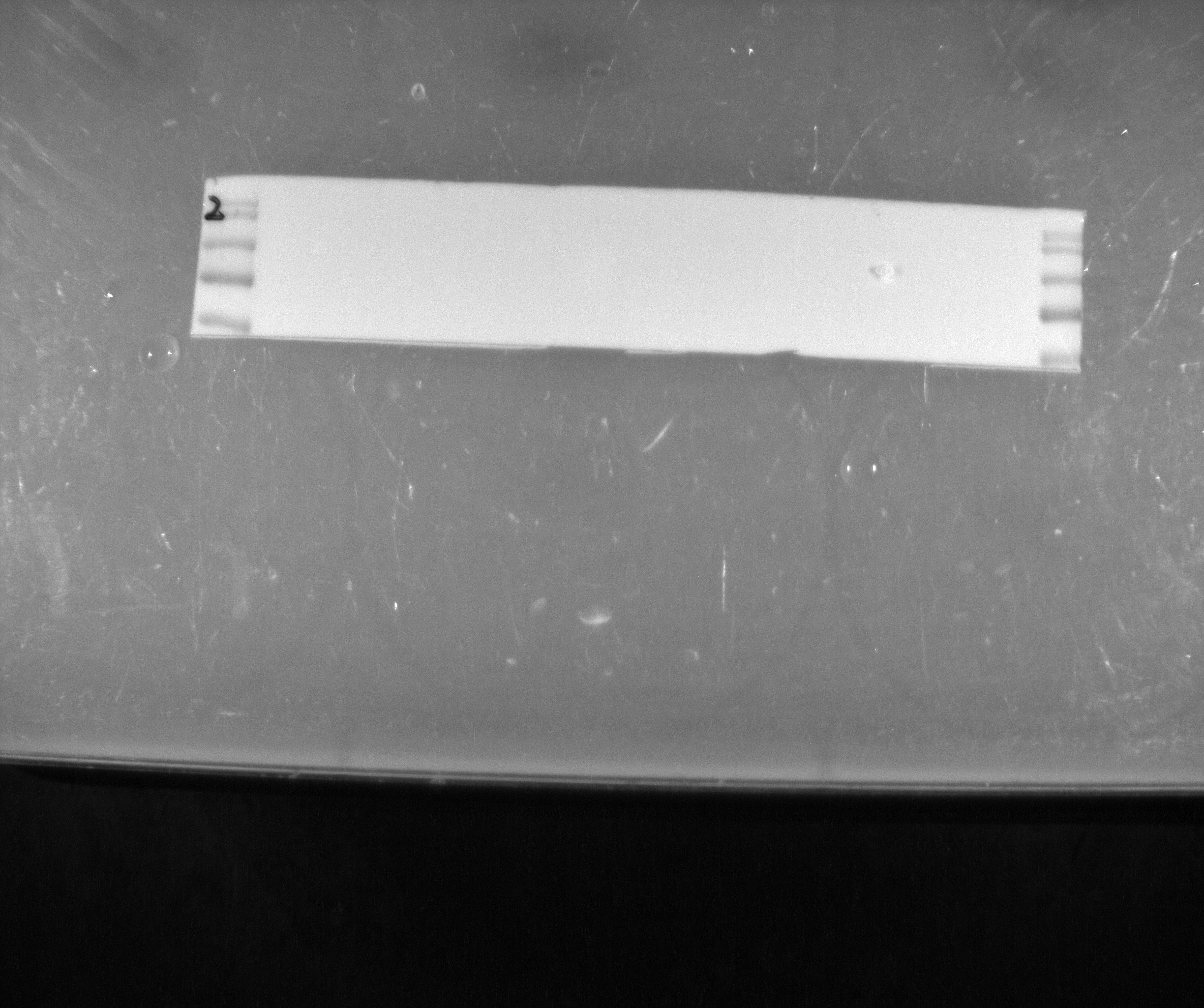


Fig.2K ACTIN (2023.3.24)





Fig.2K BACE1 Fig.2K BACE1 marker


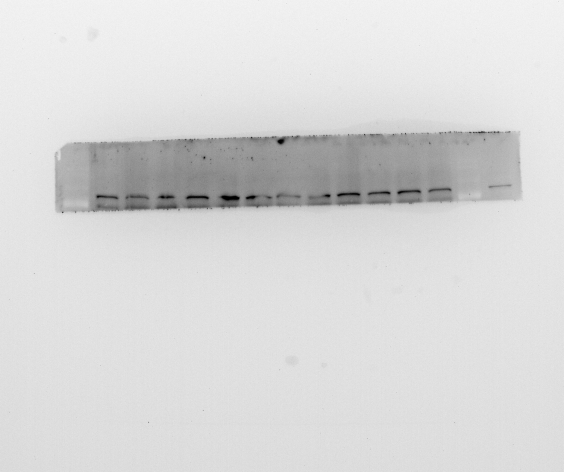

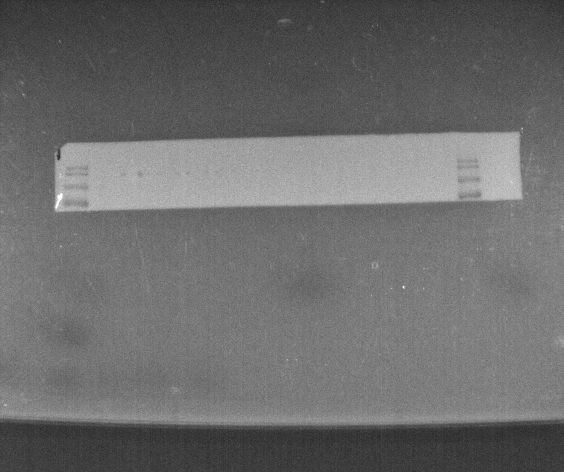


Fig.2K PS1 Fig.2K PS1 marker


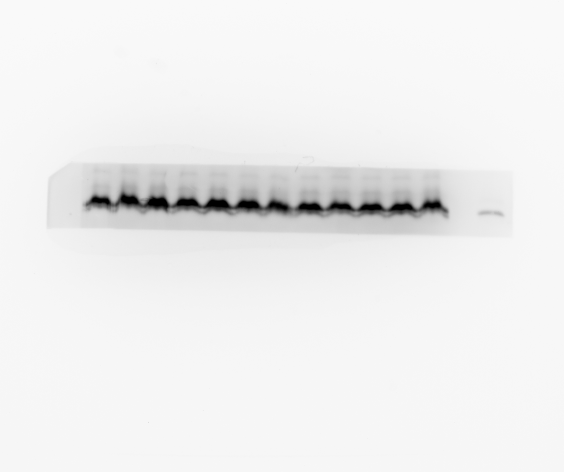

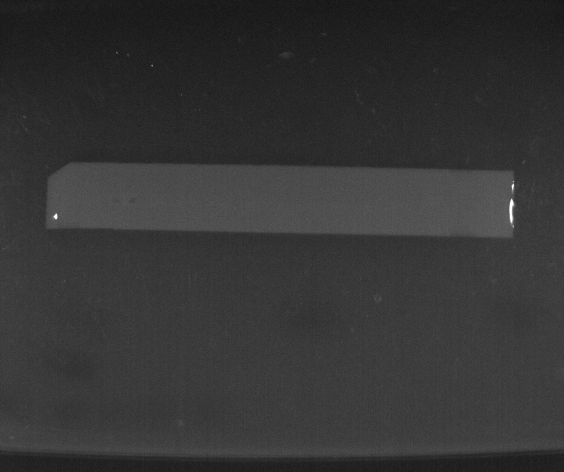


Fig.2K ACTIN Fig.2K ACTIN marker


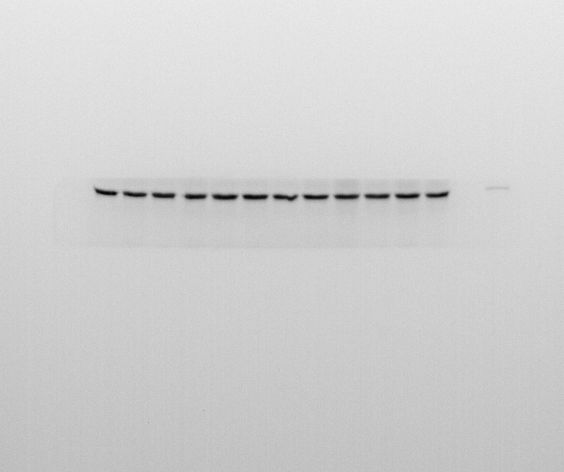

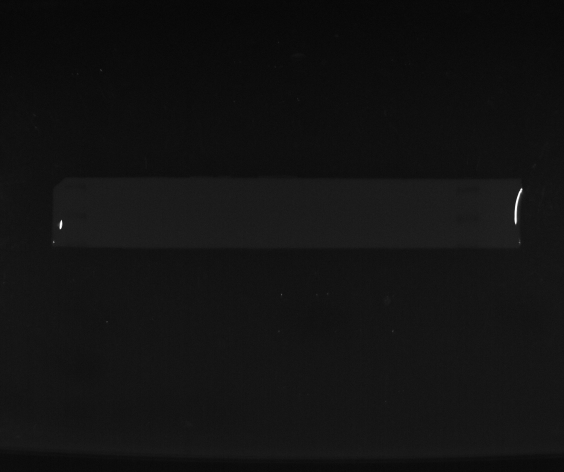

Supplement: Supplementary file 2 — Supplementary Material 2 [file 13195_2024_1527_MOESM2_ESM.docx]
