# Supplementary material for: Alpha-lipoic acid alleviates cognitive deficits in transgenic APP23/PS45 mice through a mitophagy-mediated increase in ADAM10 α-secretase cleavage of APP
Source: Alzheimers Res Ther. 2024 Jul 19;16:160. doi: 10.1186/s13195-024-01527-3 (PMC11264788; doi:10.1186/s13195-024-01527-3)

Fig.7A BNIP3L Fig.7A BNIP3L marker


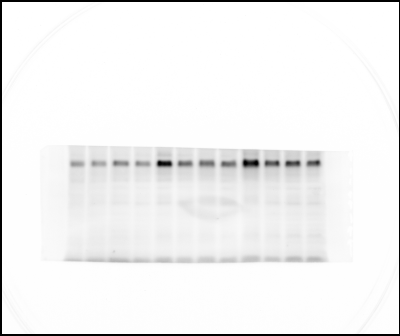

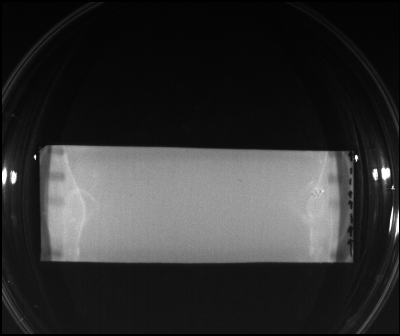


Fig.7A ADAM10 Fig.7A ADAM10 marker


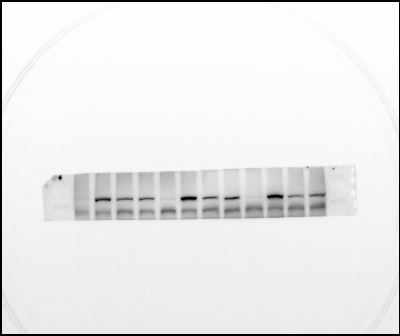

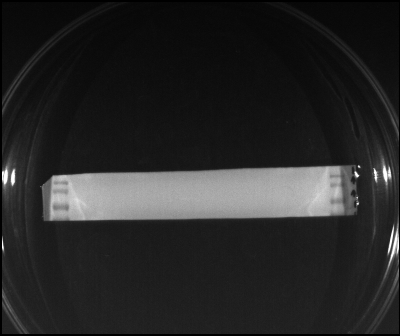


Fig.7A TUBLIN Fig.7A TUBLIN marker (2024.3.17)


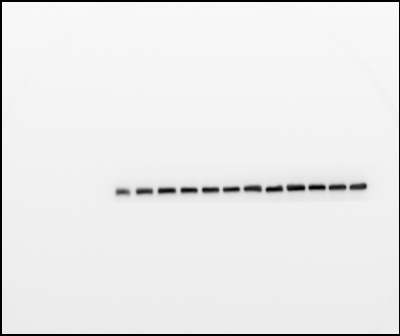

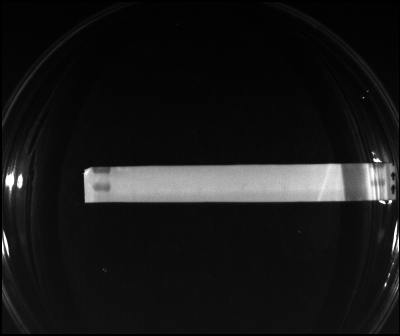


Fig.7D BNIP3L Fig.7D GAPDH


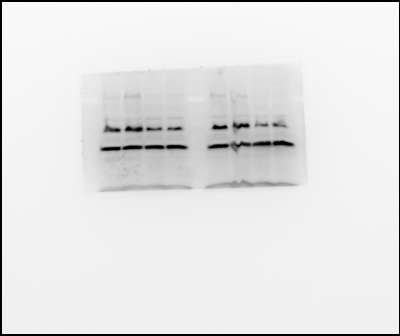

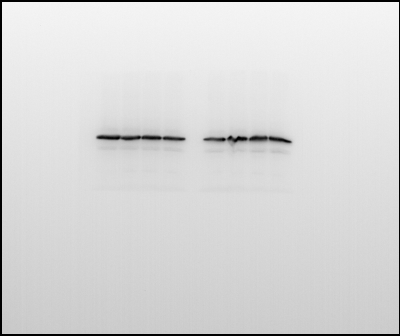


Fig.7D BNIP3L marker Fig.7D GAPDH marker (2023.6.29-30)


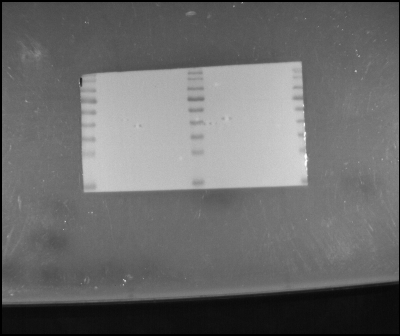

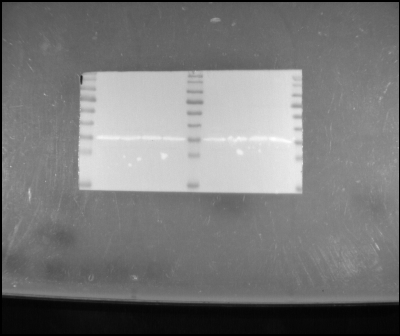


Fig.7D BNIP3L Fig.7D GAPDH


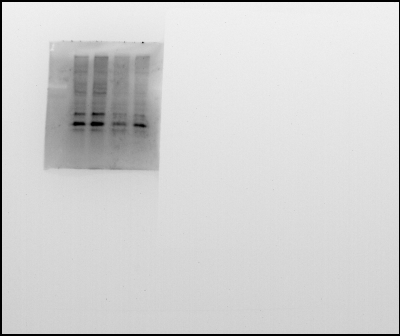

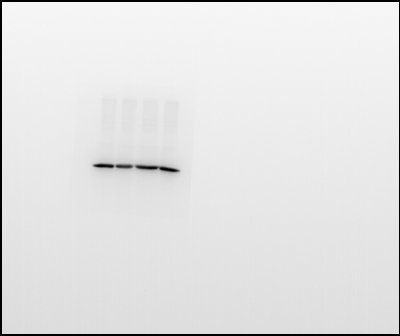


Fig.7D BNIP3L marker Fig.7D GAPDH marker (2023.6.29-30)


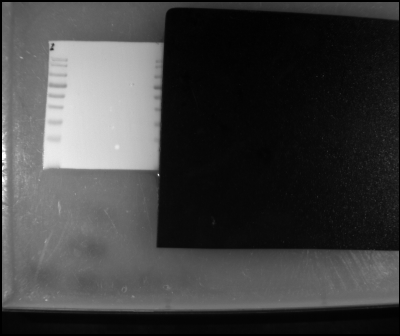

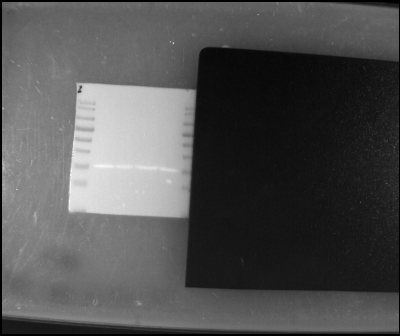


Fig.7D BNIP3L Fig.7D GAPDH


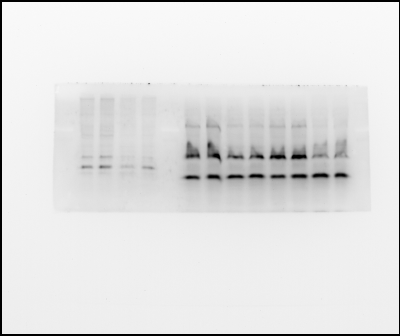

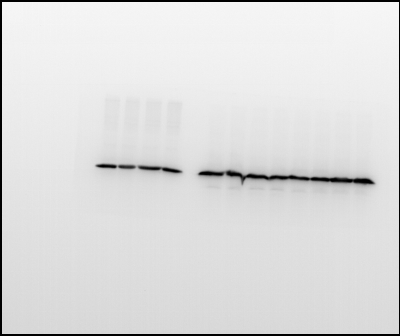


Fig.7D BNIP3L marker Fig.7D GAPDH marker (2023.6.29-30)


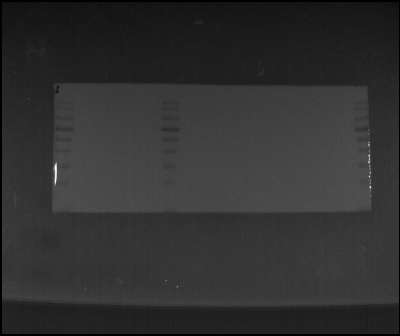

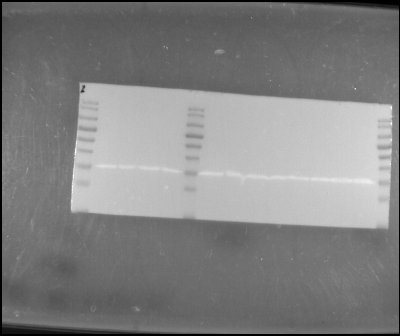


Fig.7E ADAM10 Fig.7E GAPDH


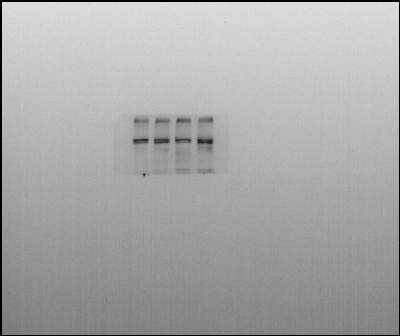

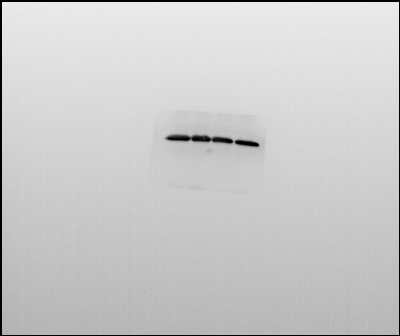


Fig.7E ADAM10 marker Fig.7E GAPDH marker (2023.5.17)


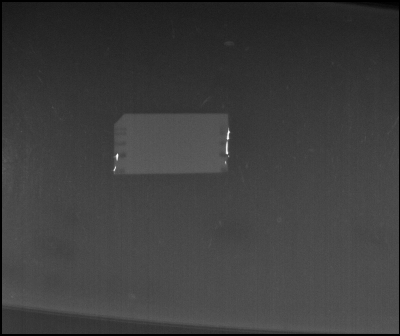

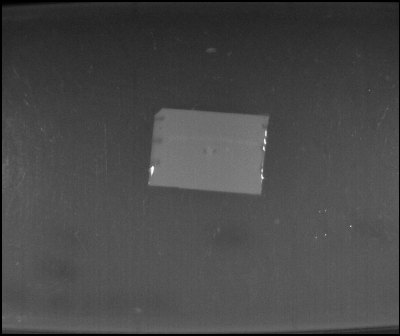


Fig.7E ADAM10 Fig.7E GAPDH


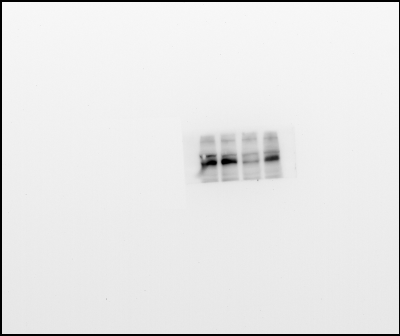

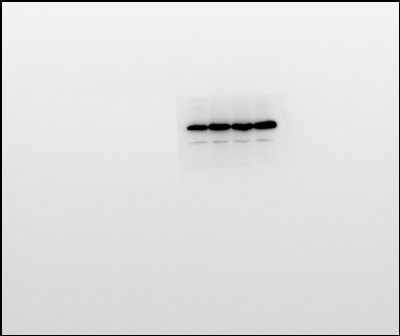


Fig.7E ADAM10 marker Fig.7E GAPDH marker (2023.5.24)


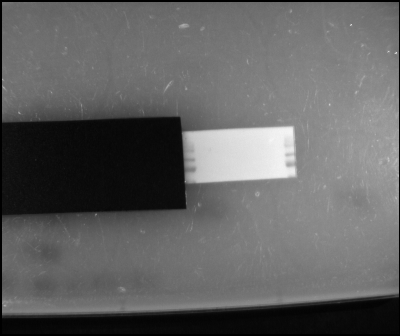

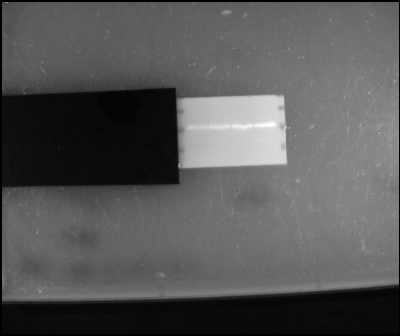


Fig.7E ADAM10 Fig.7E GAPDH


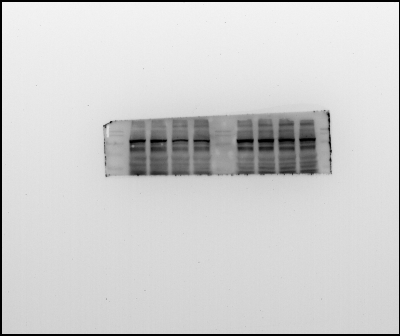

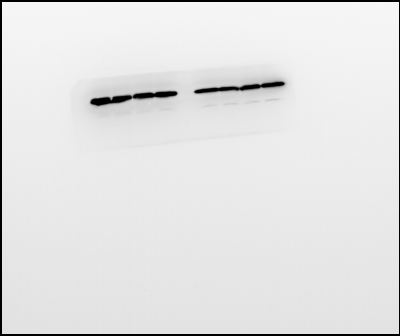


Fig.7E ADAM10 marker Fig.7E GAPDH marker (2023.5.25)


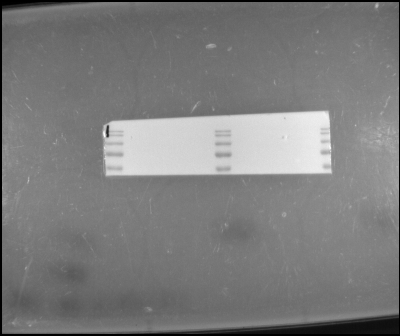

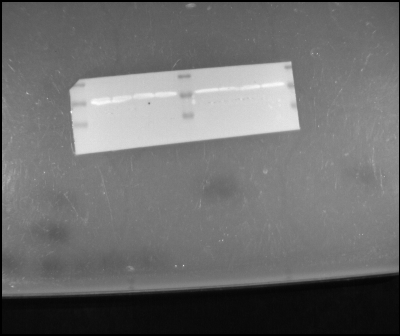


Fig.7F C83 Fig.7F GAPDH


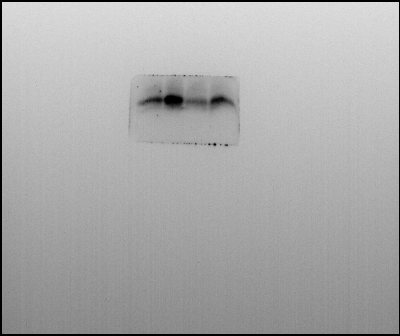

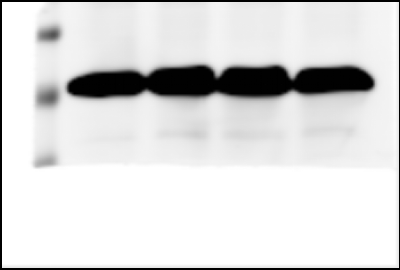


Fig.7F C83 marker (2023.5.19)


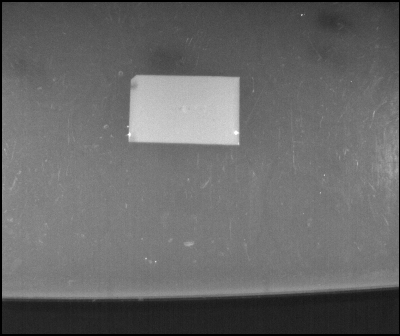


Fig.7F C83 Fig.7F GAPDH


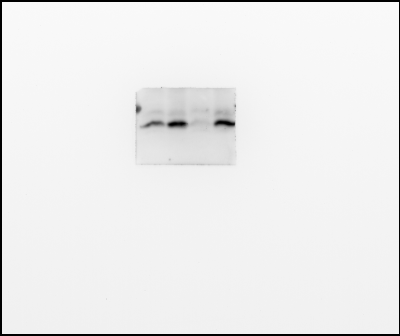

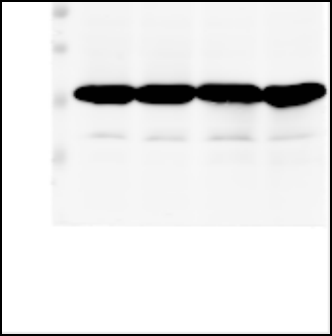


Fig.7F C83 marker (2023.5.24)


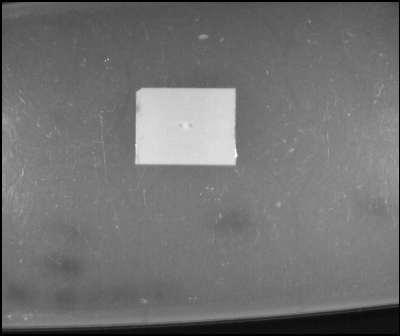


Fig.7F C83 Fig.7F GAPDH


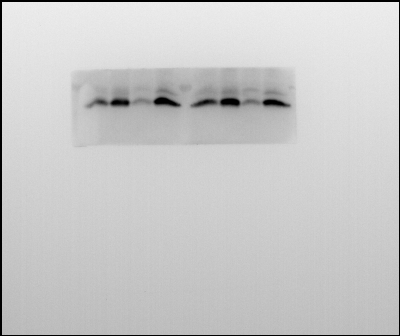

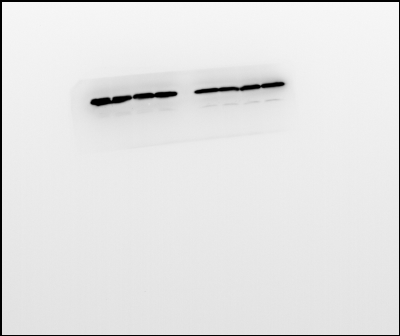


Fig.7F C83 marker Fig.7F GAPDH marker (2023.5.25)


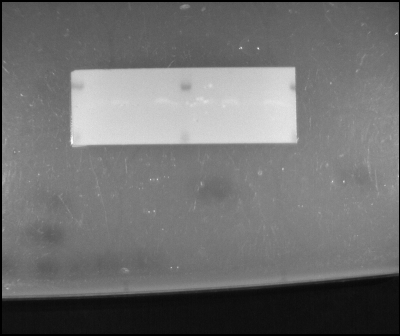

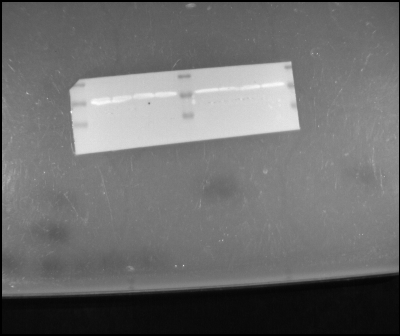

Supplement: Supplementary file 3 — Supplementary Material 3 [file 13195_2024_1527_MOESM3_ESM.docx]
