# Supplementary material for: Alpha-lipoic acid alleviates cognitive deficits in transgenic APP23/PS45 mice through a mitophagy-mediated increase in ADAM10 α-secretase cleavage of APP
Source: Alzheimers Res Ther. 2024 Jul 19;16:160. doi: 10.1186/s13195-024-01527-3 (PMC11264788; doi:10.1186/s13195-024-01527-3)

Fig.5A: P62 Fig.5A: GAPDH (2021.11.25)


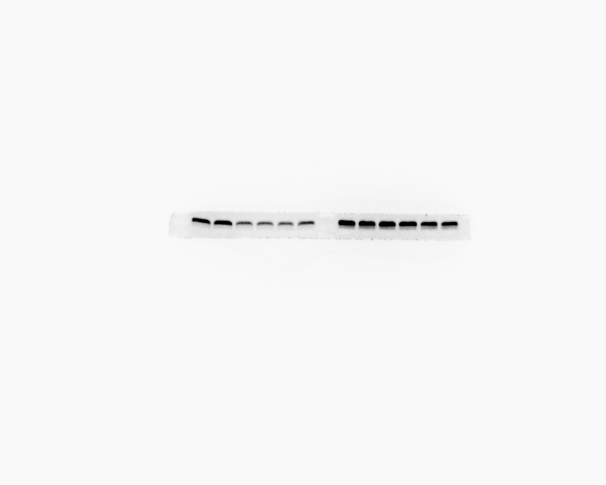

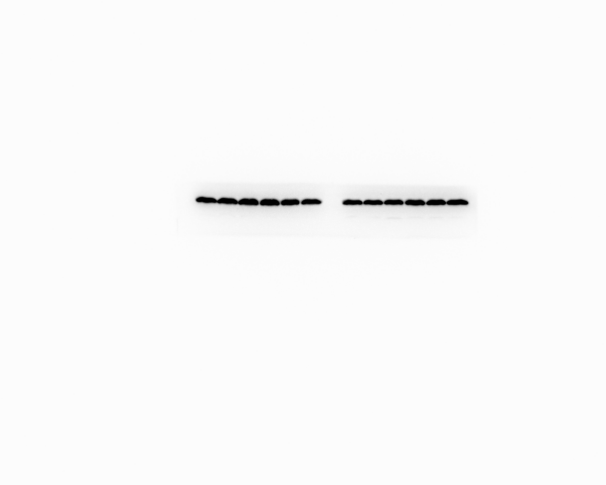


Fig.5A: P62 Fig.5A: GAPDH (2021.8.26)


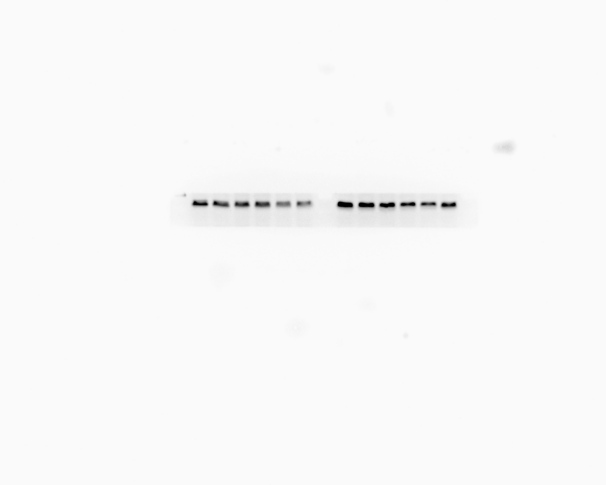

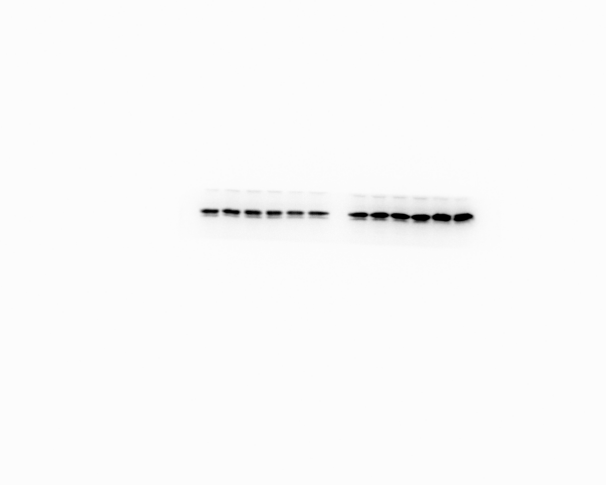


Fig.5A: P62 Fig.5A: GAPDH


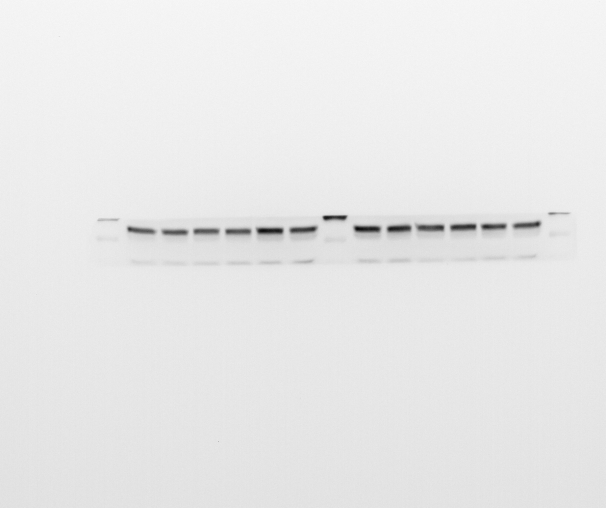

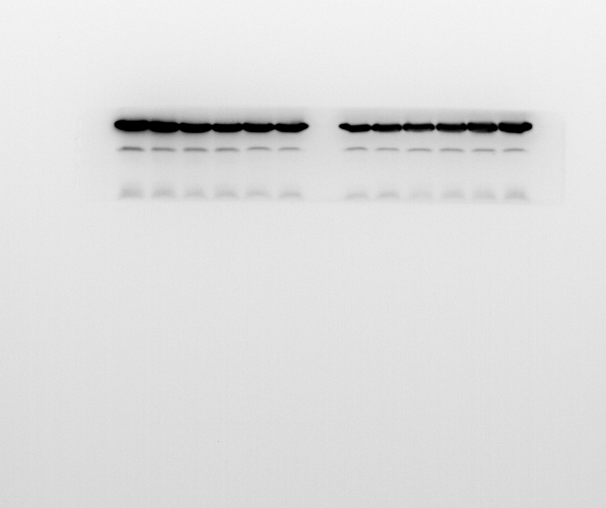


Fig.5A: P62 marker Fig.5A: GAPDH marker (2022.6.24)


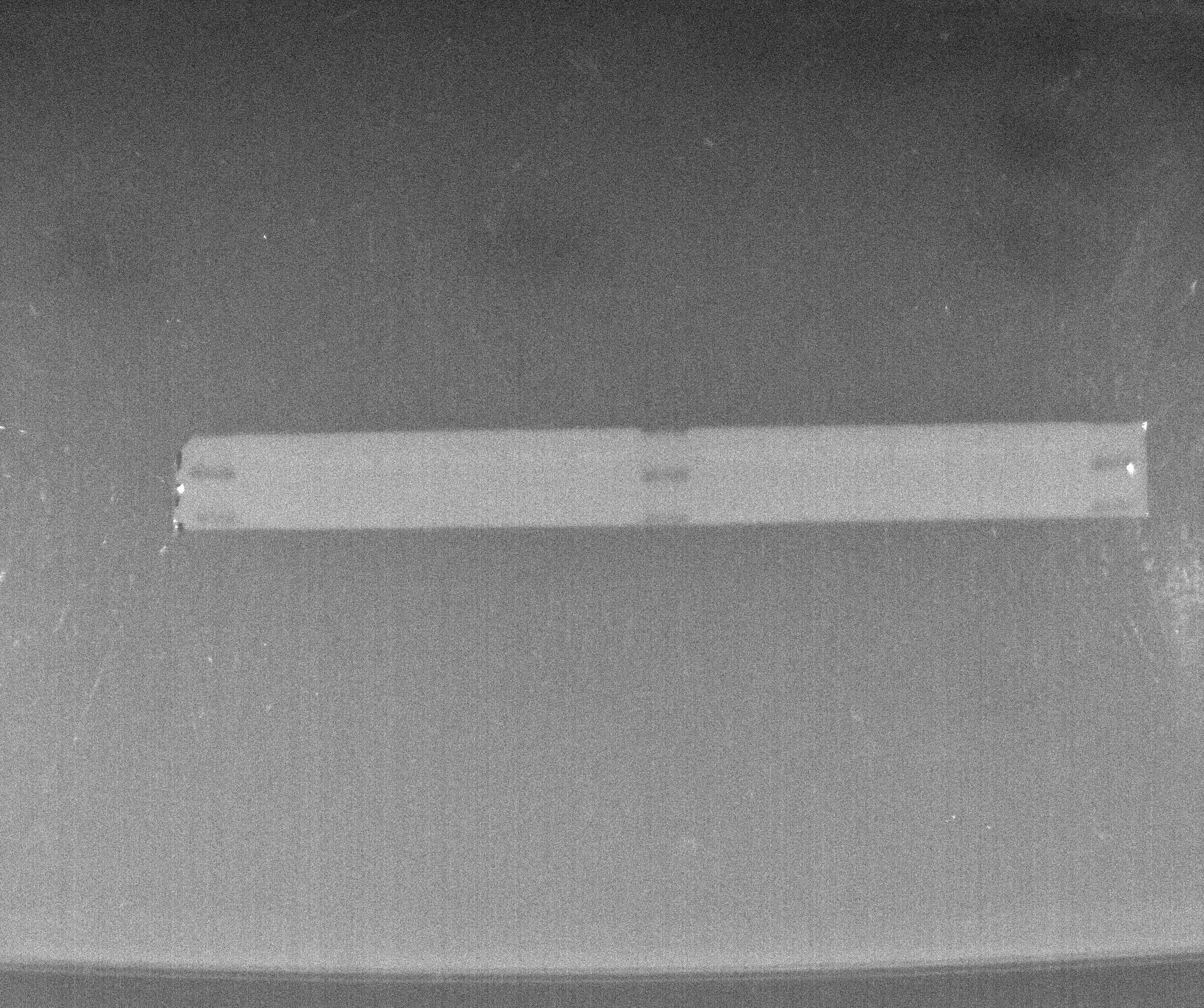

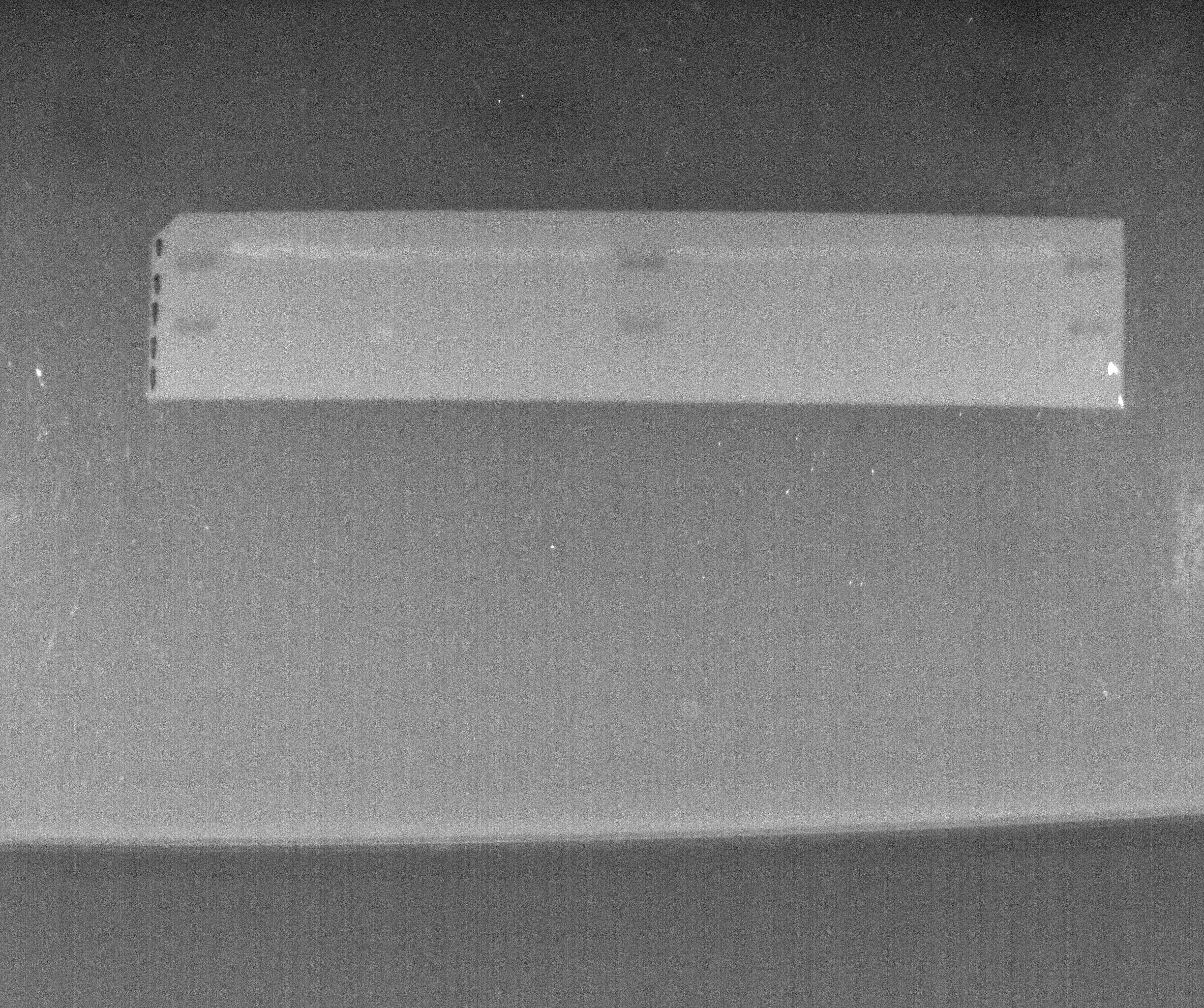


Fig.5A: P62 Fig.5A: ACTIN


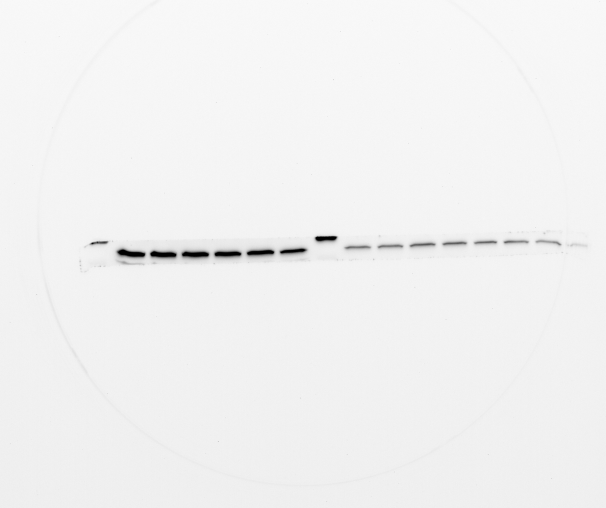

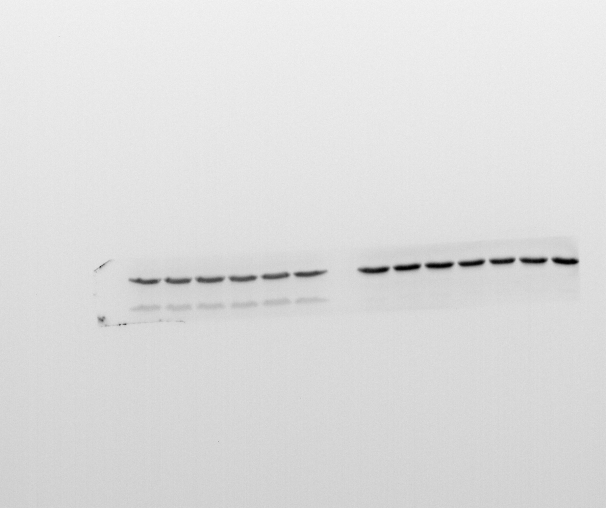


Fig.5A: P62 marker Fig.5A: GAPDH marker (2022.7.15)


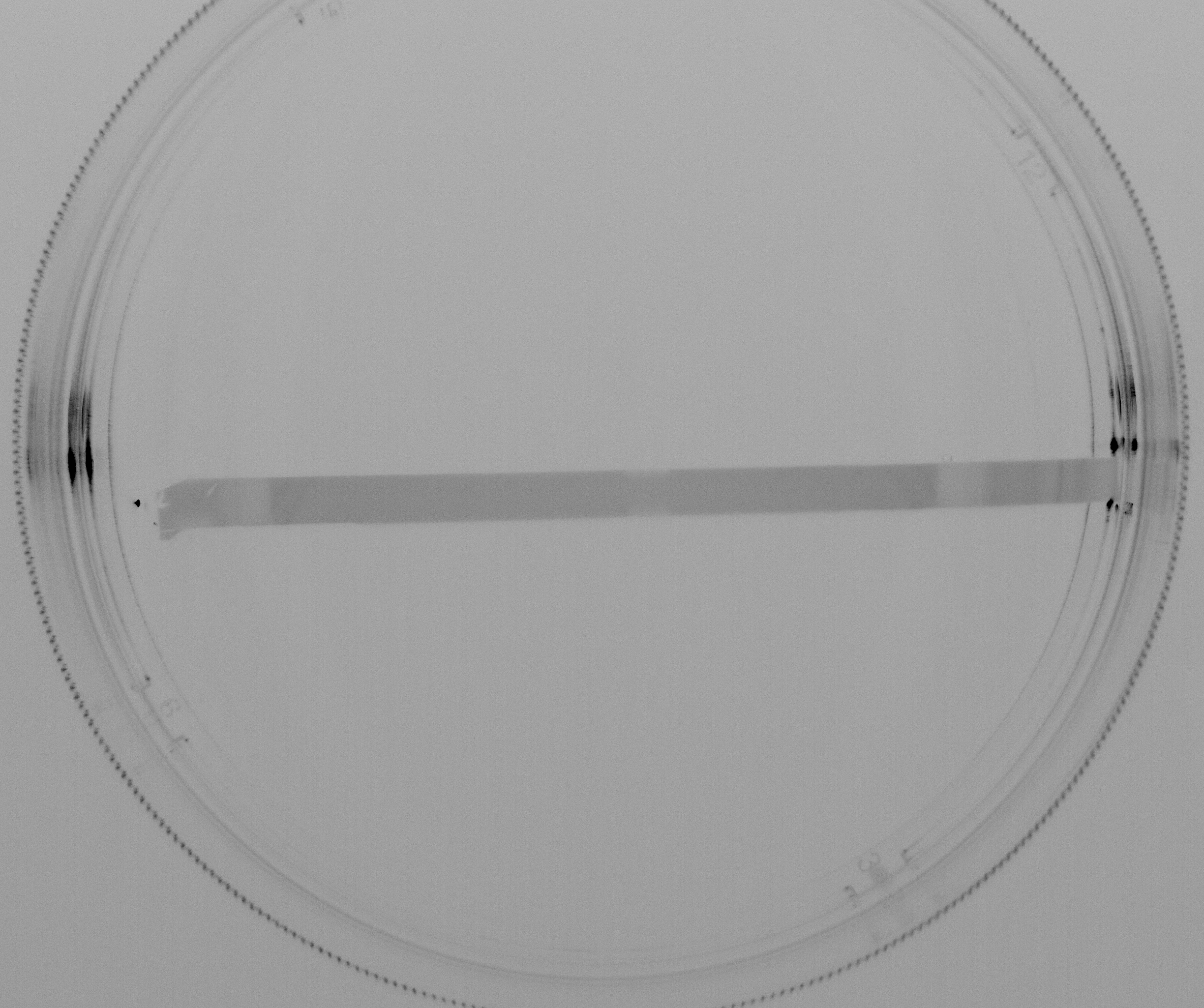

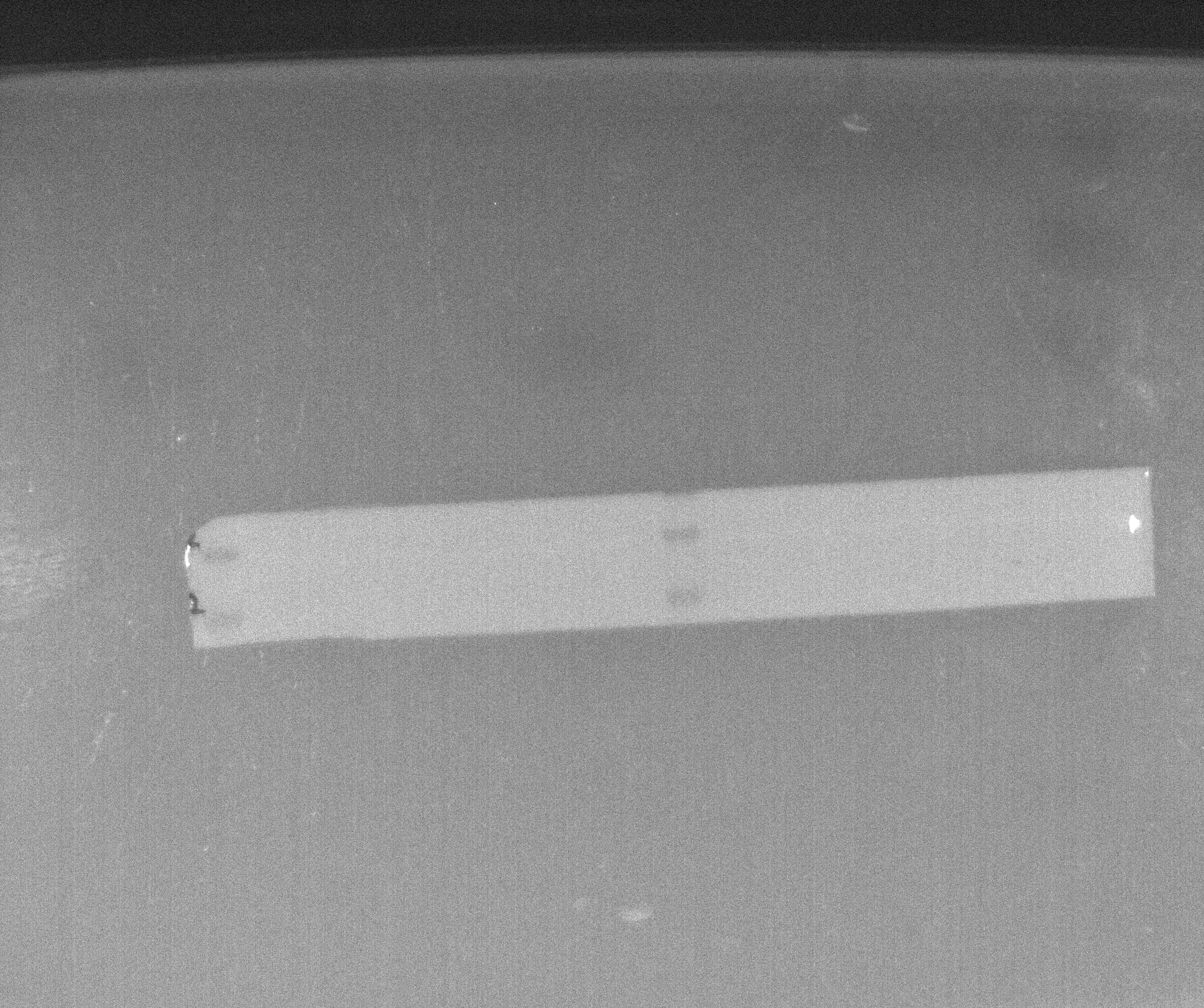


Fig.5B: LC3 Fig.5B: GAPDH (2021.6.25)


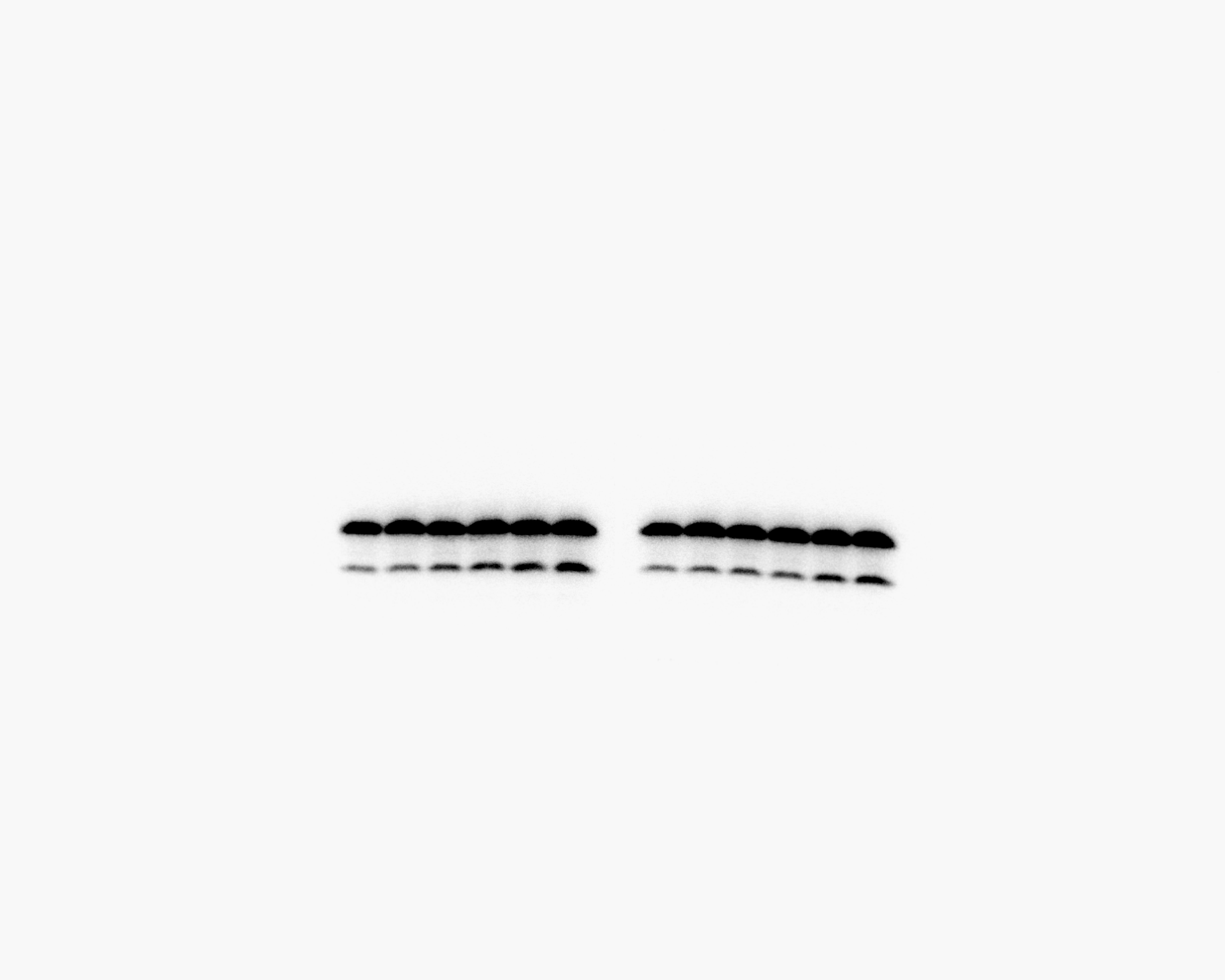

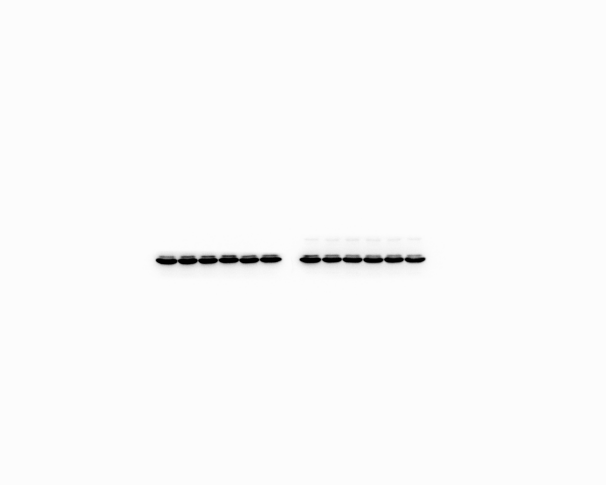


Fig.5B: LC3 Fig.5B: GAPDH (2021.9.23)


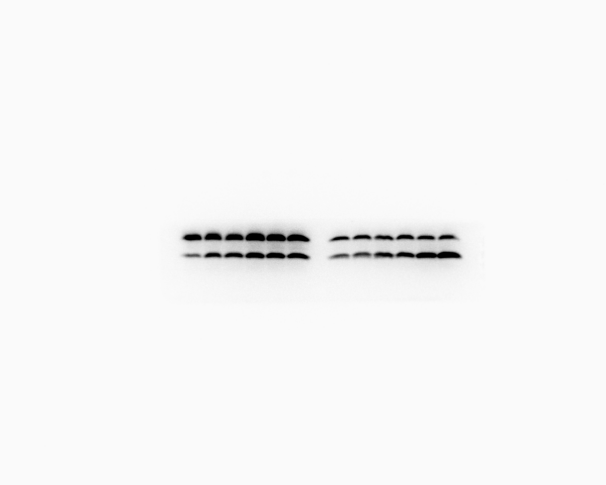

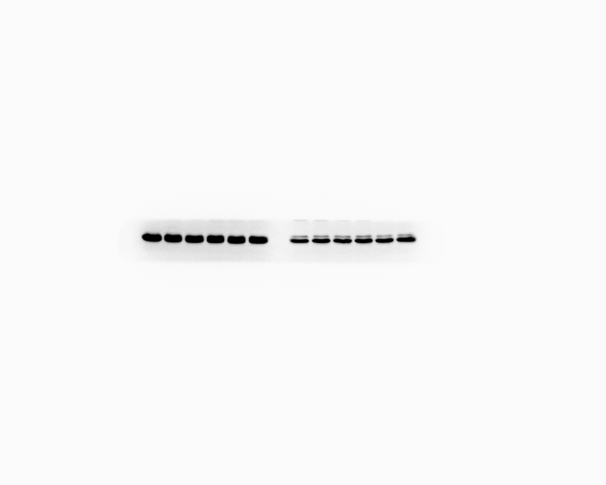


Fig.5B: LC3 Fig.5B: GAPDH (2021.11.11)


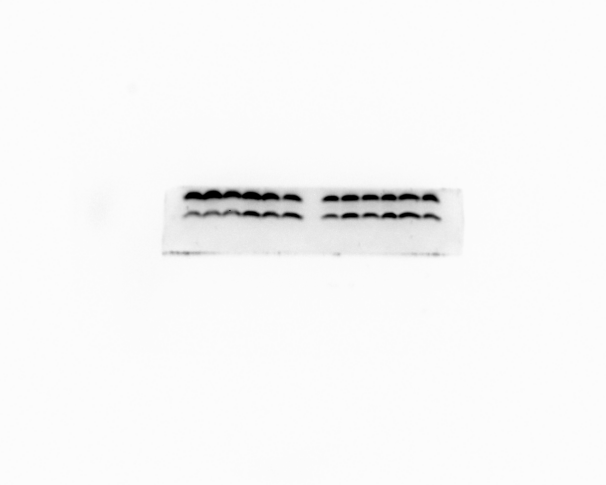

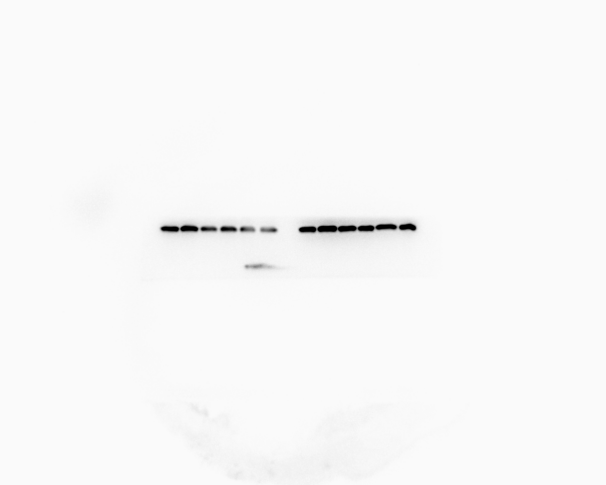

Supplement: Supplementary file 4 — Supplementary Material 4 [file 13195_2024_1527_MOESM4_ESM.docx]
