# Supplementary material for: Alpha-lipoic acid alleviates cognitive deficits in transgenic APP23/PS45 mice through a mitophagy-mediated increase in ADAM10 α-secretase cleavage of APP
Source: Alzheimers Res Ther. 2024 Jul 19;16:160. doi: 10.1186/s13195-024-01527-3 (PMC11264788; doi:10.1186/s13195-024-01527-3)
Supplement: Supplementary file 5 — Supplementary Material 5 [file 13195_2024_1527_MOESM5_ESM.docx]

Fig.3A: ADAM10	Fig.3A: GAPDH (2021.5.12)

Fig.3A: ADAM10	Fig.3A: ADAM10 marker

Fig.3A: GAPDH	Fig.3A: GAPDH marker (2022.10.20)

Fig.3A: ADAM10	Fig.3A: GAPDH (2021.7.20)

Fig.3A: ADAM10	Fig.3A: GAPDH (2021.8.26)

Fig.3A: ADAM10	Fig.3A: ADAM10 marker

Fig.3A: ACTIN	Fig.3A: ACTIN marker (2022.7.15)

Fig.3A: C83	Fig.3A: GAPDH (2021.4.8)

Fig.3A: C83	Fig.3A: ACTIN (2021.5.19)

Fig.3A: C83	Fig.3A: ACTIN (2021.8.29)

Fig.3A: C83	Fig.3A: GAPDH (2022.3.4)

Fig.3A: C83	Fig.3A: GAPDH (2021.11.30)

Fig.3B: APP	Fig.3B: APP marker

Fig.3B: GAPDH (2022.8.30)

Fig.3B: APP	Fig.3B: APP marker

Fig.3B: GAPDH	Fig.3B: GAPDH marker (2022.7.21)

Fig.3B: APP	Fig.3B: APP marker

Fig.3B: GAPDH	Fig.3B: GAPDH marker (2022.7.21)

Fig.3B: APP	Fig.3B: APP marker

Fig.3B: ACTIN (2022.10.30)

Fig.3B:PS1	Fig.3B: GAPDH (2021.4.3)

Fig.3B: PS1	Fig.3B: GAPDH (2021.4.7)

Fig.3B: PS1	Fig.3B: PS1 marker

Fig.3B: GAPDH	Fig.3B: GAPDH marker (2022.2.14)

Fig.3B: PS1	Fig.3B: PS1 marker

Fig.3B: GAPDH	Fig.3B: GAPDH marker (2022.7.8)

Fig.3B: PS1	Fig.3B: PS1 marker

Fig.3B: GAPDH	Fig.3B: GAPDH marker (2023.4.20)

Fig.3G: sAPPa	Fig.3G: sAPPa

Fig.3G: sAPPa marker and Fig.3G: sAPPa marker

Fig.3G: sAPPa

Fig.3G: sAPPa marker
